# Automated design of synthetic microbial communities

**DOI:** 10.1038/s41467-020-20756-2

**Published:** 2021-01-28

**Authors:** Behzad D. Karkaria, Alex J. H. Fedorec, Chris P. Barnes

**Affiliations:** 1 Department of Cell & Developmental Biology, University College London, London, WC1E 6BT UK; 2 UCL Genetics Institute, University College London, London, WC1E 6BT UK

**Keywords:** Microbial ecology, Multicellular systems, Population dynamics, Synthetic biology

## Abstract

Microbial species rarely exist in isolation. In naturally occurring microbial systems there is strong evidence for a positive relationship between species diversity and productivity of communities. The pervasiveness of these communities in nature highlights possible advantages for genetically engineered strains to exist in cocultures as well. Building synthetic microbial communities allows us to create distributed systems that mitigate issues often found in engineering a monoculture, especially as functional complexity increases. Here, we demonstrate a methodology for designing robust synthetic communities that include competition for nutrients, and use quorum sensing to control amensal bacteriocin interactions in a chemostat environment. We computationally explore all two- and three- strain systems, using Bayesian methods to perform model selection, and identify the most robust candidates for producing stable steady state communities. Our findings highlight important interaction motifs that provide stability, and identify requirements for selecting genetic parts and further tuning the community composition.

## Abstract (web summary)

In naturally occurring microbial systems, there is a positive relationship between species diversity and productivity of the community. Here the authors perform model selection to find potential amensal interactions that yield robust stable synthetic microbial consortia.

## Introduction

Traditionally, in biotechnology and synthetic biology, a microbe is engineered and grown as a monoculture to perform a particular function. Novel functionality is imparted by introducing heterologous genetic processes that would not normally be found in the organism. Non-orthogonal interactions between the introduced heterologous processes can cause the engineered function to behave in an unintended manner^1–3^, while the increased metabolic burden imposed can significantly slow growth rates and encourage selection of mutants^4^. Limited cellular resource availability and unforseen interactions can cause the host organism and the introduced circuits to behave differently when expressed alongside one another^5–7^. Using microbial communities would enable us to allocate functional components between subpopulations of cells, creating physical barriers that insulate processes from one another and distribute the burden of heterologous expression between members of the community^7^. This allows us to scale complexity in a manner that could not be achieved under the limitations of a monoculture. In natural environments, we observe mixed-species microbial communities that exhibit competitive advantages over monocultures in productivity, resource efficiency, metabolic complexity and resistance to invasion^8,9^. Being able to predictably and reproducibly construct microbial communities for synthetic biology or biotechnology applications would allow us to harness these advantages.

The maintenance and control of microbial communities comes with its own challenges. Competitive exclusion occurs when multiple populations compete for a single limiting resource (in the absence of other interactions); a single population with the highest fitness will drive the others to extinction^10^. Evidence from microbial ecology has shown us that stability can arise through feedback between subpopulations. Cooperative and competitive interactions are both important for integrating feedback that can stabilise communities by manipulating growth or fitness of the subpopulations^11–16^. Synthetic microbial communities have been built using quorum sensing (QS) systems to regulate processes that manipulate the growth rate or fitness of a population. Fitness can be manipulated by the expression of lysis proteins, metabolic enzymes, toxins and anti-microbial peptides (AMPs)^14,17–23^. Here, we focus on the use of bacteriocins to manipulate subpopulation fitness. Bacteriocins are gene-encoded AMPs that can be used to directly suppress the growth rate of a sensitive population^24^. They are exported into the extracellular environment, and generally use “Trojan horse” strategies to enter and kill sensitive strains. Expression of immunity genes provides protection against the bacteriocin, and can be expressed separately or in conjunction with the bacteriocin^25^. A single expressed bacteriocin can impact the growth of multiple other strains in the system, as opposed to intracellular toxins which require all strains to be engineered. Bacteriocins also offer variable spectrums of sensitivity, enabling broad or narrow targeting of microbial species^24^. Previously, we have demonstrated the use of bacteriocin MccV to improve plasmid maintenance in a population^26^ and for building stable cocultures that overcome competitive exclusion^66^. Other bacteriocins, such as nisin, have also been used to produce stable communities^23^.

Predicting how a system will behave before implementation is essential for the efficient use of lab resources and fully understanding the interactions that occur^27^. System design by intuition alone becomes increasingly challenging when dealing with multi-level interactions. We can use model selection to compare a set of candidate models and identify the most promising designs^28^. We have previously performed model selection and parameterisation using Approximate Bayesian computation with sequential Monte Carlo sampling (ABC SMC)^29^ to design robust genetic oscillators^30^ and multistable genetic switches^31^. Similar approaches have been used to compare the ability of genetic parts to produce logic gate behaviours^32^ and to design regulatory networks from databases of characterised parts^33,34^. Automated circuit design has the potential to greatly improve the engineering process in synthetic biology.

Here, we build upon computational circuit design in synthetic biology, presenting automated synthetic community design. Our workflow automatically generates candidate systems from a set of parts which can be used to engineer a community. We use ABC SMC to perform model selection, identifying candidate systems that have the highest probability of producing stable communities in a chemostat bioreactor. Using these methods we reveal the optimal designs for two-strain and three-strain systems. This workflow also allows us to derive fundamental design principles for building stable communities and reveals critical parameters to control the community composition.

## Results

### Automated synthetic microbial Community Designer (AutoCD) workflow

**Fig. 1:**
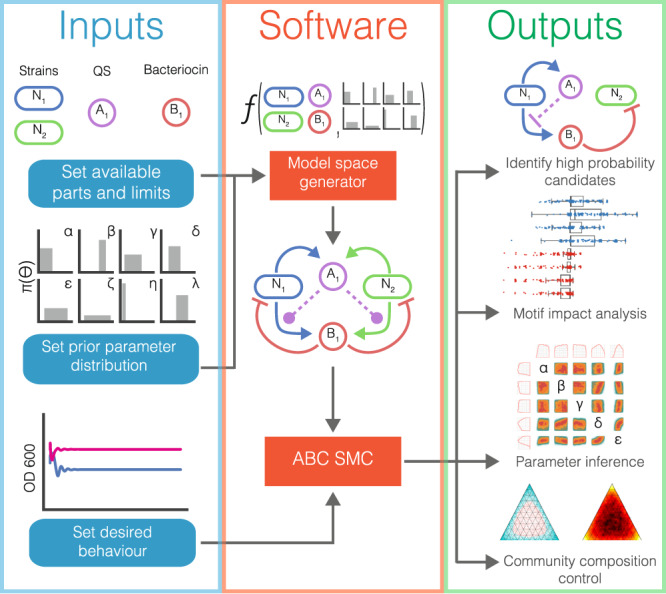
Overview of AutoCD pipeline. Model selection workflow begins with definition of available parts and prior parameter distributions used to generate system models from all the possible interactions. We use ABC SMC to perform model selection for the desired population behaviour. The outputs of ABC SMC provide us with community designs, insight into underlying motifs, parameter requirements and information on composition tunability.

Figure 1 illustrates AutoCD, the workflow developed and applied in this study. First, we set the available parts which can be used to build a stabilising system in a chemostat environment. This consists of the number of strains (*N*), bacteriocins (*B*), and QS systems (*A*). Any QS system can regulate the expression of any bacteriocin in the system by induction or repression. Strains in all models are dependent upon a single nutrient resource (*S*), which is consumed by strains and replenished through dilution of the chemostat with fresh media. Importantly, all models therefore include nutrient-based competition between subpopulations. Uniform distributions are used to encode our prior knowledge of biochemical rate parameters informed by literature, describing each part and their interactions with one another (Table 1). The priors used are broad to allow the full range of possible part characteristics; in scenarios where the parts have already been selected and characterised, the prior parameters can be constrained. The available parts and prior parameter distributions serve as inputs to the model space generator, which conducts a series of combinatorial steps to produce all possible genetic circuits. The model space generator then builds unique combinations of strains expressing different genetic circuits, where each combination is a candidate model. Filtering steps remove unviable, redundant and mirror systems, yielding a set of unique candidates to be assessed. The model space generator produces an ordinary differential equation (ODE) model for each system in the context of the chemostat environment, and these models form our prior model space (for details, see the “Methods“ section).

**Table 1:** Prior distributions for both two and three strain systems. Constant parameters have the same min and max value. ${K}_{{A}_{y}{B}_{z}}$, *K*_*ω*_, ${K}_{{A}_{y}}$ and ${KB}_{\mathrm{max}}z$ are sampled from log uniform distributions. The remaining parameters are sampled from uniform distributions.

Parameter/state variable	Description	Prior (min)	Prior (max)	Units	Citation
*Parameters*					
*C*_*N*_	OD to cell number scaling factor	1e9	1e9	None	N/A
*C*_*B*_	Microcin scaling factor	1e−9	1e−9	None	N/A
*C*_*A*_	QS scaling factor	1e-9	1e−9	None	N/A
*D*	Dilution rate	0.01	0.2	h^−1^	N/A
${K}_{{A}_{y}{B}_{z}}$	Half-maximal QS promoter activation/repression from *A*_*y*_ to *B*_*z*_	1e−9	1e−6	M	^81^
*K*_*x*_	Monod’s half-saturation constant	3.9e−5	3.9e−5	M	^82^
*K*_*ω*_	Half-saturation killing constant	1e−7	1e−6	M	^83,84^
*S*_0_	Substrate concentration of input media (0.4% glucose)	0.02	0.02	M	M9 media
*γ*	*E. coli* substrate yield	1e11	1e11	cell M^−1^	^85^
${k}_{{A}_{y}}$	Production rate of AHL per cell	1e−22	1e−15	M h^−1^	^86^
$K{B}_{\max }z$	Maximal expression rate of microcin	1e−22	1e−15	M h^−1^	^6^
${\mu }_{{x}_{\max }}$	Maximum growth rate	0.4	3	h^−1^	^39,40^
*n*_*z*_	Hill coefficient AHL induced expression	1	2	M	^81^
*n*_*ω*_	Hill coefficient for killing	1	2	M	^81^
${\omega }_{\max }$	Maximum rate of bacteriocin killing	0.5	2.0	M^−1^ h^−1^	^83,84,87^
*Initial state variable*					
*N*	OD of strain	0.01	0.5	OD	N/A
*S*	0.4% glucose concentration	0.02	0.02	M	N/A
*B*	Microcin concentration	1e−81	1e−81	M	N/A
*A*	QS concentration	1e−10	1e−10	M	N/A

The final input is a mathematical description of the objective population behaviour, a stable steady state. We use three distance functions (*d*_1_, *d*_2_, *d*_3_) to describe how far away a simulation is from the objective stable steady state (Eq. (1)). *d*_1_ is the final gradient of a strain population (*N*_*x*_), capturing the most fundamental characteristic of stable steady state, where the population level of a strain is unchanging. *d*_2_ is the standard deviation of a population, quantifying unstable behaviours such as oscillations, favouring simulations that reach stable steady state quickly. *d*_3_ is the reciprocal of the strain population at the end of the simulation, allowing us to define a minimum population density. Given the three distances, *ϵ*_*F*_ defines thresholds below which a simulation meets the requirements of our stable steady state objective. The distances of all strain populations in a simulation must be below these thresholds to satisfy the objective behaviour. ${\epsilon }_{{F}_{1}}$ was chosen to match the error tolerance of the ODE solver and ${\epsilon }_{{F}_{2}}$ threshold was chosen through qualitative assessment of simulation data to define a practical threshold for what stable steady state simulations should look like. ${\epsilon }_{{F}_{3}}$ is set to ensure all populations have a minimum final OD of 0.001, chosen for what could be realistically measured using flow cytometry. The posterior distribution is made up of simulations where the distances for each strain population are less than the *ϵ*_*F*_ thresholds (Eq. (2)).

ABC SMC performs model selection on the model space for the objective defined by these distance functions and *ϵ*_*F*_. A particle is a sampled model and associated parameters. ABC SMC initially samples particles from the prior distributions with an unbounded distance threshold. Particles are propagated through intermediate distributions, gradually reducing the distance thresholds until they equal *ϵ*_*F*_ (see the “Methods” section). ABC SMC provides an estimation of model and parameter space posterior probabilities for the given prior distributions and the objective behaviour. We can use the outputs of ABC SMC to help us design synthetic communities and chemostat settings in the lab.

Distance functions:

1 $${d}_{1}({N}_{x}) 	=| {{\Delta }}{N}_{x}(t-1)| \\ {d}_{2}({N}_{x}) 	=\sigma ({N}_{x})\\ {d}_{3}({N}_{x}) 	=\frac{1}{{N}_{x}(t-1)}$$

Distance thresholds:

2 $${\epsilon }_{F}	= \{1{{\mathrm{{e}}}}^{-9},0.001,1000\}\\ 	\quad{\,\,}{d}_{1}\, <\, {\epsilon }_{{F}_{1}}\\ 	\quad{\,\,}{d}_{2}\, <\, {\epsilon }_{{F}_{2}}\\ 	\quad{\,\,}{d}_{3}\, <\, {\epsilon }_{{F}_{3}}$$

### Designing two-strain cocultures that achieve steady state

**Fig. 2:**
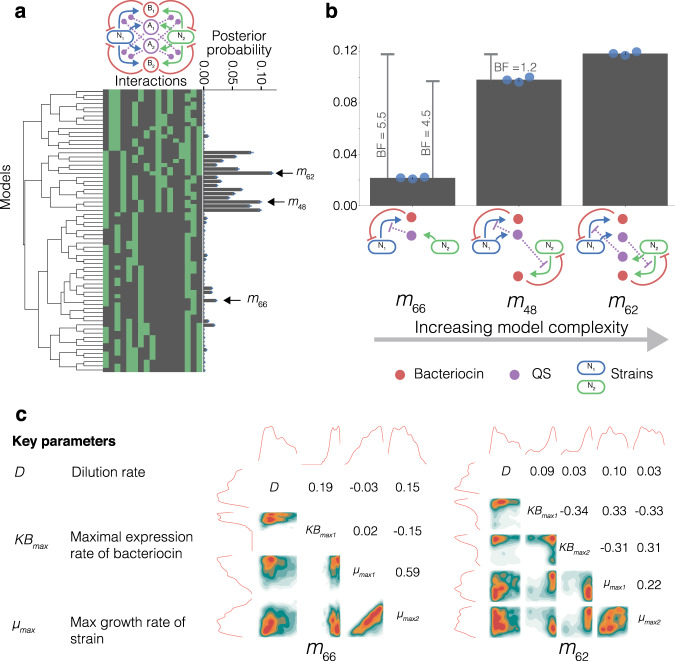
Output of AutoCD for the two-strain stable steady state objective. **a** Dendrogram generated by hierarchical clustering of the adjacency matrices for each model in the two-strain model space. All possible interactions are shown in the network illustration. Each column of the heatmap represents a possible interaction between state variables, where green indicates the interaction exists for the model and black indicates absence of the interaction. The bar chart shows the mean posterior probability of each model. The scatter points indicate the posterior probability of each replicate and the error bars indicate the standard deviation. *n* = 3, where each replicate consists of 180,000 accepted particles. **b** Shows the mean posterior probability of three models with the highest posterior probability when subsetted for number of parts expressed in order of increasing complexity (2, 3 and 4 parts). The scatter points indicate the posterior probability of each replicate and the error bars indicate the standard deviation. *n* = 3, where each replicate consists of 180,000 accepted particles. Bayes factors (BF) are shown for pairwise comparison of the three models. Model schematics show the interactions between strains (blue and green), bacteriocin (red) and QS molecules (purple). **c** Posterior parameter distributions of several tunable parameters in *m*_66_ and *m*_62_. The top and left plots show 1D density distributions of each parameter, central distributions are 2D density distributions for each pair of parameters. Pearson correlation coefficients are shown on the top side of the diagonal for each parameter pair.

Here we apply AutoCD to the design of a stable steady state coculture containing two strains. In Fig. 2 we define a model space consisting of two strains (*N*_1_, *N*_2_), two bacteriocins (*B*_1_, *B*_2_) and two QS systems (*A*_1_, *A*_2_). We set model space limits to enable feasible experimental implementation, allowing expression of up to one QS per strain and expression of up to one bacteriocin per strain. Each strain can be sensitive to up to one bacteriocin. Given these conditions, the model space generator yields 69 unique two-strain models (*m*_0_,*m*_1_...*m*_68_). These 69 models serve as a uniform prior model space upon which we perform model selection using ABC SMC (see Supplementary Fig. 4 for visualisation of each candidate model). From the available genetic parts, there are 17 possible interaction options that could exist between state variables in each candidate model. We perform hierarchical clustering on the interactions present in each model, grouping models based on the similarity of their interactions. This clustering is visualised as a dendrogram in Fig. 2a. ABC SMC approximates the posterior probability of each model for the stable steady state objective, indicating how effective the candidate system is in producing a stable steady state. *m*_62_ has the highest posterior probability, and is therefore the system which most robustly produces stable steady state (Fig. 2a). *m*_62_ consists of two strains exhibiting a cross-protection mutualism relationship^35^. Each strain expresses an orthogonal QS molecule that represses the expression of a self-limiting (SL) bacteriocin in the opposing strain (Fig. 2b). In the absence of the opposing strain, the SL bacteriocin is expressed freely. This creates an interdependence between the two strains where the extinction of one strain would result in the extinction of the other. This closed feedback loop is a feature of the topology of *m*_62_, overcoming the competitive exclusion principle.

When designing new systems, minimising the number of genetic parts will reduce the number of experimental variables, improving the ease of construction and optimisation of a system. We subset the model space by the number of expressed parts in the system (maximum two QS and two bacteriocin), yielding subsets containing candidate models with two, three and four expressed parts (low complexity to high complexity). We identify the candidates with the highest posterior probability in each subset (Fig. 2b). The posterior probability increases despite the larger parameter spaces, which is important because ABC SMC will naturally favour models which yield stable steady state with the smallest possible number of parameters (Occam’s razor)^36^. We see that all three models have SL motifs, where a strain is sensitive to the bacteriocin it produces. All three models are devoid of other-limiting (OL) motifs, where a strain is sensitive to a bacteriocin produced by another strain.

The Bayes factor (BF) is a ratio between the marginal likelihoods of two models, giving a quantification of support for one model compared with another. BF > 3.0 indicates evidence of a notable difference between the two models, while BF < 3.0 suggests insubstantial evidence^37^ (Table 2). The BF of *m*_66_ compared with *m*_48_ suggests substantial improvement in the posterior probability can be made by increasing complexity. However, the BF of *m*_48_ compared with *m*_62_ suggests insubstantial evidence behind this improvement in posterior probability (Fig. 2b). These diminishing returns when increasing system complexity hold important ramifications for system design. The introduction of an additional QS part to move from *m*_48_ to *m*_62_ may not be worthwhile for the minor improvement in steady state robustness.

**Table 2:** Bayes factor categorisation to describe evidence in favour of *m*_1_, compared with *m*_2_.

Bayes factor (BF) value	Evidence against *m*_2_ (in favour of *m*_1_)
1–3	Very weak
3–20	Positive
20–150	Strong
>150	Very strong

Model selection has identified the best performing designs for producing stable communities. However, the parts used in the design may require specific characteristics or chemostat settings. ABC SMC also produces posterior parameter distributions for each model, giving us information about the parameter values necessary to yield stable steady state. Figure 2c shows the posterior distributions of several tunable parameters in *m*_66_ and *m*_62_. The dilution rate of the chemostat (*D*) is a directly tunable parameter and the maximal expression rate of the bacteriocin ($K{B}_{\max }$) can be tuned through choice of promoter and ribosome-binding site^38^. The growth rates (${\mu }_{\max }$) can be tuned through choice of base strains or auxotrophic dependencies^39,40^.

For *m*_66_, the correlation coefficients between strain maximal growth rates (*μ*_max1_ and *μ*_max2_) shows the parameters are loosely correlated. Additionally, we see that *N*_1_ requires a higher maximal growth rate (*μ*_max1_) than that of *N*_2_ (*μ*_max2_). The faster maximal growth rate of *N*_1_ is necessary to counteract self-limitation that is negatively regulated by the population of *N*_2_. Conversely, *m*_62_ shows a wider distribution of strain growth rates at stable steady states and a low correlation coefficient. This indicates that this topology does not heavily depend on specific growth rates or related growth rates between the two strains in order to produce a stable steady state. $K{B}_{\max }$ for all bacteriocins is tightly constrained to high maximal bacteriocin expression rates. The distributions of *D* in both systems show a lower dilution rate is important for stable steady state. The steady state compositions for *m*_66_ frequently contain *N*_1_ in high proportion compared with *N*_2_, whereas *m*_62_ will commonly yield compositions with more even representation of *N*_1_ and *N*_2_ at steady state (Supplementary Fig. 2).

### Self-limiting motifs stabilise two strain systems

The dendrogram of Fig. 2a highlights a cluster of high performing models that are closely related. This suggests underlying interactions of the model space exist that are important for producing communities with stable steady state.

**Fig. 3:**
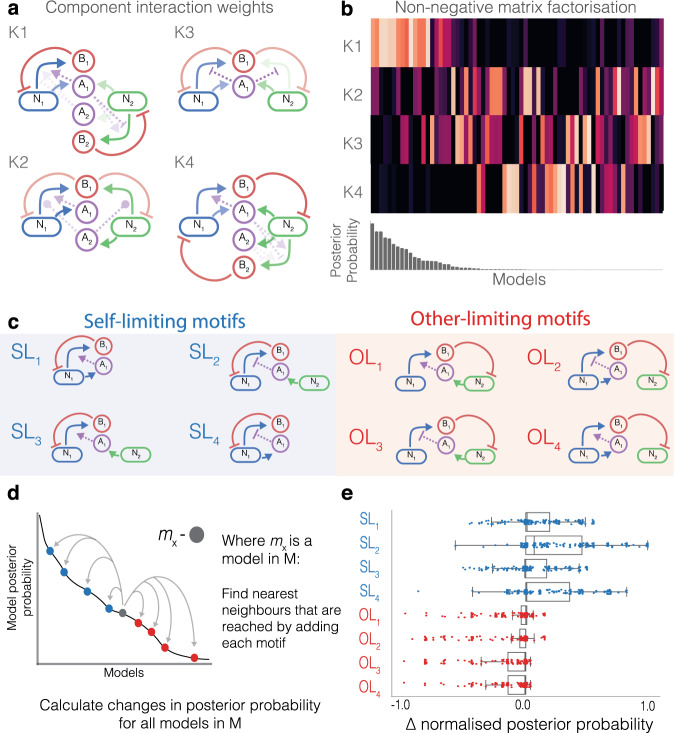
Contribution of network motifs to stability. **a**, **b** Non-negative matrix factorisation (NMF) analysis to learn motifs in model space using four components (*K* = 4). **a** Four components learned by NMF, the line opacity indicates the coefficient of the interaction. **b** Visualisation of the component weights for each model. Each column is a model, with light colours corresponding to high weight and dark colours low weight. **c**–**e** Manually curated minimal motifs capture interaction importance. **c** Minimal motifs split into self-limiting (SL) and other-limiting (OL) by the direction of bacteriocin killing. **d** Illustration of the algorithm used to generate each datapoint in **e**. Moving from a model to the nearest neighbours that can be built by adding a motif will produce a change in model posterior probability. **e** Boxplots and scatter plot showing the change in posterior probability when adding each motif to a model. The boxplots show the median, first and third quartile. The lower and upper whiskers mark the 5th and 95th percentiles, respectively. *n* = 224 for each motif.

Non-negative matrix factorisation (NMF) is an unsupervised machine learning method we can use to reduce the dimensionality of the interaction space^41^. We can use NMF to help us understand the underlying motifs and how they affect community stability. We represent each model by the interactions present in the system (Fig. 2a). NMF takes these interactions and learns a number of clusters (*K*), models can be rebuilt by a weighted sum of these clusters. In our case, these clusters can be represented as interaction motifs. We set *K* = 4, in order to give us a digestible summary of the model space. Figure 3a shows the learned motifs that can be used to represent the entire model space. Figure 3b shows the component weights for each model, defining the membership each model has for each motif. The models are shown in descending order of posterior probability, we can see that *K*1 is heavily weighted in the top performing models. The motif *K*1 refers to SL only interactions where the strain is sensitive to the bacteriocin it produces (Fig. 3a, b). The top models are consistently assigned low weights for *K*4 (Fig. 3a, b), a motif which refers to OL only interactions, where the strain is sensitive to a bacteriocin produced by the other strain (Fig. 3a).

We use the indications produced by NMF to curate our own discrete motifs, improving the ease of interpretation. *K*1 and *K*4 show us the direction of bacteriocin sensitivity is an important feature and we proceed to investigate this further. All models can be built by combining eight fundamental motifs which can be categorised as either SL or OL, based on the direction of bacteriocin sensitivity (Fig. 3c). Within each category, motifs are differentiated by the mode of bacteriocin regulation (Fig. 3c). For example, *m*_66_ = SL_2_, *m*_48_ = SL_4_ + SL_2_ and *m*_62_ = SL_2_ + SL_2_. In order to assess the importance of each motif for producing stable communities we perform a motif impact analysis. For each model we identify the nearest neighbours in the model space that can be built by adding each motif and calculate the change in posterior probability for each neighbour (Fig. 3d). By repeating this across the entire model space, we are able to quantify whether a motif is stabilising or destabilising (Fig. 3e). The lower quartiles of SL motifs all show lower negative change magnitudes compared with the lower quartiles of OL motifs. The upper quartiles of SL motifs show a higher positive change magnitude than that of OL motifs. Together these show the addition of SL motifs more often result in an improved posterior probability, whereas addition of OL motifs more often result in decreased posterior probability. The upper quartile of SL_2_ shows the motif has the most stabilising effect, closely followed by SL_4_. We see these findings are reflected by top models identified in Fig. 2b, where all models are constructed with SL_2_ and SL_4_ motifs.

The total output of bacteriocin by a population is a function of the population’s density. All SL motifs therefore possess a fundamental negative feedback relationship between growth rate and density, augmented by the mode of QS regulation. Conversely, the population density and growth rate of a strain in OL motifs are decoupled. This lack of feedback is a clear explanation as to why we see SL motifs as positive contributors to stability while OL motifs have a destabilising effect. By comparing the posterior probabilities of *m*_62_ and Supplementary Fig. 4, we show that while self-limitation interactions are important for viability, interdependence between the strains is necessary to produce the most robust design (Supplementary Fig. 3).

### Designing three strain communities that achieve steady state

**Fig. 4:**
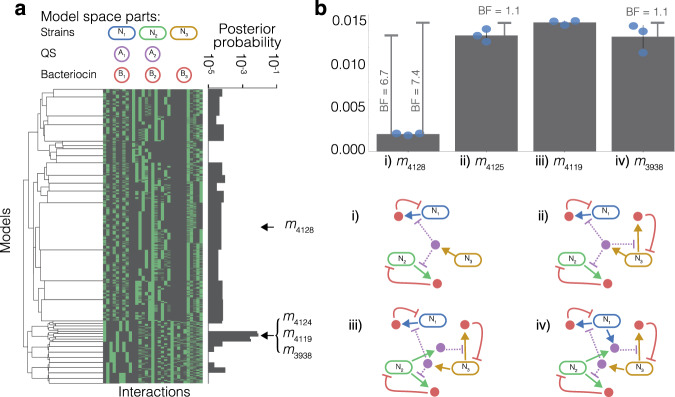
Output of AutoCD for three-strain stable steady state objective. **a** Dendrogram is generated by hierarchical clustering of the adjacency matrices for each model in the three-strain model space. We set the limit of number of levels to 5, in order to show high level groups. Each column of the heatmap represents a possible interaction between state variables, where green indicates the interaction exists for the model and black indicates absence of the interaction. The posterior probability plot shows the average posterior probability within each group of models. **b** Shows the models with highest posterior probability when subsetted for number of parts expressed, in order of increasing complexity (3, 4, 5 and 6 expressed parts). The bar chart shows the mean model posterior probability across three experiments, represented by the scatter points, the error bars indicate the standard deviation. *n* = 3, where each replicate consists of 825,000 accepted particles. Bayes factors (BF) are shown for pairwise comparison of the three models and error bars show the standard deviation between three repeat experiments. Model schematics show the interactions between strains (blue, green and red), bacteriocin (red) and QS molecules (purple). Models with two parts showed posterior probability 0.0 and are not shown.

While several studies have demonstrated the ability to establish synthetic two-strain systems^19,22,42–51^, efforts with three strains are sparser^23,52,53^. Having demonstrated the automated design of two-strain systems, we next tackle the far larger challenge of designing stable three-strain communities. The addition of a single strain significantly increases the parameter space, engineering options and possible interactions. We define our available parts consisting of three strains (*N*_1_, *N*_2_, *N*_3_), three bacteriocins (*B*_1_, *B*_2_, *B*_3_) and two orthogonal QS systems (*A*_1_, *A*_2_). We maintain the same strain engineering restrictions, allowing up to one QS expression and up to one bacteriocin expression per strain. Each strain can be sensitive to up to one bacteriocin. Given the available parts and engineering limits, the model space generator yields 4182 unique models (see Supplementary Fig. 5 for visualisation of each candidate model). Due to the much greater number of models, we group models based upon the interactions in each model by hierarchical clustering for up to five levels. The average posterior probabilities of each cluster are shown (Fig. 4a). 3289 models have a posterior probability of zero, highlighting how much more difficult this design scenario is. ABC SMC identifies *m*_4119_ as the system with the highest posterior probability for producing stable steady state. *m*_4119_ consists of two QS molecules; *A*_1_ is produced by *N*_2_, *A*_2_ is produced by *N*_3_ (Fig. 4b). The QS molecules repress the expression of SL bacteriocins produced by each population. Using the minimal motifs defined in Fig. 3c, *m*_4119_ can be summarised as *m*_4119_ = 3 × SL_2_. We group the model space on the counts of heterologous expression in the system, yielding subsets containing candidate models with three, four, five and six expressed parts (Fig. 4b). Models with two heterologously expressed parts all had a posterior probability of 0.0 and are not shown. Again, we see a diminishing increase in posterior probability that comes with increasing complexity. *m*_3938_ is the more complicated neighbour of *m*_4119_, where *N*_1_ is also contributing with production of *A*_1_, resulting in a fall in the posterior probability. The increase in posterior probability that occurs when moving from *m*_4125_ to *m*_4119_ has BF < 3.0, indicating the difference between the posterior probability of the two models is not substantial. These system comparisons highlight the trade-off between increasing complexity and improving system performance. In a similar fashion to the two-strain model space, the top performing models are dominated by SL only interactions (Supplementary Fig. 1).

### Multiple engineered bacteriocins are more important than multiple orthogonal QS systems

Our results have identified top performing models in the two-strain and three-strain model spaces. We have also highlighted the diminishing returns that occur with increasing model complexity in top performing models. Next we aim to summarise the importance of different parts and their contribution to the stable steady state objective behaviour, further enabling us to triage genetic parts for construction in the lab.

**Fig. 5:**
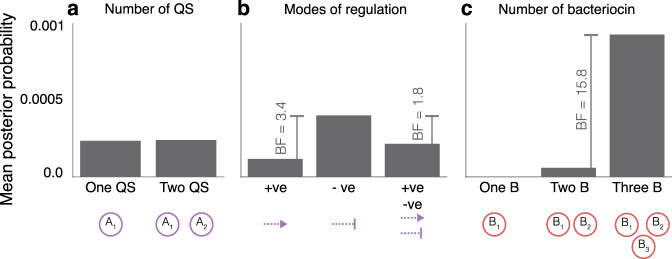
Average posterior probabilities associated with the number of genetic parts. **a** Comparing systems with one and two orthogonal QS parts. **b** Comparing modes of bacteriocin regulation in the system by subsetting for systems with induction (+ve), repression (−ve) or both (+ve, −ve). **c** Comparing systems with one, two and three bacteriocin.

Figure 5 shows a summary of the parts used to construct three-strain systems and the average posterior probabilities they yield. This gives us important information to form heuristic rules in the design of three-strain systems. Figure 5a shows a very similar posterior probability when comparing two QS systems rather than one. Figure 5b demonstrates the substantial advantage of repressive QS regulation of bacteriocin production over inducible systems. Figure 5c shows very strong evidence in favour of using three bacteriocins to produce stable steady state in three-strain systems. These three statistics suggest that on average there is little advantage to be gained in the use of two QS systems, and priority should be given to the use of a single repressive QS to regulate three bacteriocin systems, such as we see in *m*_4125_.

### Defining stable steady state population ratios in three-strain systems

Natural microbial communities are observed to contain species in abundances differing over orders of magnitude^54,55^. Together the individual species can contribute to an aggregate community function^56,57^. Synthetic communities can take advantage of aggregate community output by their application to improving yields and efficiency of bioproduction pathways via the distribution of genetic processes between subpopulations^43,50^. Biosynthesis studies using cocultures have shown the importance of optimising inoculation ratios to maximise community outputs^58,59^. Therefore being able to define the steady state composition of a synthetic community is a valuable feature. Here we demonstrate that a form of post-processing can be applied to the output of ABC SMC by applying a secondary threshold, identifying key parameters that enable fine tuning of stable steady state population densities.

**Fig. 6:**
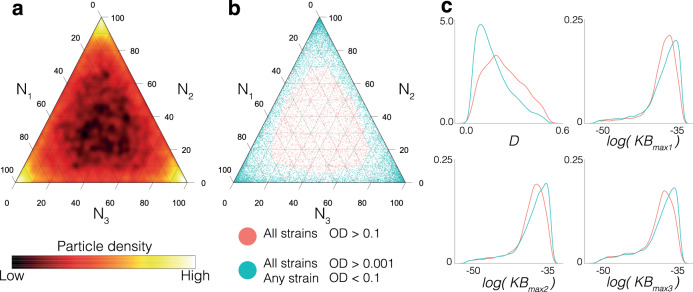
Distribution of population densities in model 4119. Axes of ternary diagrams (**a** and **b**) show percentage composition of the community. **a** Heat map showing the community composition at stable steady state in model 4119. Axes refer to percentage representation for each strain in the community. **b** Scatter plot of the stable steady state systems, highlighting the secondary threshold where all strains have OD > 0.1 (red), and the primary objective only where any strain has 0.001 < OD < 0.1. **c** Density plots comparing the parameter distributions of four parameters that show the greatest divergence to produce the secondary threshold: Dilution rate (*D*) and maximal bacteriocin expression rates (*KB*_max1_, *KB*_max2_, *KB*_max3_). The Kolmogorov–Smirnov values for the two objectives are 0.25, 0.12, 0.13 and 0.12, respectively.

The ${\epsilon }_{{F}_{3}}$ threshold value ensures all simulations in the final population have an OD > 0.001. Figure 6a shows the community composition distribution of *m*_4119_. The majority of accepted particles show a final community composition that is dominated by a single strain. Using the final population distances from ABC SMC we can apply a secondary threshold and identify how the system can be tuned to produce a more evenly distributed community composition. We set a secondary threshold, stipulating that all strains must be of OD > 0.1 (pink) (Fig. 6b). Therefore strains that do not meet the secondary threshold have 0.001 < OD < 0.1 (blue) (Fig. 6b). From these two subsets we generate separate parameter distributions and calculate the divergence using Kolmogorov–Smirnov (KS). Parameter distributions that show the greatest divergence are important for changing the system behaviour from one that is dominated by a single strain, to one that has a more even distribution of strain densities. The distributions of four parameters that exhibit greatest divergence are shown in Fig. 6c. A higher dilution rate (*D*) and lower maximal bacteriocin expression rates (${K}_{{B}_{\max }1}$, ${K}_{{B}_{\max }2}$, ${K}_{{B}_{\max }3}$) are associated with producing a more evenly distributed community composition. Importantly, all three parameters are realistically tunable. The dilution rate can be controlled directly through the chemostat device, while bacteriocin expression rates can be changed through the choice of promoters and ribosome-binding sites.

## Discussion

Synthetic communities built to date have employed the use of QS, metabolic dependencies, intracellular lysis proteins, toxins and extracellular AMPs to engineer interactions that enable community formation^23,51,52^. When designing a synthetic community, the fundamental interactions in the system itself is often directed by mimicking ecological interactions found in nature, or by rational judgement. As the possible types of engineered interaction increases, so does the need for comprehensive assessment of the vast model spaces. The modelling and statistical framework demonstrated here addresses this design problem. With our examples we have highlighted important design features and heuristic rules for building synthetic steady state communities. As we move to increasingly complex multi-strain systems, bottom-up approaches have shown that understanding pair-wise interactions can be used to build up to larger stable communities^60^.

We have identified optimal system designs using bacteriocins and QS for stable steady state in two-strain and three-strain communities. *m*_62_, the top model of the two-strain model space uses a cross-protection mutualism, whereby the density of each subpopulation inhibits the self-limitation of the other. Similarly, in the three-strain model space *m*_4119_ has pairwise cross-protection mutualism between two subpopulations and a dependent subpopulation (Fig. 4b). Cross-protection mutualism has previously been incorporated in synthetic microbial communities via the mutual degradation of externally supplied antibiotics^46^. Metabolic interdependencies can also be employed to engineer mutualism^47,48^. All top performing models used SL interactions to produce stable steady state dynamics. Self-limitation is observed in many natural biological communities, normally in a response to stress^61,62^. These processes, while detrimental to the individual, provide a net benefit to the community through release of a public good—they are altruistic processes^63^. Altruistic cell death is conserved throughout different species implying a competitive advantage in natural environments^64^. SL interactions have previously been used to overcome competitive exclusion by employing lysis proteins regulated by QS in a two-strain culture^51^. The inducible expression of SL bacteriocins under tightly controlled promoters has also been demonstrated^65^. Additionally, in our recent work we have demonstrated the use of bacteriocins to stabilise communities^66^. Random sampling or encapsulation of microbial networks has been demonstrated experimentally in both ecological and synthetic contexts^67,68^. These high throughput approaches could be used to validate our findings, combining differentially engineered strains with one another to give a view of strain combinations that form stable communities.

The robustness of SL interactions can be explained by the feedback loops involved. Total bacteriocin output by a subpopulation is heavily dependent upon its population density; low population density will naturally have a low output of bacteriocin^69^, making QS a secondary level of regulation. This is supported by both two-strain and three-strain scenarios where we observe the diminishing returns that come with increasing complexity. Figure 5 shows that increasing the number of bacteriocins in a system yields greater increases in stability than increasing the number of QS systems. A closed feedback loop exists between the bacteriocin expression rate and the population density, an important reason why we see all SL motifs generally show positive contribution to stability. Conversely, in OL motifs the population expressing the bacteriocin will not be negatively affected and therefore a closed feedback loop does not exist.

Ecological studies using generalised Lotka–Volterra approaches frequently show that negative, intraspecific interactions are of central importance to the stability of ecological networks^70–72^. In our models, SL interactions, dilution rate and limited nutrients are all analogous to negative, intraspecific, density-dependent interactions described at a more detailed level; particularly regarding time delays and accumulation of bacteriocin or QS molecules that may occur. Our results align with previous findings and provide insight into the relative importance of different types of interactions in a synthetic biology context. Additionally, studies have previously shown that higher connectance in mutualistic ecological networks promotes persistence and resilience^73^. All our top performing models contain forms of mutualism; in these models we also see a trend of increasing robustness with complexity which is analagous to connectance (Figs. 2b and 4b).

Studies have traditionally used eigenvalue analysis to investigate the stability properties of random interaction ecological networks^71,73,74^. Similar approaches could be applied to the synthetic community model spaces shown here. The Bayesian approach and time series analysis used here allows us to select for defined temporal characteristics of transient behaviour that represent a definition of a stable system that is achievable experimentally. In principle, eigenvalues could also be included within a distance measure of asymptotic local stability. However, we found they did not improve the classification of behaviour in these models. Finally, we showed that the posterior parameter distribution from ABC SMC can be used to make decisions on part characteristics and experimental conditions (Figs. 2c and 6c). Our results show the dilution rate (*D*) is an important experimental parameter for producing stable steady state, and tuning the community composition. The rate of removal of molecules from the environment can produce very different population dynamics. This is supported by previous work where the dilution rate has been demonstrated to be important for determining the population dynamics^6,22,46^. We also show our methodology can identify systems that are robust to differences in growth rate, highlighted by the comparison of *m*_66_ and *m*_62_ in Fig. 2c. Together these draw attention to important part characteristics that should be considered when constructing a stable community. It should be emphasised that while the design rules we have identified hold true for a stable steady state objective, it may not be the case for other objective population dynamics, such as oscillations. New objectives can be investigated by changing the distance functions which describe the population dynamics.

The framework we have developed offers a natural entry point to the design-build-test cycle, providing a data informed roadmap for building a robust synthetic community with a desired behaviour. We have revealed stable steady state systems in a two-strain and three-strain model space, and generated impactful rules and heuristics for their construction. The flexibility of this framework enables us to quickly redefine population level behaviours depending on the required application.

## Methods

### Model space generator

Models are generated from a set of parts, which are expressed by different strains in the system. We represent an expression configuration through a set of options. We define the options for expression of *A* in each strain, where the options are not expressed, expression of *A*_1_, and expression of *A*_2_ (0, 1 and 2). We define the options for expression of bacteriocin, which for the two-strain model space includes no expression, expression of *B*_1_ or expression of *B*_2_ (0, 1, and 2). For the three-strain model space, this includes includes no expression, expression of *B*_1_, expression of *B*_2_ or expression of *B*_3_ (0, 1, 2 and 3, respectively). Lastly we define the mode of regulation, *R*, for the bacteriocin, which can be either induced or repressed (0 and 1). This is redundant if a bacteriocin is not expressed.

Two strain:

$$A 	=\{0,1,2\}\\ B 	=\{0,1,2\}\\ R 	=\{0,1\}$$

Three strain:

$$A 	=\{0,1,2\}\\ B 	=\{0,1,2,3\}\\ R 	=\{0,1\}$$

This enables us to build possible part combinations that can be expressed by a population. Let *P*_C_ be a family of sets, where each set is a unique combination of parts:

$${P}_{{\mathrm{{C}}}}=A\times B\times R$$

Each strain in a system can be sensitive to up to one bacteriocin. Let *I* represent the options for strain sensitivity. In the two-strain model space, the options are insensitive, sensitive to *B*_1_ or sensitive to *B*_2_ (0, 1 and 2, respectively). In the three-strain model space, the options are insensitive, sensitive to *B*_1_, sensitive to *B*_2_ or sensitive to *B*_3_ (0, 1, 2 and 3, respectively).

Two strain:

$$I=\{0,1,2\}$$

Three strain:

$$I=\{0,1,2,3\}$$

Each strain is defined by its sensitivities and expression of parts. Let *P*_E_ be all unique engineered strains:

$${P}_{{\mathrm{{E}}}}=I\times {P}_{{\mathrm{{C}}}}$$

which can be combined to form a model yielding unique combinations in two strains and three strains:

Two strain:

$${P}_{{\mathrm{{M}}}}={P}_{{\mathrm{{E}}}}\times {P}_{{\mathrm{{E}}}}$$

Three strain:

$${P}_{{\mathrm{{M}}}}={P}_{{\mathrm{{E}}}}\times {P}_{{\mathrm{{E}}}}\times {P}_{{\mathrm{{E}}}}$$

Finally, we use a series of rules to remove redundant models. A system is removed if:

1. Two or more strains are identical, concerning bacteriocin sensitivity and combination of expressed parts.
2. The QS regulating a bacteriocin is not expressed by a strain.
3. A strain is sensitive to a bacteriocin that is not expressed by a strain.
4. A bacteriocin is expressed that no strain is sensitive to.

This cleanup yields the options which are used to generate ODE equations for system.

### System equations

State variables in each system are rescaled to improve speed of obtaining numerical approximations:

3 $${N}_{x}^{\prime}={N}_{x}{C}_{N}$$

4 $${B}_{z}^{\prime}={B}_{z}{C}_{B}$$

5 $${A}_{y}^{\prime}={A}_{y}{C}_{A}$$

Each model is represented as sets defining the system:

6 $${\mathbb{N}}=\{1,2...x\}$$

7 $${\mathbb{B}}=\{1,2...z\}$$

8 $${\mathbb{A}}=\{1,2...y\}$$

The system is represented as differential equations:

9 $$ \frac{{\mathrm{{d}}}{N}_{x}}{{\mathrm{{d}}}t}={N}_{x}{\mu }_{x}(S)-{N}_{x}\mathop{\sum }\limits_{z=1}^{{\mathbb{B}}}\omega ({B}_{z}^{\prime})-{N}_{x}D$$

10 $$\frac{{\mathrm{{d}}}S}{{\mathrm{{d}}}t}=D({S}_{0}-S)-\mathop{\sum }\limits_{x=1}^{{\mathbb{N}}}\frac{{\mu }_{x}{N}_{x}^{\prime}}{{\gamma }_{x}}$$

11 $$\frac{{\mathrm{{d}}}{B}_{z}}{{\mathrm{{d}}}t}=\mathop{\sum }\limits_{x=1}^{{\mathbb{N}}}\frac{({k}_{{B}_{x,z}}{N}_{x}^{\prime})}{{C}_{B}}-D{B}_{z}\quad \,\,\,$$

12 $$\frac{{\mathrm{{d}}}{A}_{y}}{{\mathrm{{d}}}t}=\mathop{\sum }\limits_{x=1}^{{\mathbb{N}}}\frac{{k}_{{A}_{x,y}}{N}_{x}^{\prime}}{{C}_{A}}-D{A}_{y}\quad \, \quad$$

Growth is modelled by Monod’s equation for nutrient limited growth:

13 $${\mu }_{x}(S)=\frac{{\mu }_{{x}_{\max }}S}{{K}_{X}+S}$$

Killing by bacteriocin is modelled via a Hill function, where ${\omega }_{\max }=0$ if strain is insensitive:

14 $$\omega ({B}_{z}^{\prime})={\omega }_{\max }\frac{{B}_{z}^{^{\prime} {n}_{\omega }}}{{K}_{\omega }^{{n}_{\omega }}+{B}_{z}^{^{\prime} {n}_{\omega }}}$$

Induction or repression of bacteriocin expression by QS, *A*_*y*_:

15 $${k}_{{\mathrm{{B}}}}(z,y)=K{B}_{\max }z\frac{{A}_{y}^{^{\prime} {n}_{z}}}{{K}_{{{\mathrm{{B}}}}_{z}}^{{n}_{z}}+{A}_{y}^{^{\prime} {n}_{z}}}$$

16 $${k}_{{\mathrm{{B}}}}(z,y)=K{B}_{\max }z\frac{{K}_{{B}_{z}}^{{n}_{z}}}{{K}_{{B}_{z}}^{{n}_{z}}+{A}_{y}^{^{\prime} {n}_{z}}}$$

Simulations were conducted for 1000 h, the final 100 h were used to calculate the summary statistics and were stopped early if the population of any strain fell below 1e−10 (extinction event). Simulations with an extinction event have distances set to maximum in order to prevent excessive time spent simulating collapsed populations.

### Bayesian inference

Let *θ* ∈ Θ be a sampled parameter vector with a prior *π*(*θ*). Given an objective of *x*_0_, where *x*_0_ exists in the solution space, ${x}_{0}\in {\mathcal{D}}$. We define the likelihood function for the objective behaviour as *f*(*x*_0_∣*θ*). Bayes’ theorem gives us the posterior distribution of *θ* that exists for the objective *x*_0_:

17 $$\pi (\theta | {x}_{0})=\frac{f({x}_{0}| \theta )\pi (\theta )}{\pi ({x}_{0})}$$

We can rewrite *π*(*x*_0_) where *a* and *b* represent the lower and upper bounds of the parameter value:

18 $$\pi ({x}_{0})=\mathop{\int}\nolimits_{a}^{b}f({x}_{0},\theta ){\mathrm{{d}}}\theta =\mathop{\int}\nolimits_{a}^{b}f({x}_{0}| \theta )\pi (\theta ){\mathrm{{d}}}\theta$$

The posterior distribution informs us of the parameter distribution that gives rise to the objective:

19 $$\pi (\theta | {x}_{0})=\frac{f({x}_{0}| \theta )\pi (\theta )}{\mathop{\int}\nolimits_{a}^{b}f({x}_{0}| \theta )\pi (\theta ){\mathrm{{d}}}\theta }$$

Let *m* be a model from a vector of competing models, *M*, such that *m* ∈ *M* = {*m*_0_,*m*_2_,...*,m*_*n*_}. Each model has its own parameter space, allowing us to define a joint space, (*m*, *θ*) ∈ *M* × Θ.

We can write Bayes’ theorem in the context of a model space:

20 $$\pi (m| {x}_{0})=\frac{f({x}_{0}| m)\pi (m)}{{\int}_{M} \,\, f({x}_{0}| m^{\prime} )\pi (m^{\prime} ){\mathrm{{d}}}m^{\prime} }$$

Since the *M* is discrete, we can rewrite this as:

21 $$\pi (m| {x}_{0})=\frac{f({x}_{0}| m)\pi (m)}{{\sum }_{M}\, \, f({x}_{0}| m^{\prime} )\pi (m^{\prime} )}$$

The marginal likelihood of the model, *f*(*x*_0_∣*m*), is the expectation of the likelihood function taken over the model parameter prior distribution. It measures a model’s fit:

22 $$f({x}_{0}| m)={\int}_{{{{\Theta }}}_{M}}\pi (\theta | m)f({x}_{0}| \theta ,m){\mathrm{{d}}}\theta$$

### Approximate Bayesian computation

Writing the likelihood function, *f* (*x*_0_∣*θ*), in terms of summary statistics can be difficult. We bypass this and approximate the posterior by generating data from a model. We can sample a parameter vector from the prior, θ^*^ ~ *π*(*θ*), which is simulated to yield a data vector, x^*^. This can be written as a conditional, x^*^ ~ *f* (*x*∣θ^*^), which also gives the joint density, *π*(*θ*,*x*). In order to obtain the posterior distribution that satisfies our objective behaviour, *x*_0_, we apply a conditional to define whether a generated data vector, x^*^, belongs to the objective *x*_0_.

If *x* = *x*_0_

23 $$\pi (\theta | x,{x}_{0})=\frac{\pi (\theta )f(x| \theta )}{\pi (\theta )f(x| \theta ){\mathrm{{d}}}x{\mathrm{{d}}}\theta }$$

Else

24 $$\pi (\theta | x,{x}_{0})=0$$

Let *ρ*(*x*, *x*_0_) be a distance function that compares a simulation to the objective. Using distance threshold, *ϵ*, we can define values below which the distance is acceptably small. We can redefine *π*(*θ*∣*x*, *x*_0_) in the context of thresholds to obtain an approximation of the posterior.

If *ρ*(*x*, *x*_0_) < *ϵ*

25 $${\pi }_{\epsilon }(\theta | x,{x}_{0})=\frac{\pi (\theta )f(x| \theta )}{\pi (\theta )f(x| \theta ){\mathrm{{d}}}x{\mathrm{{d}}}\theta }$$

Else

26 $${\pi }_{\epsilon }(\theta | x,{x}_{0})=0$$

The smaller *ϵ* is and the larger the number of simulations conducted, the more accurate the representation of the true posterior will be. We can write this marginal posterior distribution as:

27 $$\pi ({\theta }^{* }| \rho ({x}^{* },{x}_{0}))\le \epsilon \approx \pi (\theta | {x}_{0})$$

### Model selection with ABC SMC

 In this paper, we use a variant of ABC, ABC Sequential Monte Carlo (ABC SMC)^36^. Particles are sampled from the prior distributions. Each particle represents a sampled model and sampled parameters for that model. ABC SMC evolves particles sampled from the prior distribution through a series of intermediate distributions and perturbations. Importance weighting is used to define their sample probability for the next distribution. The distance threshold, *ϵ*, is decreased between distributions, moving the acceptance criteria closer to the objective. These features aim to improve the acceptance rate of particles while maintaining a good approximation of the posterior distribution (see Supplementary Algorithm 1 for more details).

### Bayes factor

The BF can be used to help us interpret how much better (or worse) one model is than the other. Given two models, *m*_1_ and *m*_2_, the BF is calculated as

28 $${\mathrm{{BF}}}=\frac{P({m}_{1}| x)/P({m}_{2}| x)}{P({m}_{1})/P({m}_{2})}$$

*P*(*m*_*i*_) is the prior, and *P*(*m*_*i*_∣*x*) is the posterior probability. Given uniform priors, *P*(*m*_*i*_) = 1/*M*, where *M* is the number of models. Therefore we can simplify to:

29 $${\mathrm{{BF}}}=\frac{P({m}_{1}| x)}{P({m}_{2}| x)}$$

The BF is a measure of the support for *m*_1_ relative to *m*_2_. It accounts for the number of parameters, or complexity of the two models. The BF allows us to directly compare the weight of evidence for and against the two models and has the advantage that it can be used to compare non-nested models. Two BFs can be compared directly, since they both represent evidence in favour of the hypothesis^36,37^. We therefore use BFs to directly compare the ability of two models to represent the objective population behaviour. Table 2 allows us to interpret BF.

### Software packages and simulation settings

ABC SMC model selection algorithm was written in python using Numpy^75^, Pandas and Scipy^76^. ODE simulations were conducted in C++ with a Rosenbrock 4 stepper from the Boost library^77^. All simulations use an absolute error tolerance of 1e−9, and relative error tolerance of 1e−4. NMF was conducted using Scikit-learn^78^. Dendrograms were made from SciPy, using the unweighted pair group method with arithmetic mean (UPGMA) clustering algorithm^76^. Ternary diagrams were made using python package python-ternary^79^. Parameter distribution plots were made in R using ggplot2^80^.

### Reporting summary

Further information on research design is available in the Nature Research Reporting Summary linked to this article.

## Supplementary information

Supplementary Information

Reporting Summary

## Data Availability

The data generated and used to create figures can be found at 10.5281/zenodo.4286040. Any other relevant data can be obtained from the authors upon reasonable request.

## Code availability

AutoCD code repository can be found at https://github.com/ucl-cssb/AutoCD/^88^. The repository includes configuration files for the two- and three- strain experiments conducted in this study. All code to recreate figures can be found at 10.5281/zenodo.4286040.
