# Supplementary material for: Automated design of synthetic microbial communities
Source: Nat Commun. 2021 Jan 28;12:672. doi: 10.1038/s41467-020-20756-2 (PMC7844305; doi:10.1038/s41467-020-20756-2)

Supplementary information: Automated design of synthetic microbial communities

Behzad D. Karkaria,[1] Alex J. H. Fedorec,[1] and Chris P. Barnes[1,2*]

[1]Department of Cell & Developmental Biology, University College London, London, WC1E 6BT, UK

[2]UCL Genetics Institute, University College London, London, WC1E 6BT, UK

*To whom correspondence should be addressed;

E-mail: christopher.barnes@ucl.ac.uk

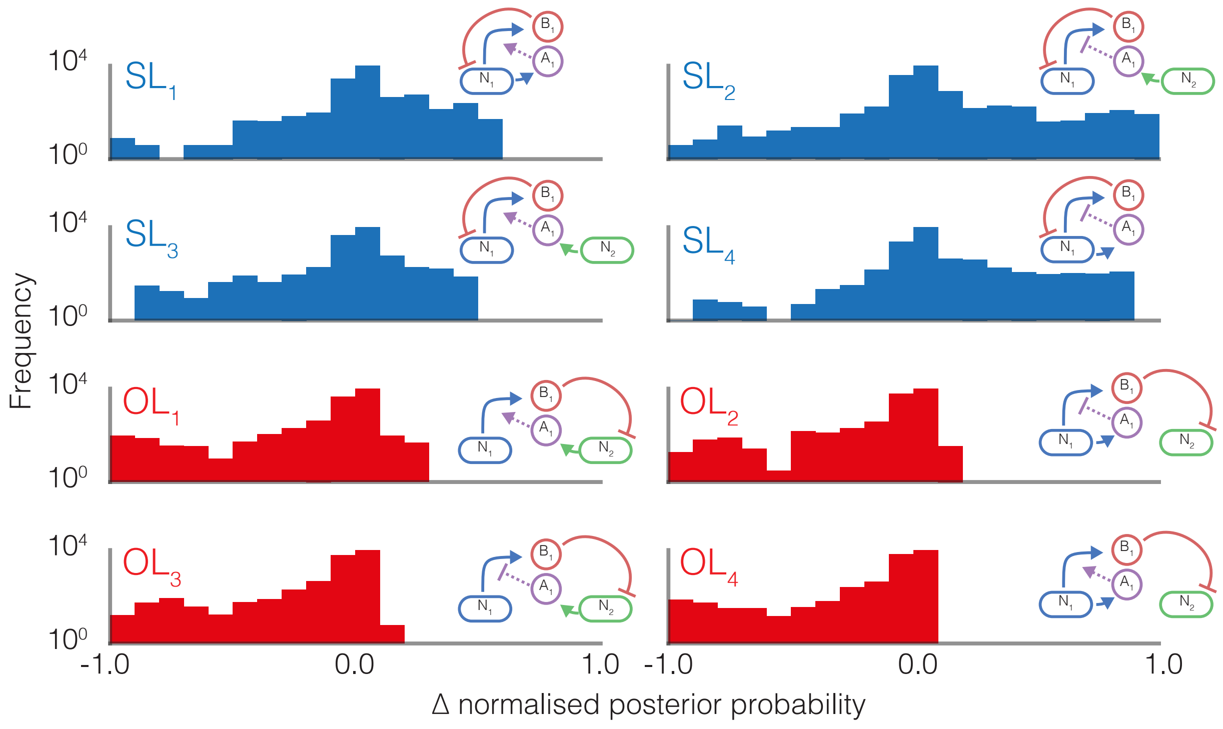

Supplementary Figure 1: Motif impact analysis in three strain model space. Histograms show the change in posterior probability when adding each motif to a model in the three strain model space.

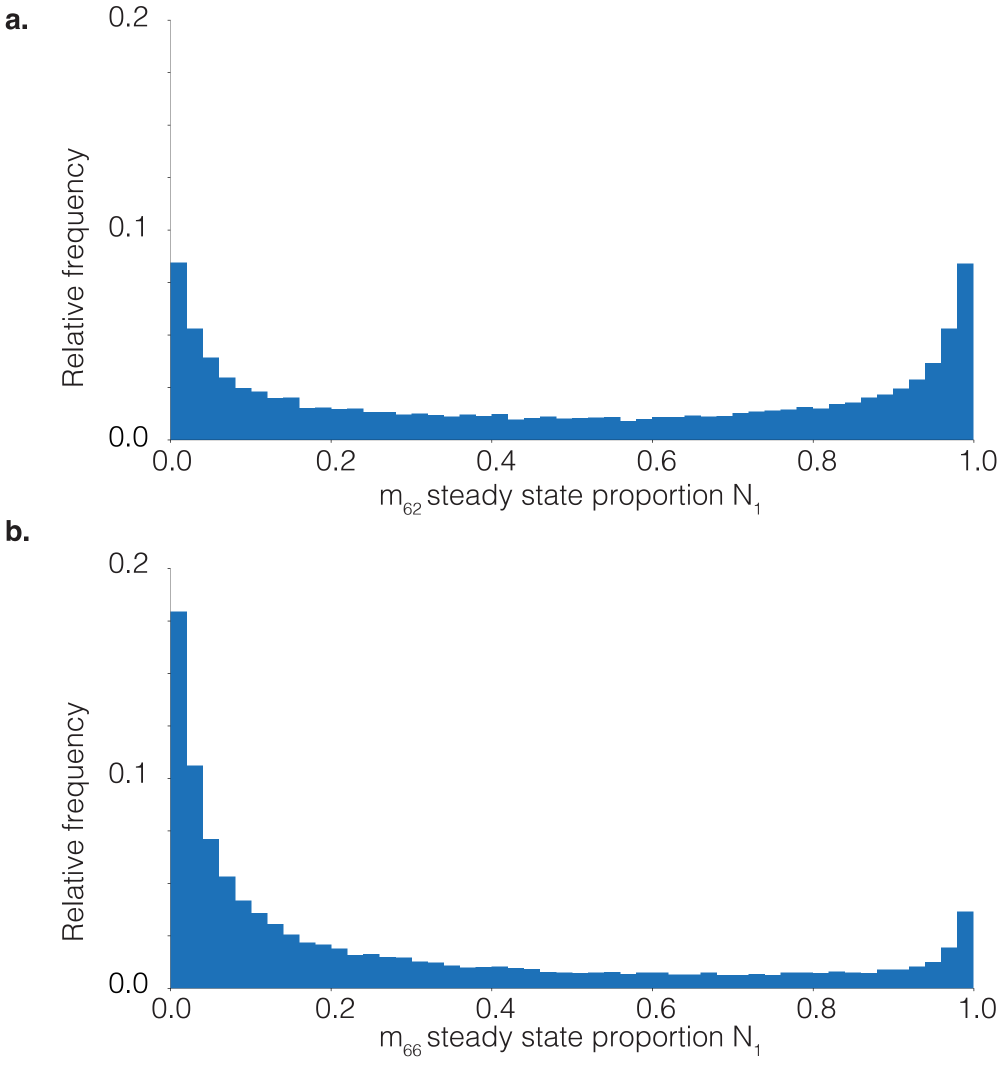

Supplementary Figure 2: Stable steady state population distribution in two strain models. **a** A histogram of the proportion of population occupied by strain N_1 at stable steady for m_{62}. **b** A histogram of the proportion of population occupied by strain N_1 at stable steady for m_{66}.

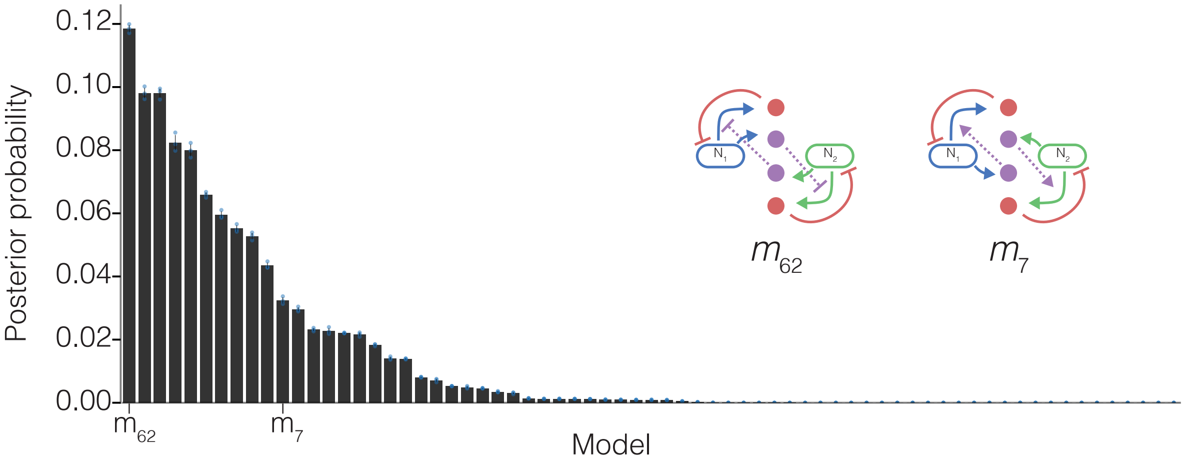

Supplementary Figure 3: Posterior probability of two strain model space in order of descending posterior probability. m_{62} and m_7 are highlighted, drawing attention to two models with and without interdependence between strains. The configuration of these parts is what differentiates the robustness of these two models for the stable steady state objective.

Supplementary Algorithm 1: Algorithm for model selection with ABC SMC

1 Set population indicator, $t = 0$
2 Set initial epsilon, $\epsilon_t = \inf$
3 Set final epsilon, $\epsilon_F = [x, y, z]$
4 Set particle indicator, $i = 0$
5 **if** $t = 0$ **then**
6   Sample m^* from $\pi(m)$
7   Sample θ^{**} from $\pi(\theta(m^*))$
8 **else if** $t > 0$ **then**
9   Sample particle θ^* from previous population $\{\theta(m^*)^i_{t-1}\}$ with weights $w(m^*)_{t-1}$
10   Perturb θ^* to obtain $\theta^{**} \sim K_t(\theta|\theta^*)$
11   **if** $\pi(\theta^{**}) = 0$ **then**
12   **go to** 5
13
14 Simulate, $x^* \sim f(x|\theta^{**}, m^*)$
15   **if** $d(x^*, x_0) > \epsilon_t$ **then**
16   **go to** 5
17
18 Set $m^i_t = m^*$
19 Set $\theta^i_t = \theta^{**}$
20 Calculate particle weight, w^i_t
21   **if** $t = 0$ **then**
22         $w^i_t = 1$
23   **else**
24         $w^i_t = \frac{\pi(\theta^{**})}{\sum_{j=1}^{N} w^j_{t-1} K_t(\theta^i_{t-1}|\theta^{**})}$
25   **if** $i < N$ **then**
26   Set $i = i + 1$
27   **go to** 5
28
29 Normalise weights for every m.
30 **if** $\epsilon_t \neq \epsilon_T$ **then**
31   Update population number, $t = t + 1$
32   Update ϵ according to accepted particle distances, $\epsilon_t = f_\epsilon()$
33   **go to** 5

Supplementary Figure 4: Two strain model space topologies

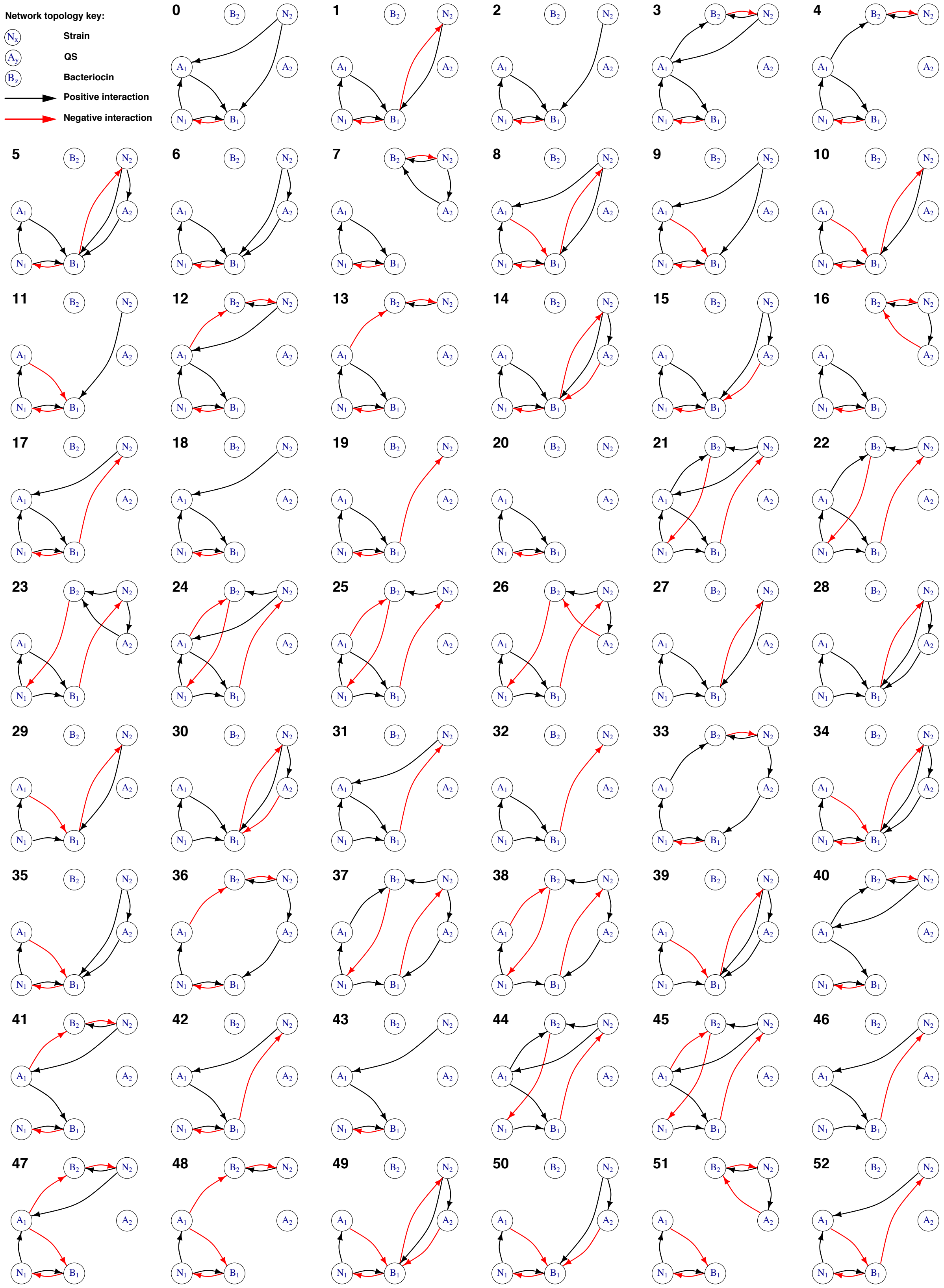

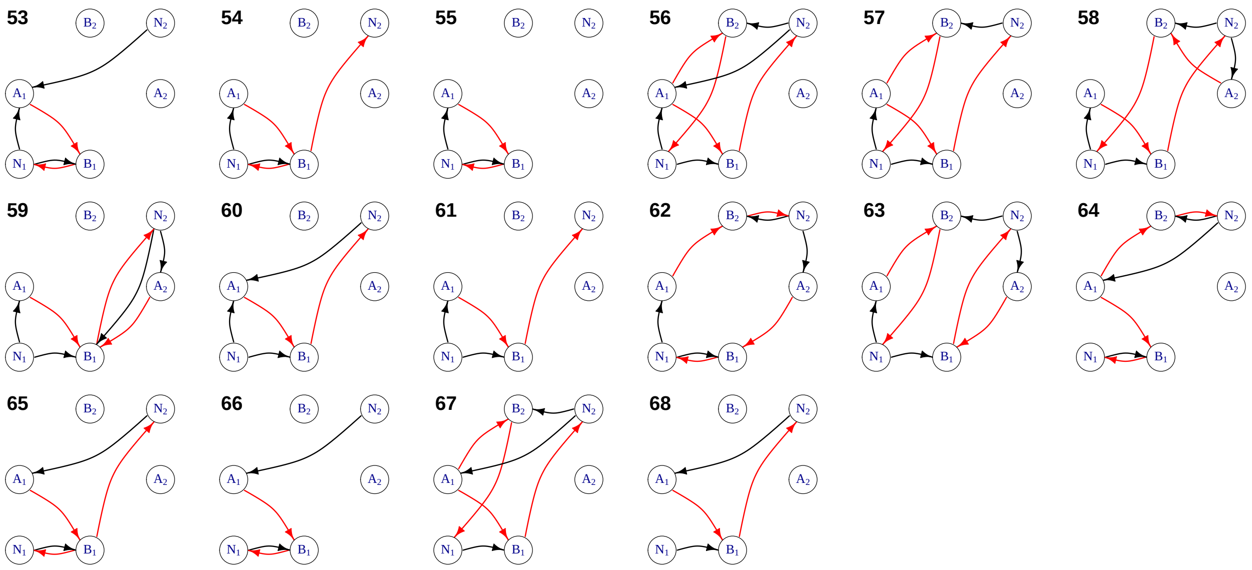
53
54
55
56
57
58
59
60
61
62
63
64
65
66
67
68

Supplementary Figure 5: Three strain model space topologies

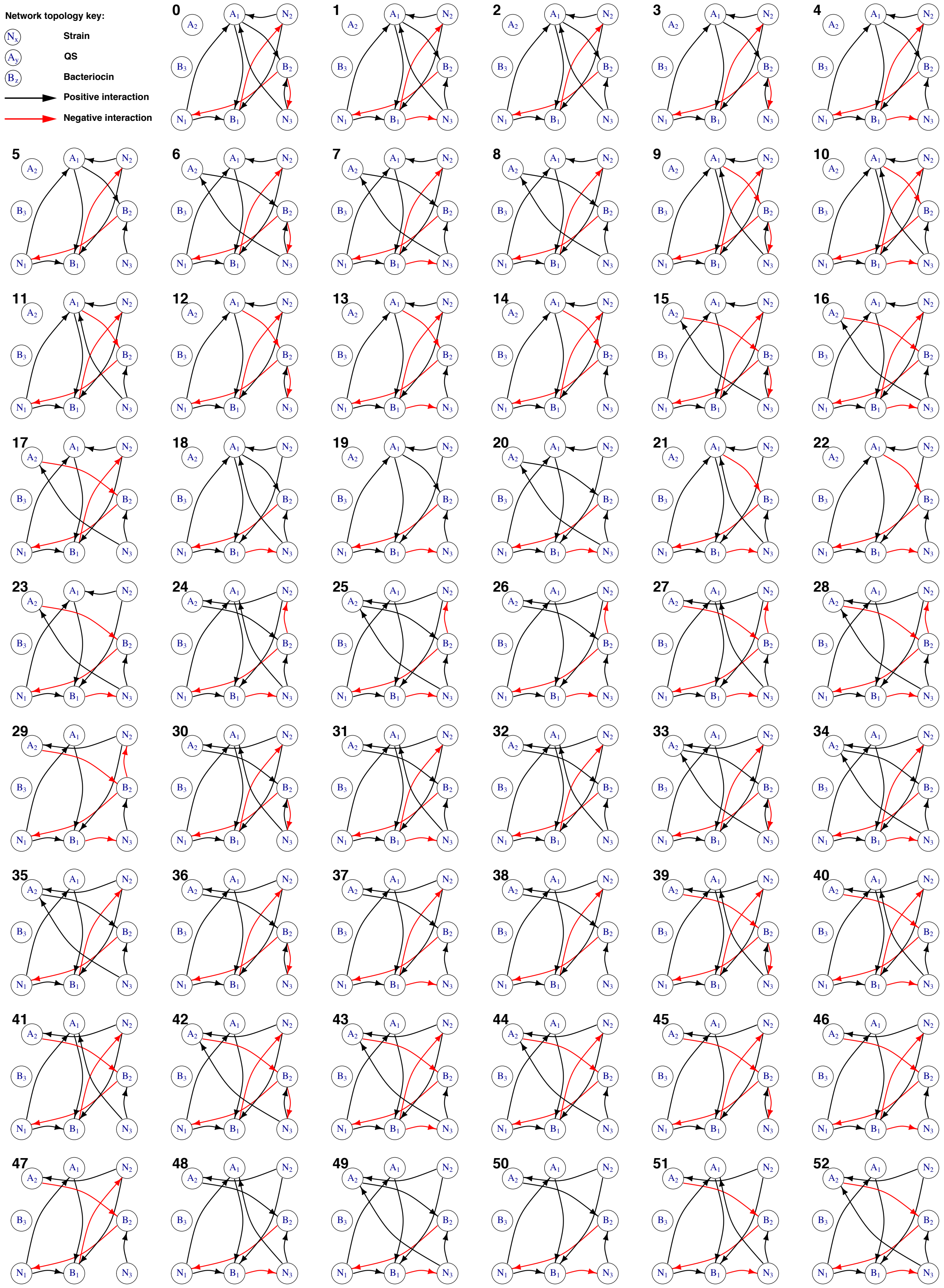

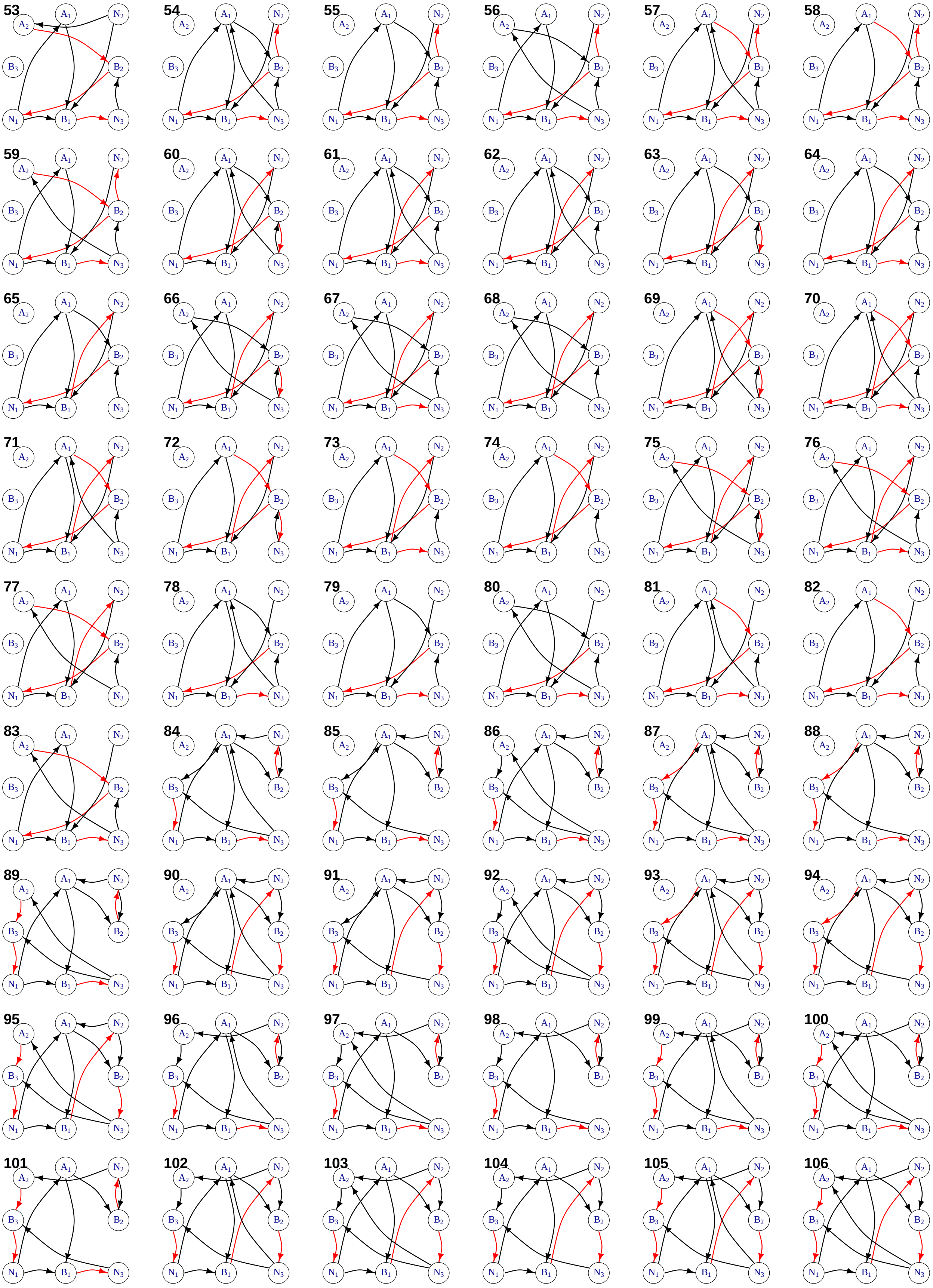
53
54
55
56
57
58
59
60
61
62
63
64
65
66
67
68
69
70
71
72
73
74
75
76
77
78
79
80
81
82
83
84
85
86
87
88
89
90
91
92
93
94
95
96
97
98
99
100
101
102
103
104
105
106

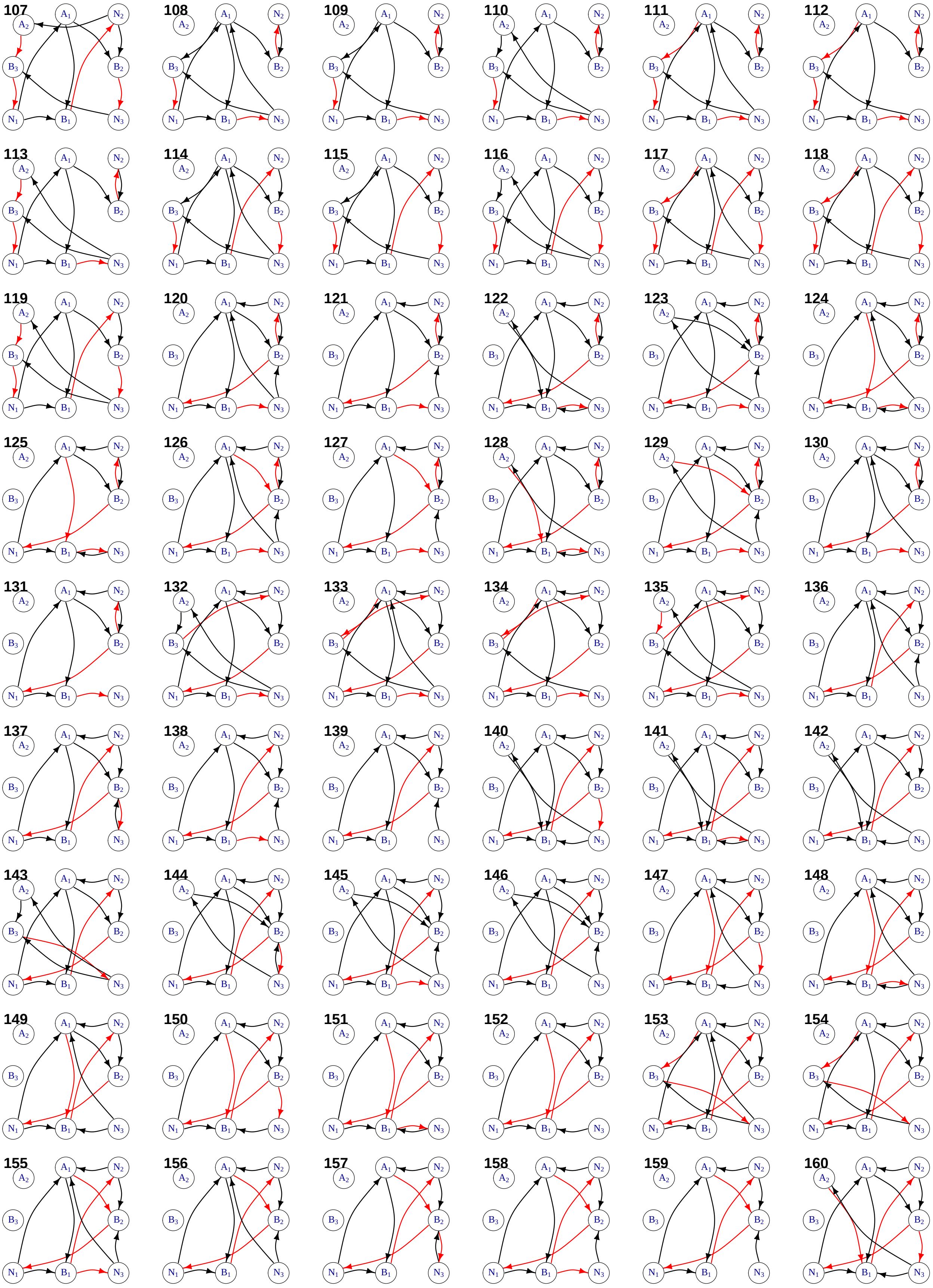
107 108 109 110 111 112
113 114 115 116 117 118
119 120 121 122 123 124
125 126 127 128 129 130
131 132 133 134 135 136
137 138 139 140 141 142
143 144 145 146 147 148
149 150 151 152 153 154
155 156 157 158 159 160

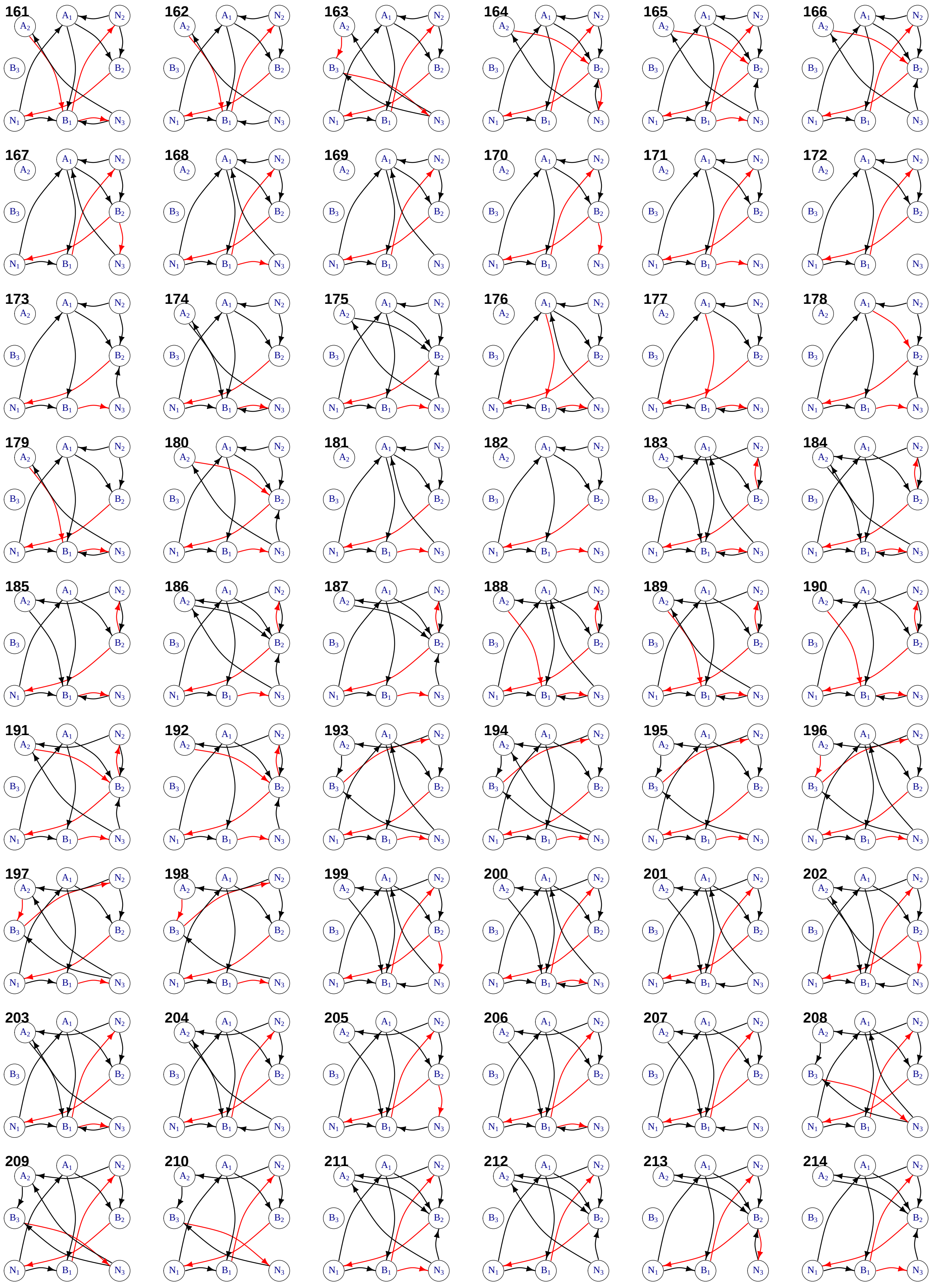
161
162
163
164
165
166
167
168
169
170
171
172
173
174
175
176
177
178
179
180
181
182
183
184
185
186
187
188
189
190
191
192
193
194
195
196
197
198
199
200
201
202
203
204
205
206
207
208
209
210
211
212
213
214

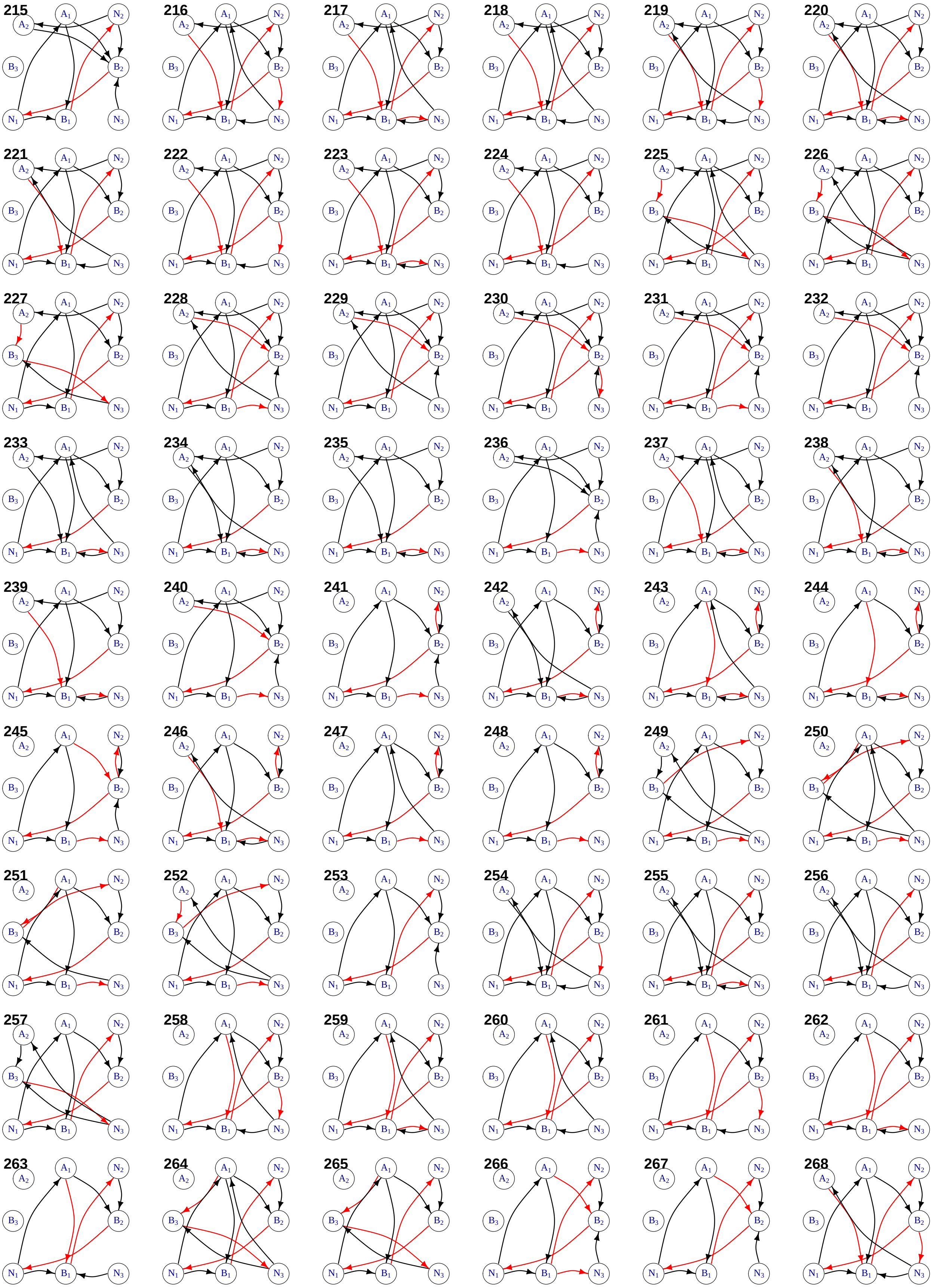
215 216 217 218 219 220
221 222 223 224 225 226
227 228 229 230 231 232
233 234 235 236 237 238
239 240 241 242 243 244
245 246 247 248 249 250
251 252 253 254 255 256
257 258 259 260 261 262
263 264 265 266 267 268

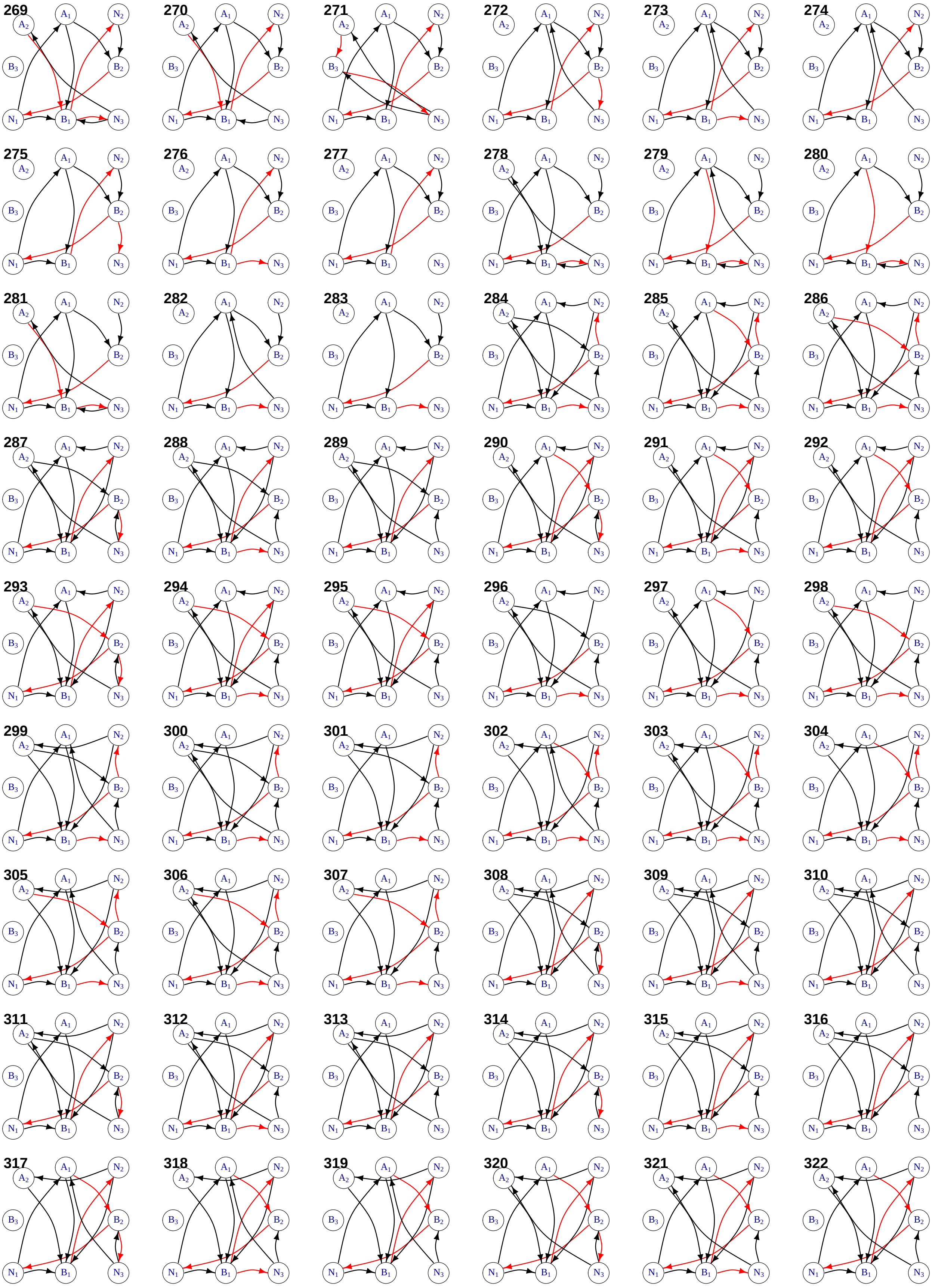
269
270
271
272
273
274
275
276
277
278
279
280
281
282
283
284
285
286
287
288
289
290
291
292
293
294
295
296
297
298
299
300
301
302
303
304
305
306
307
308
309
310
311
312
313
314
315
316
317
318
319
320
321
322

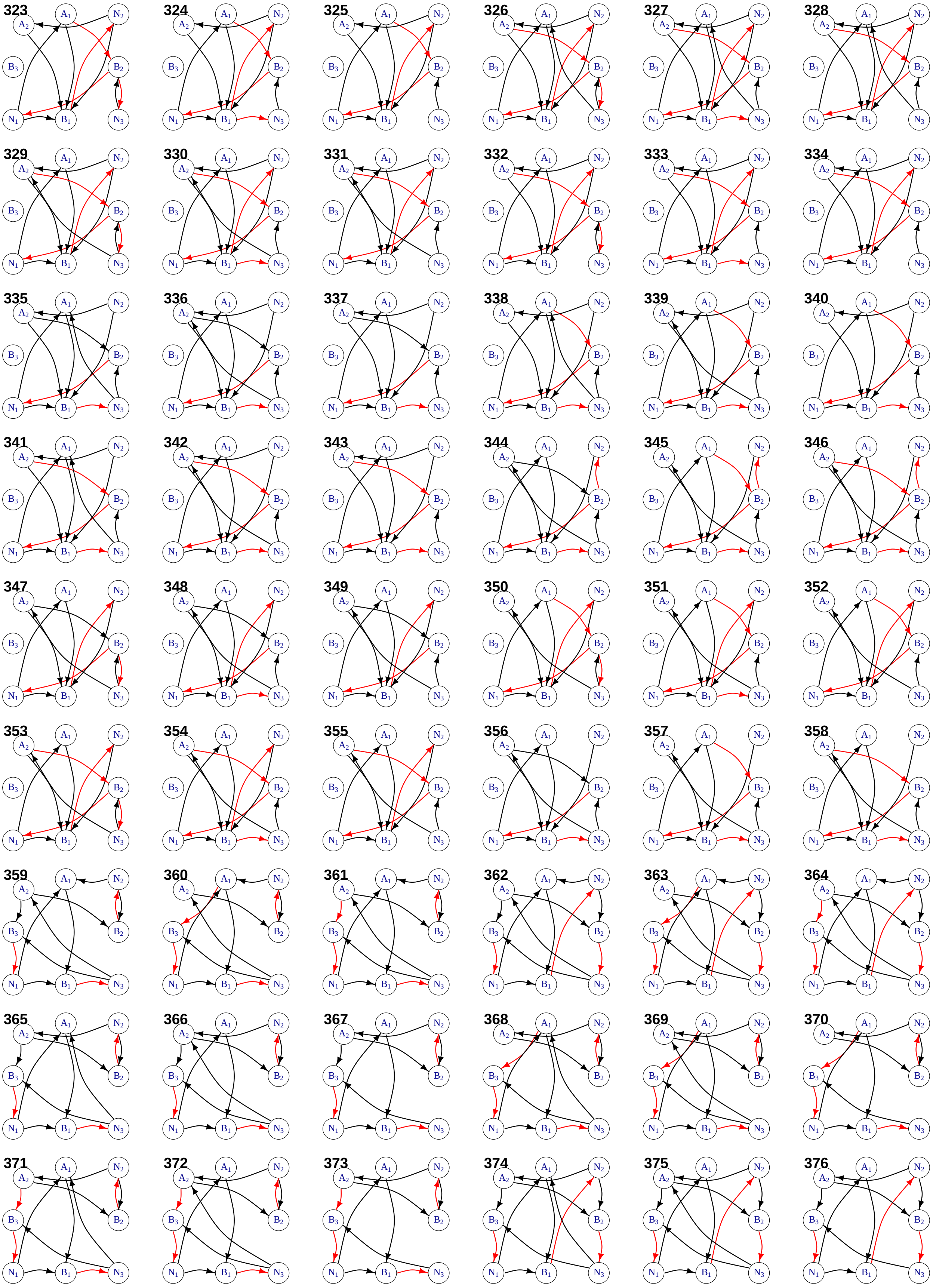
323
324
325
326
327
328
329
330
331
332
333
334
335
336
337
338
339
340
341
342
343
344
345
346
347
348
349
350
351
352
353
354
355
356
357
358
359
360
361
362
363
364
365
366
367
368
369
370
371
372
373
374
375
376
A1
A2
B1
B2
B3
N1
N2
N3

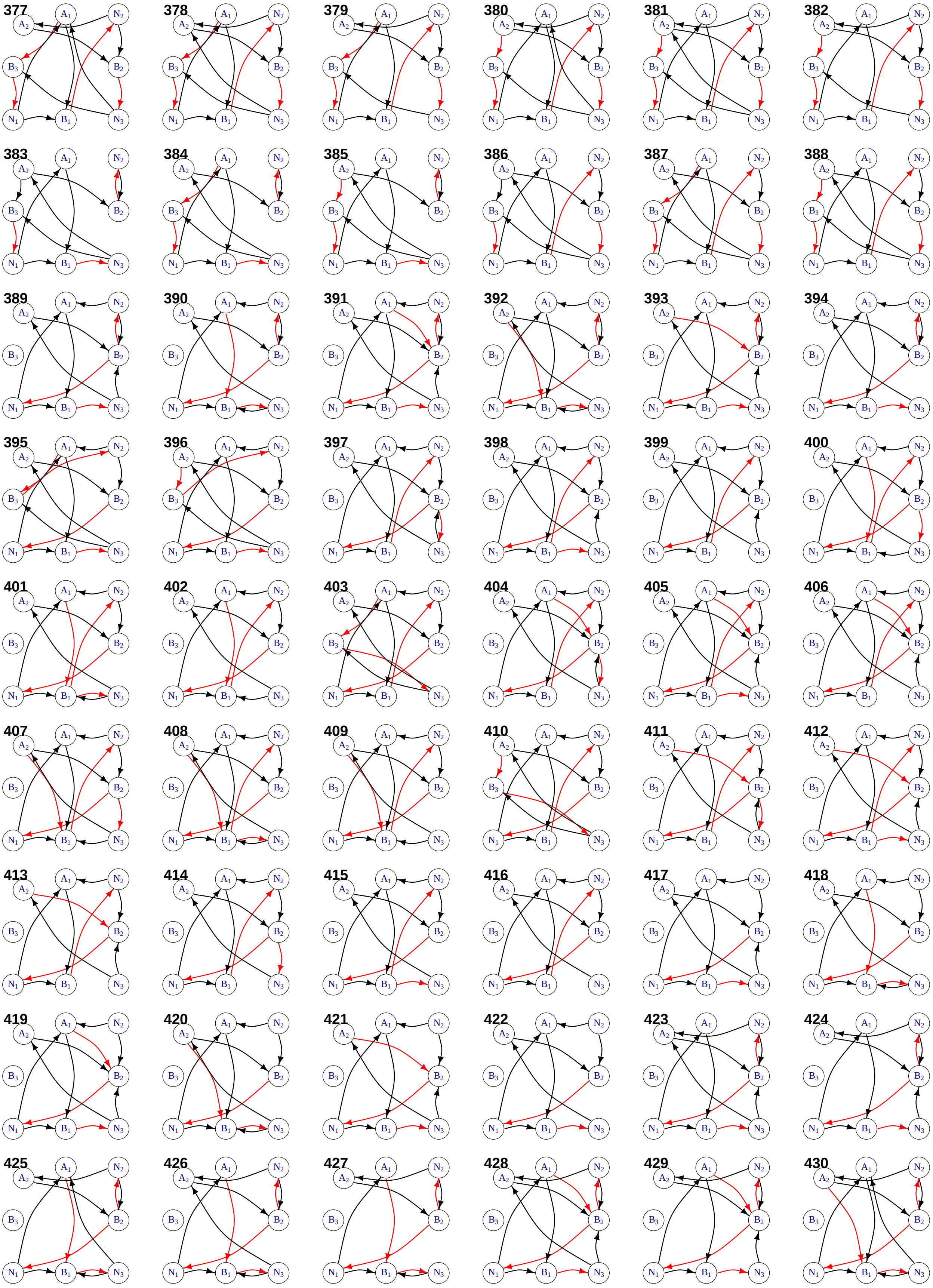
377 378 379 380 381 382
383 384 385 386 387 388
389 390 391 392 393 394
395 396 397 398 399 400
401 402 403 404 405 406
407 408 409 410 411 412
413 414 415 416 417 418
419 420 421 422 423 424
425 426 427 428 429 430
A1 A2 B1 B2 B3 N1 N2 N3

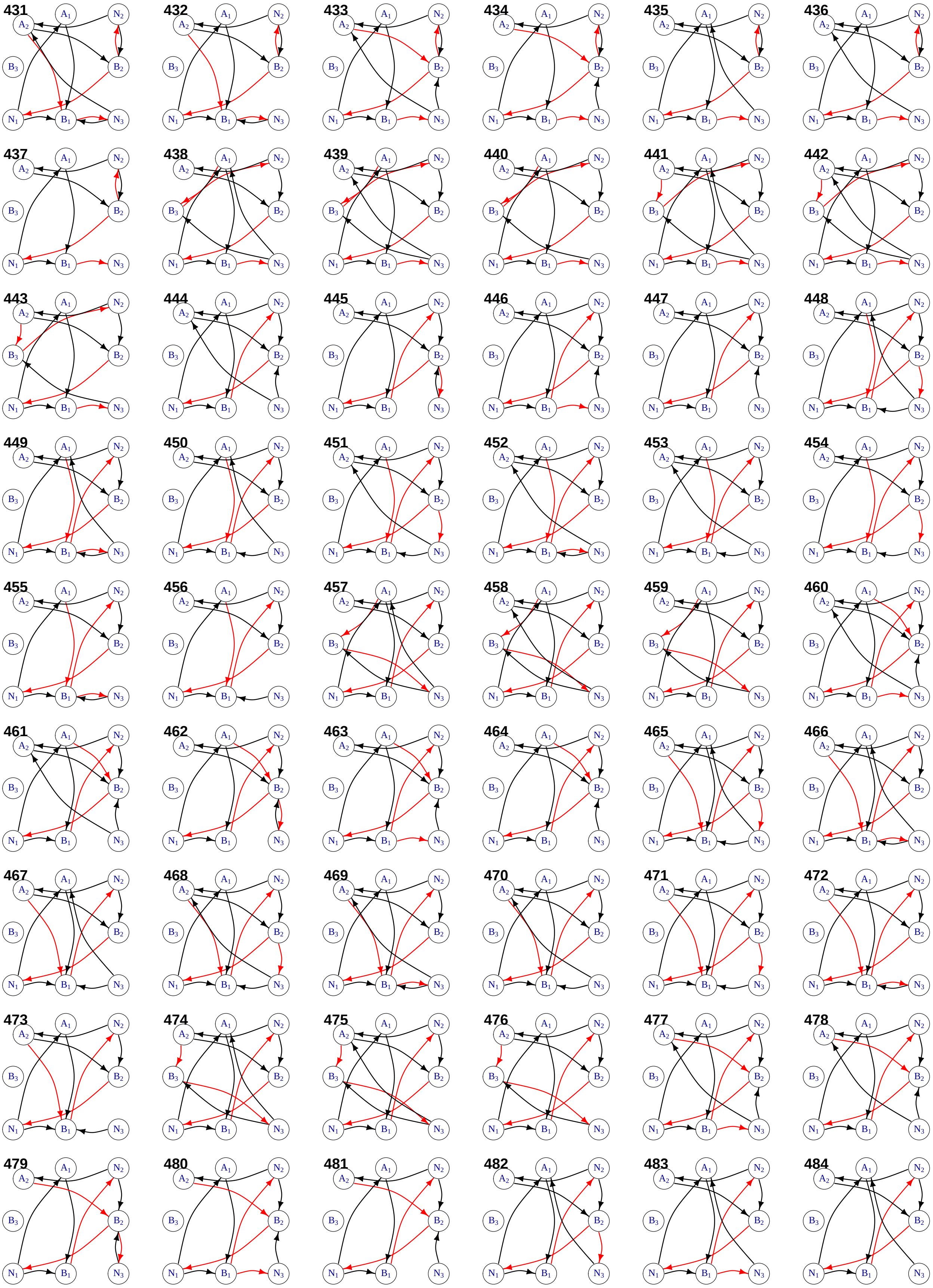
431
432
433
434
435
436
437
438
439
440
441
442
443
444
445
446
447
448
449
450
451
452
453
454
455
456
457
458
459
460
461
462
463
464
465
466
467
468
469
470
471
472
473
474
475
476
477
478
479
480
481
482
483
484

485 486 487 488 489 490
491 492 493 494 495 496
497 498 499 500 501 502
503 504 505 506 507 508
509 510 511 512 513 514
515 516 517 518 519 520
521 522 523 524 525 526
527 528 529 530 531 532
533 534 535 536 537 538

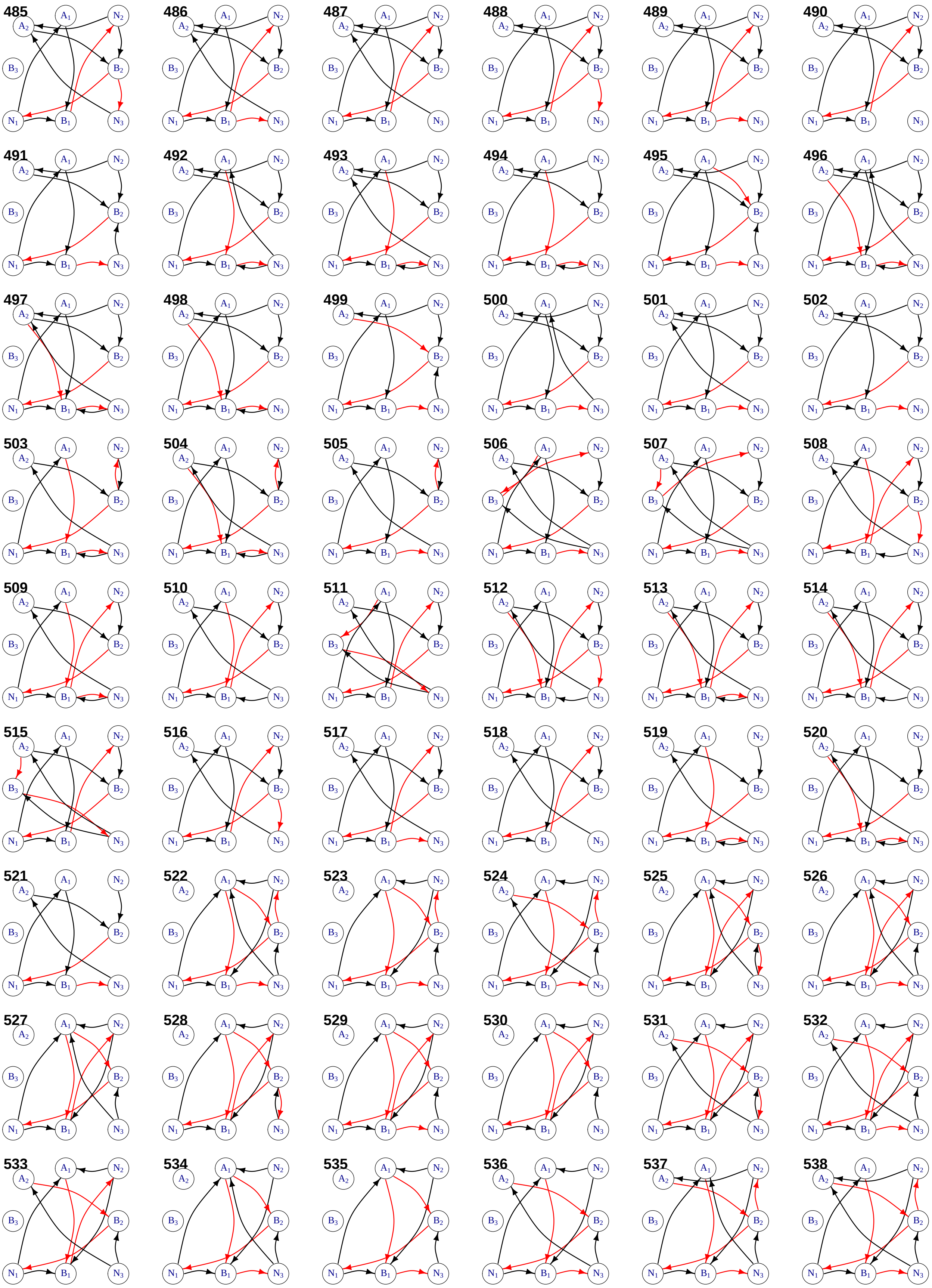

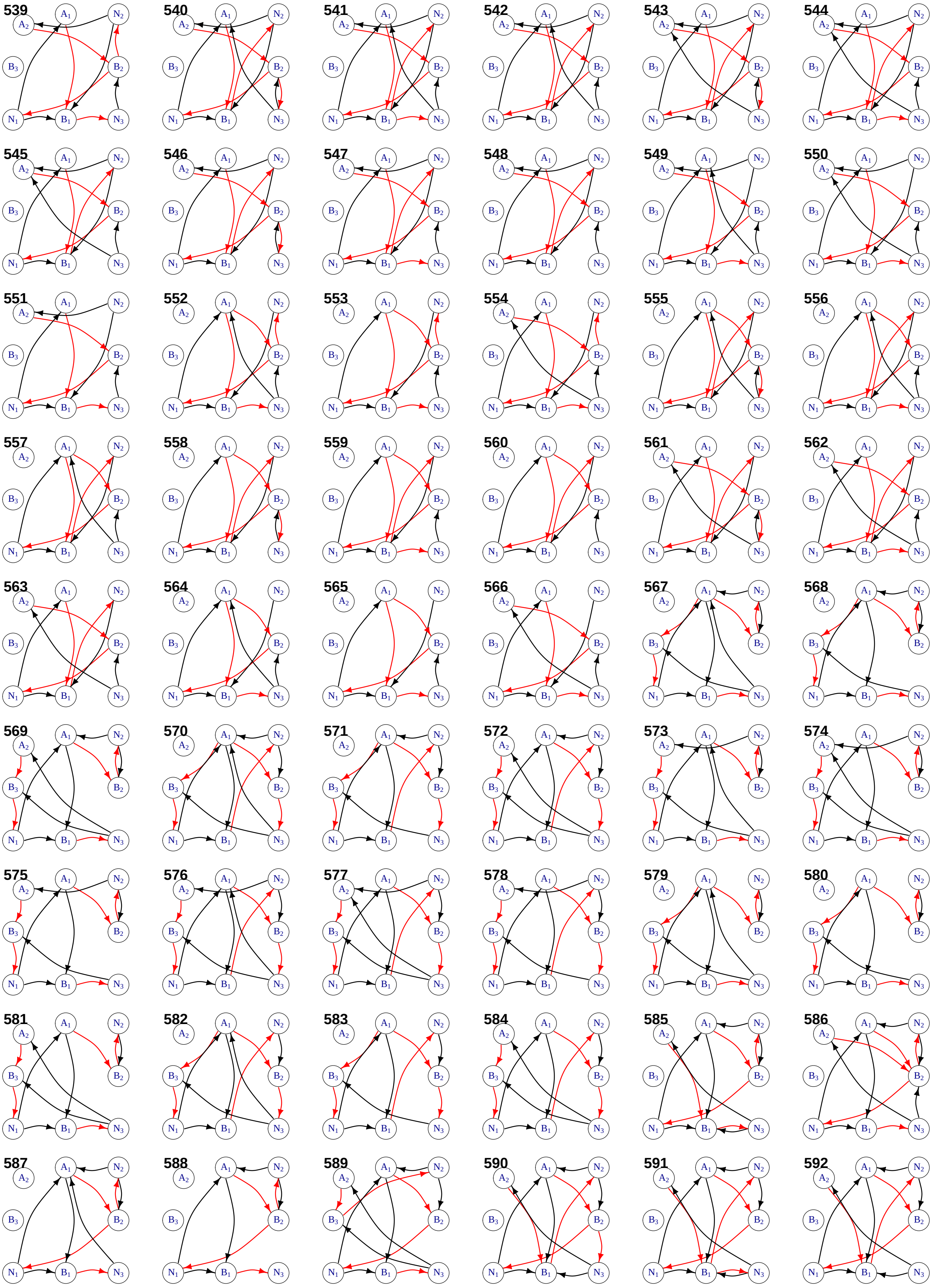
539 540 541 542 543 544
545 546 547 548 549 550
551 552 553 554 555 556
557 558 559 560 561 562
563 564 565 566 567 568
569 570 571 572 573 574
575 576 577 578 579 580
581 582 583 584 585 586
587 588 589 590 591 592

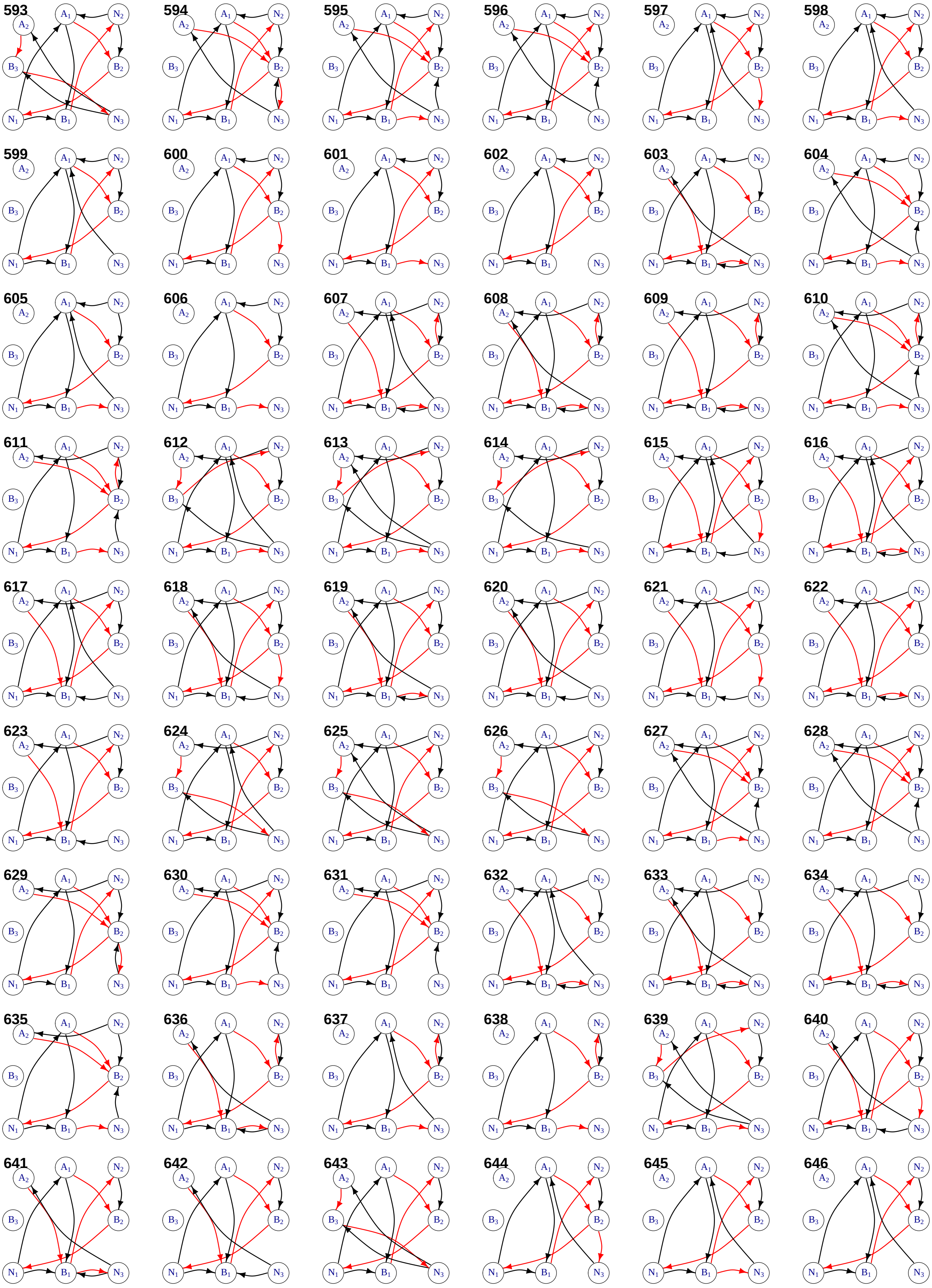
593
594
595
596
597
598
599
600
601
602
603
604
605
606
607
608
609
610
611
612
613
614
615
616
617
618
619
620
621
622
623
624
625
626
627
628
629
630
631
632
633
634
635
636
637
638
639
640
641
642
643
644
645
646

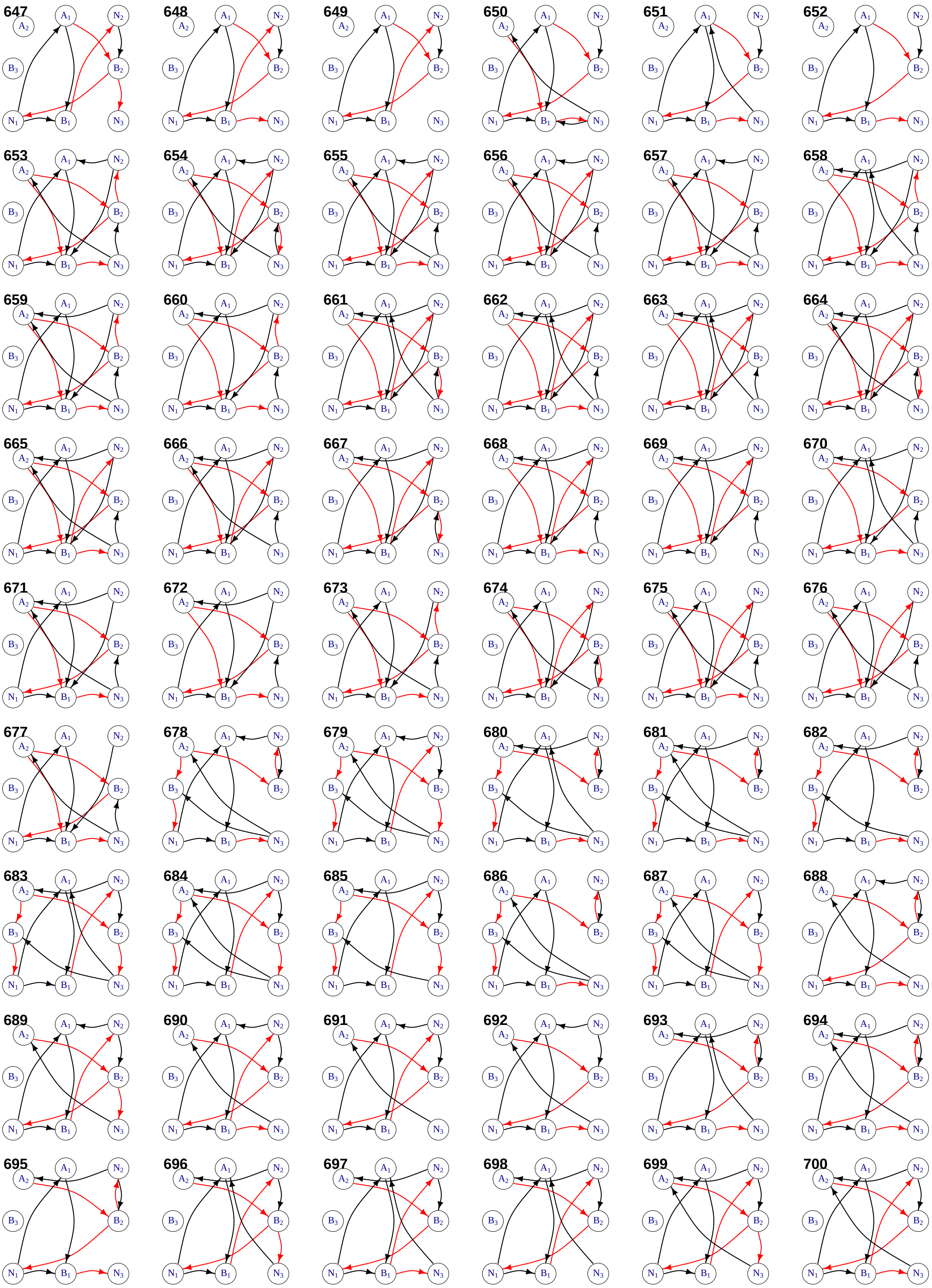
647
648
649
650
651
652
653
654
655
656
657
658
659
660
661
662
663
664
665
666
667
668
669
670
671
672
673
674
675
676
677
678
679
680
681
682
683
684
685
686
687
688
689
690
691
692
693
694
695
696
697
698
699
700

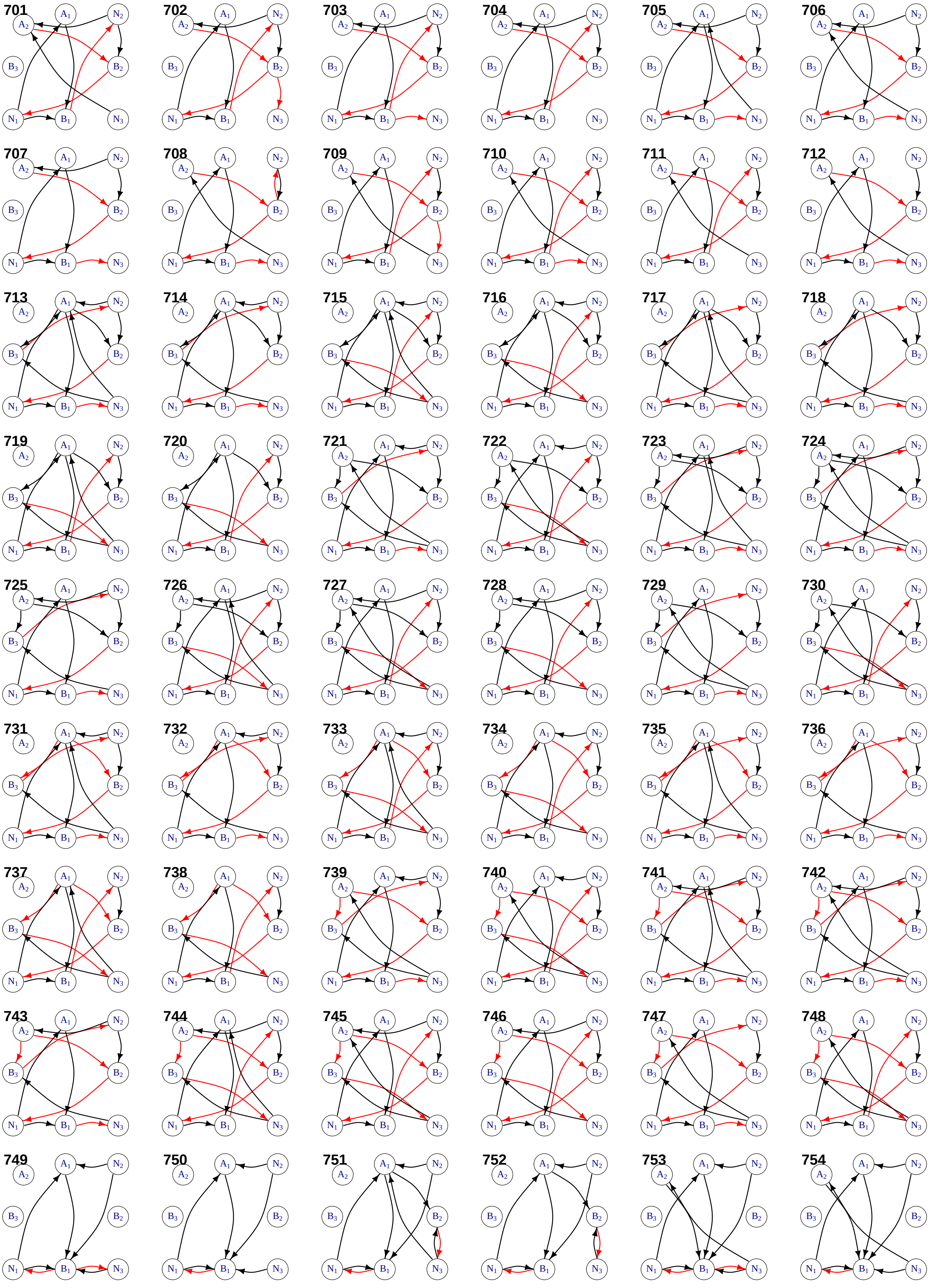
701 702 703 704 705 706
707 708 709 710 711 712
713 714 715 716 717 718
719 720 721 722 723 724
725 726 727 728 729 730
731 732 733 734 735 736
737 738 739 740 741 742
743 744 745 746 747 748
749 750 751 752 753 754

755 756 757 758 759 760

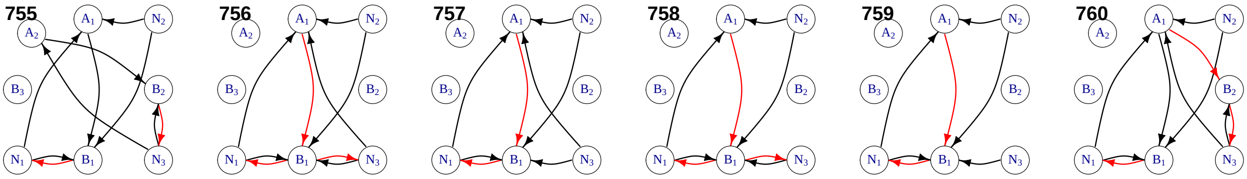

761 762 763 764 765 766

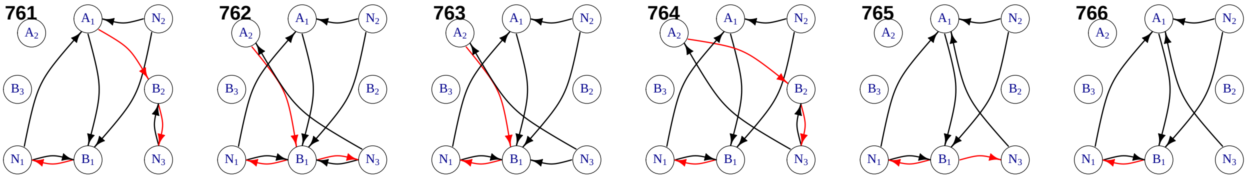

767 768 769 770 771 772

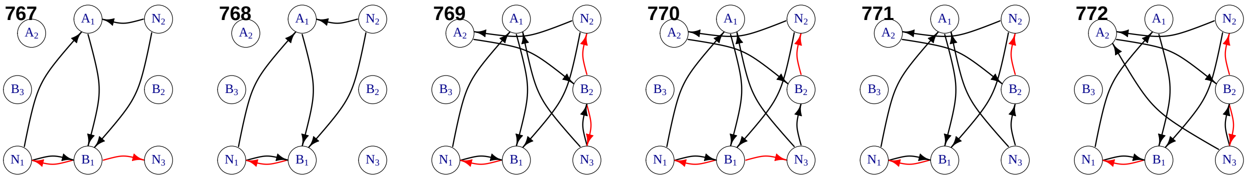

773 774 775 776 777 778

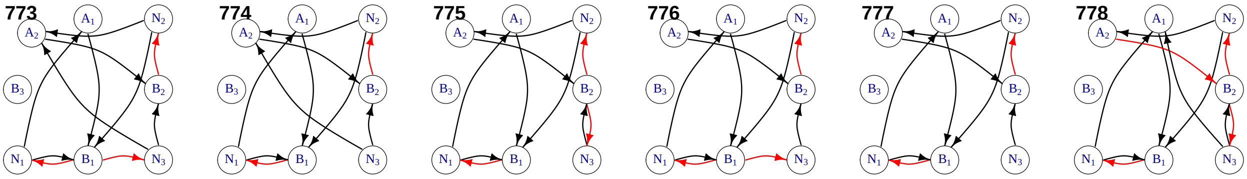

779 780 781 782 783 784

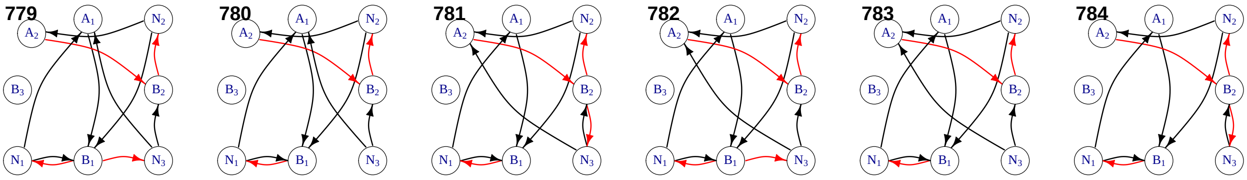

785 786 787 788 789 790

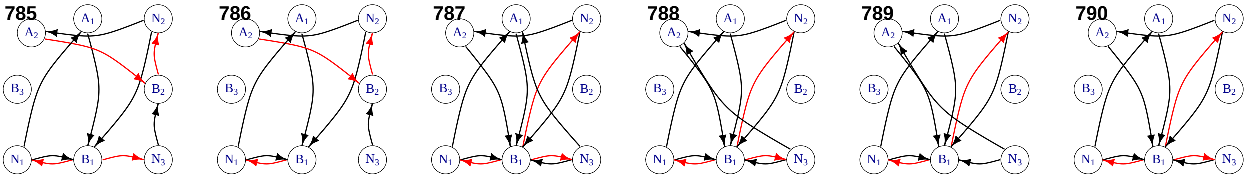

791 792 793 794 795 796

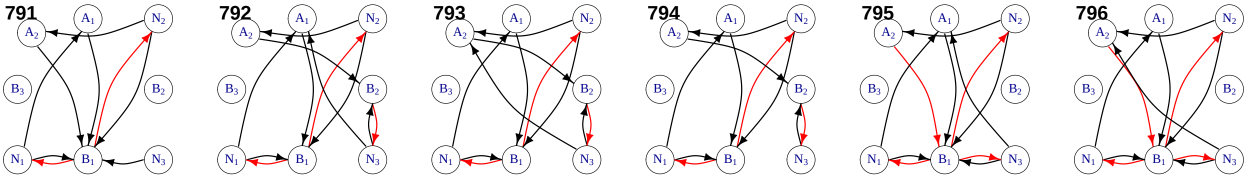

797 798 799 800 801 802

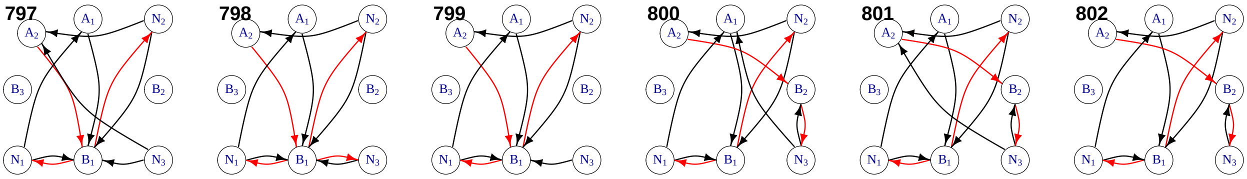

803 804 805 806 807 808

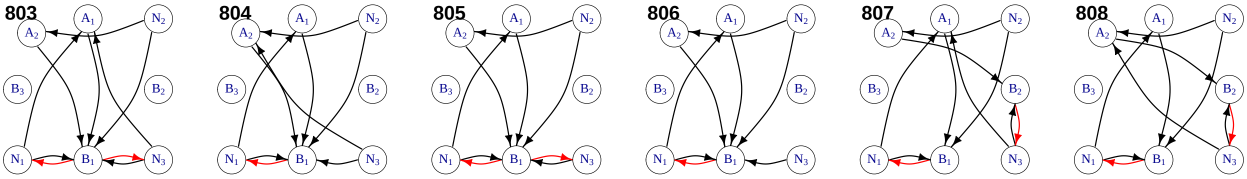

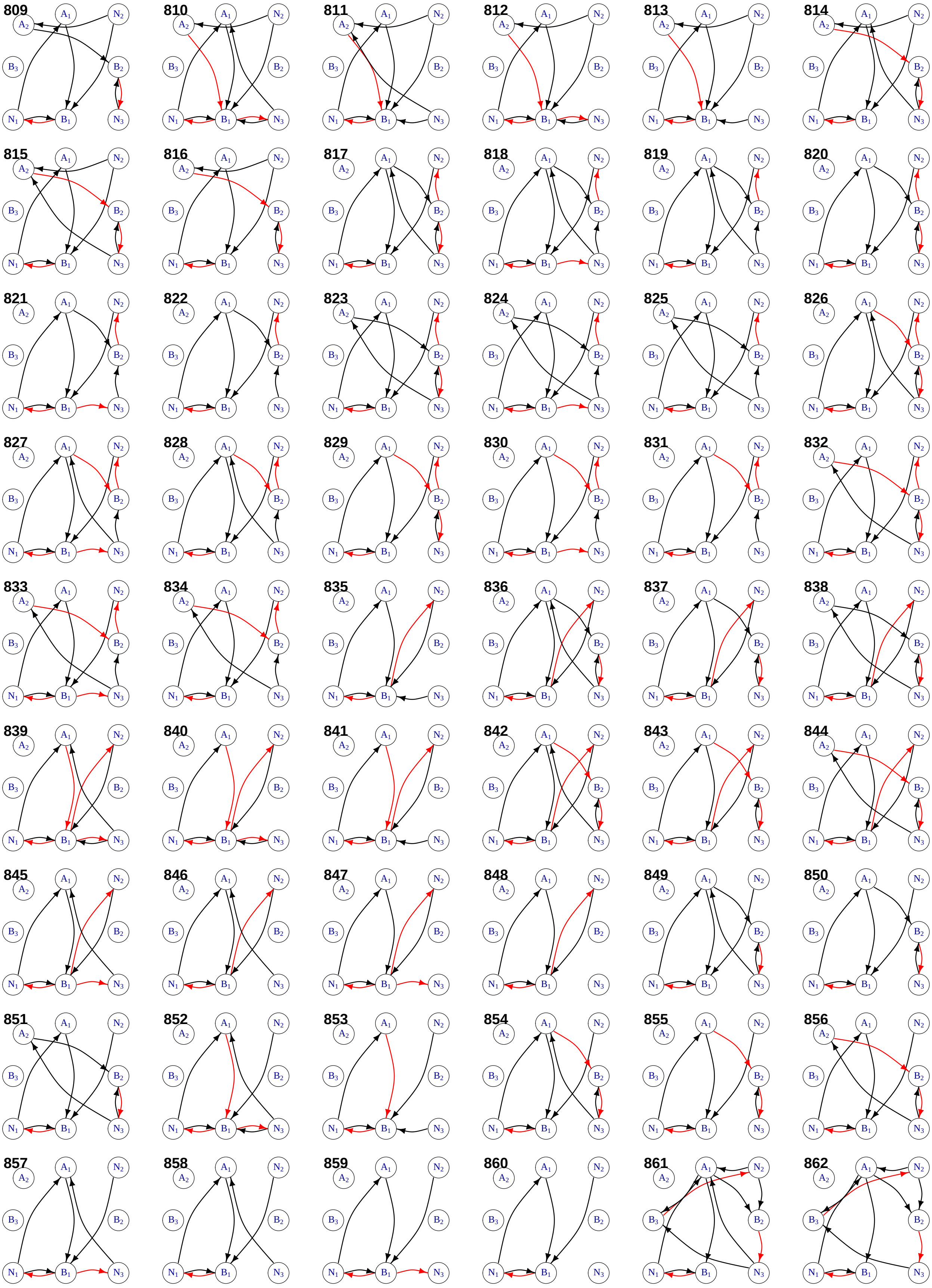
809
810
811
812
813
814
815
816
817
818
819
820
821
822
823
824
825
826
827
828
829
830
831
832
833
834
835
836
837
838
839
840
841
842
843
844
845
846
847
848
849
850
851
852
853
854
855
856
857
858
859
860
861
862

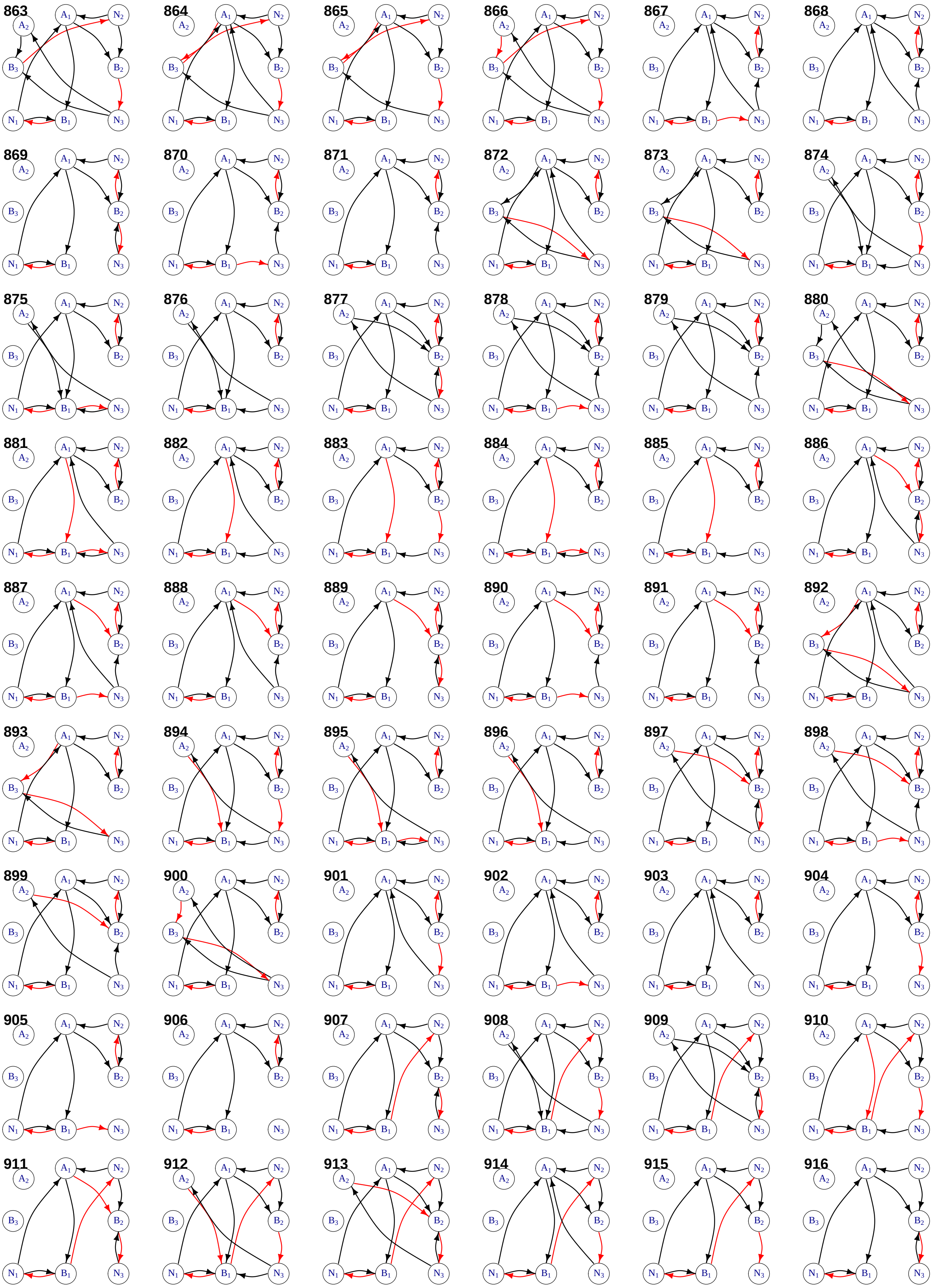

863
864
865
866
867
868
869
870
871
872
873
874
875
876
877
878
879
880
881
882
883
884
885
886
887
888
889
890
891
892
893
894
895
896
897
898
899
900
901
902
903
904
905
906
907
908
909
910
911
912
913
914
915
916

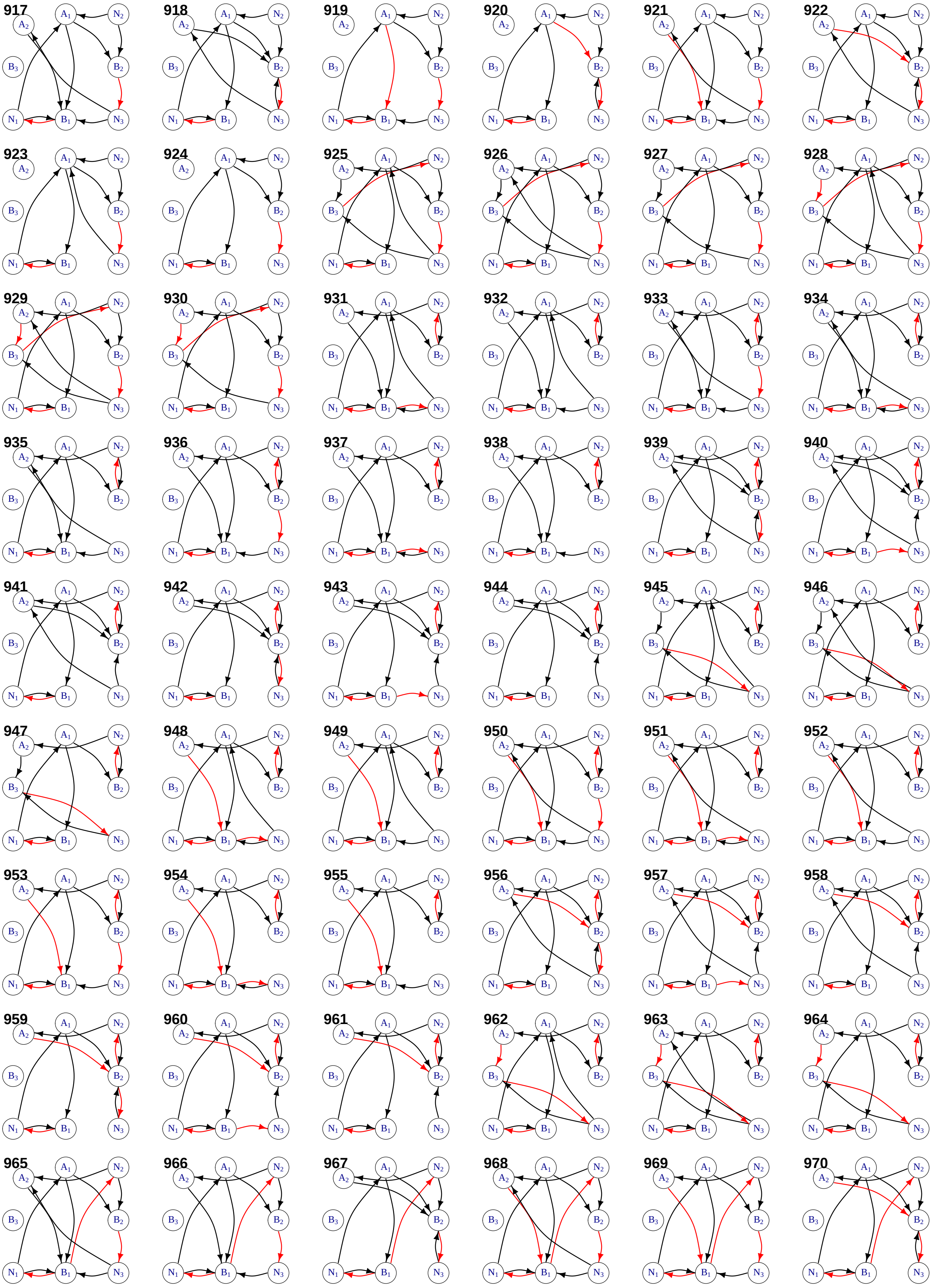
917
918
919
920
921
922
923
924
925
926
927
928
929
930
931
932
933
934
935
936
937
938
939
940
941
942
943
944
945
946
947
948
949
950
951
952
953
954
955
956
957
958
959
960
961
962
963
964
965
966
967
968
969
970

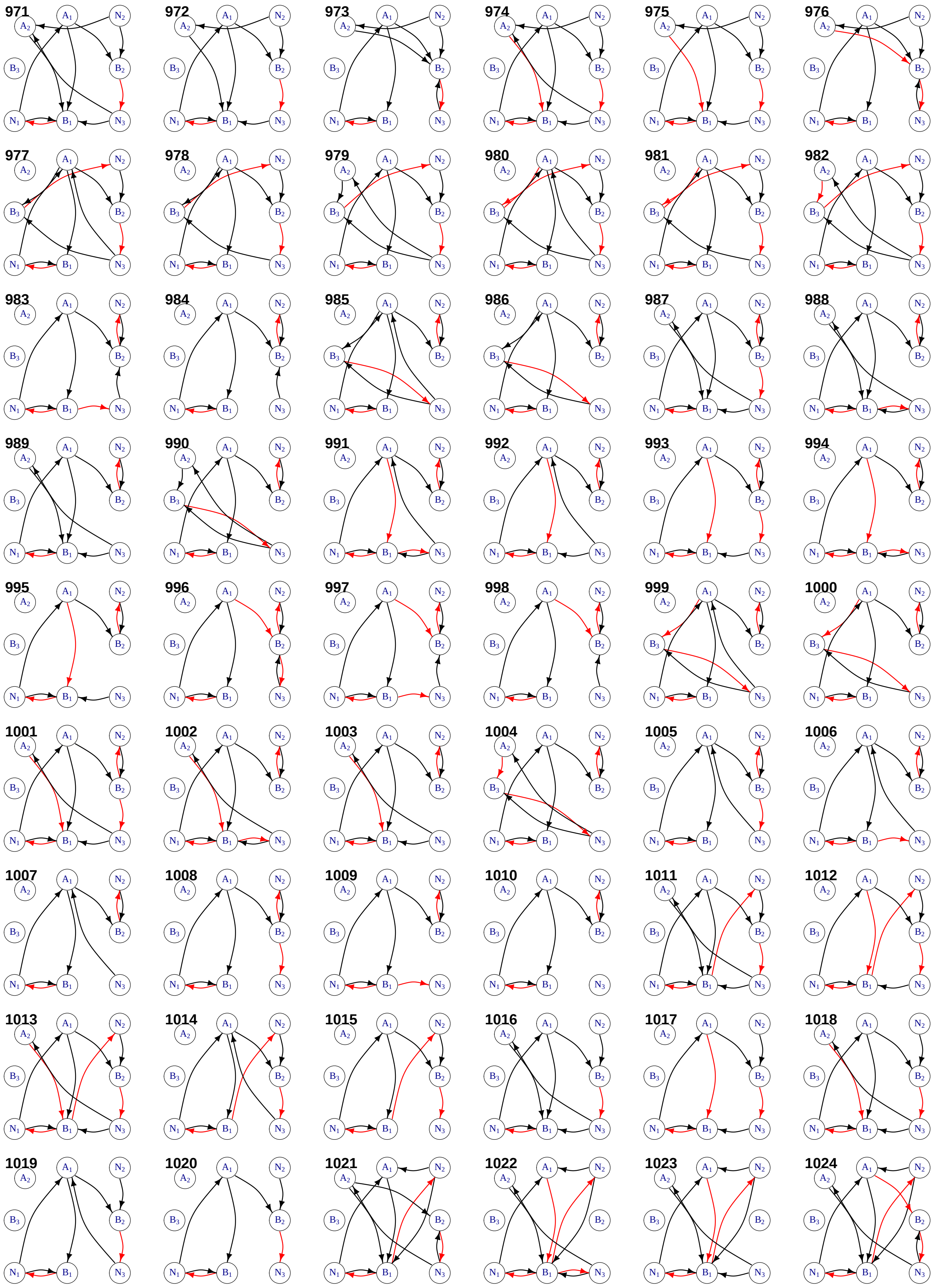
971 972 973 974 975 976
977 978 979 980 981 982
983 984 985 986 987 988
989 990 991 992 993 994
995 996 997 998 999 1000
1001 1002 1003 1004 1005 1006
1007 1008 1009 1010 1011 1012
1013 1014 1015 1016 1017 1018
1019 1020 1021 1022 1023 1024
A₁ A₂ B₁ B₂ B₃ N₁ N₂ N₃

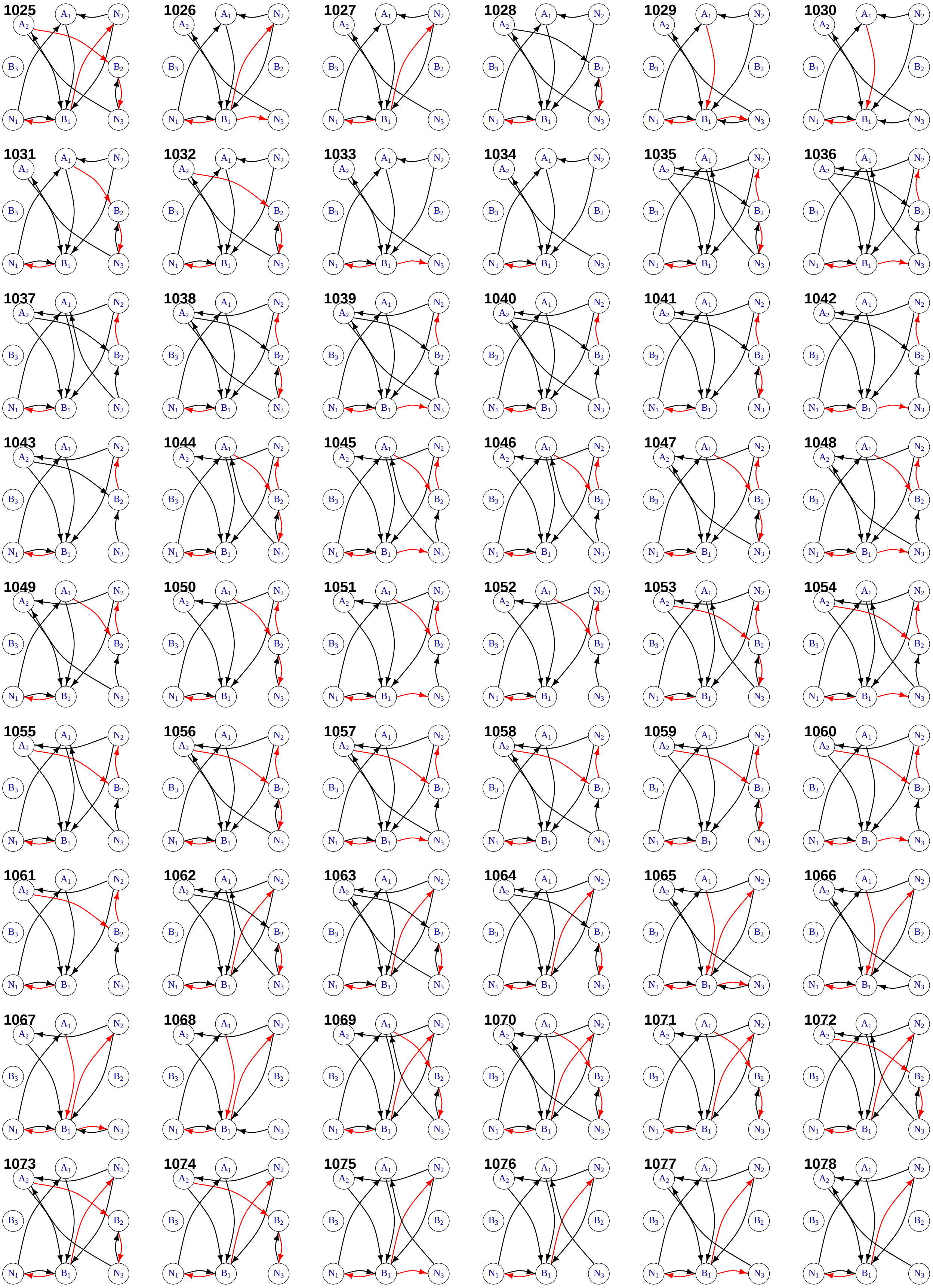
1025 1026 1027 1028 1029 1030
1031 1032 1033 1034 1035 1036
1037 1038 1039 1040 1041 1042
1043 1044 1045 1046 1047 1048
1049 1050 1051 1052 1053 1054
1055 1056 1057 1058 1059 1060
1061 1062 1063 1064 1065 1066
1067 1068 1069 1070 1071 1072
1073 1074 1075 1076 1077 1078

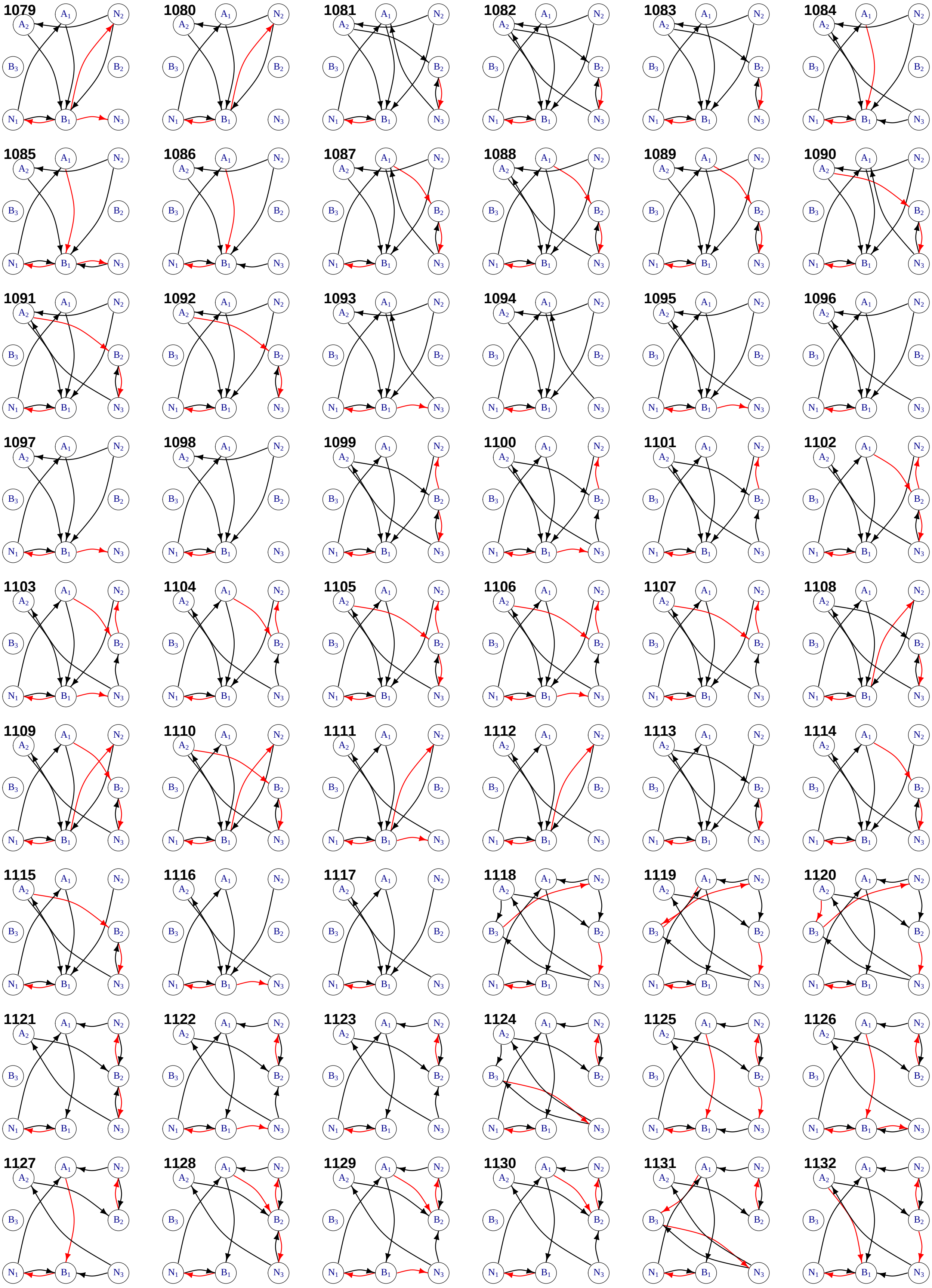

1079
1080
1081
1082
1083
1084
1085
1086
1087
1088
1089
1090
1091
1092
1093
1094
1095
1096
1097
1098
1099
1100
1101
1102
1103
1104
1105
1106
1107
1108
1109
1110
1111
1112
1113
1114
1115
1116
1117
1118
1119
1120
1121
1122
1123
1124
1125
1126
1127
1128
1129
1130
1131
1132

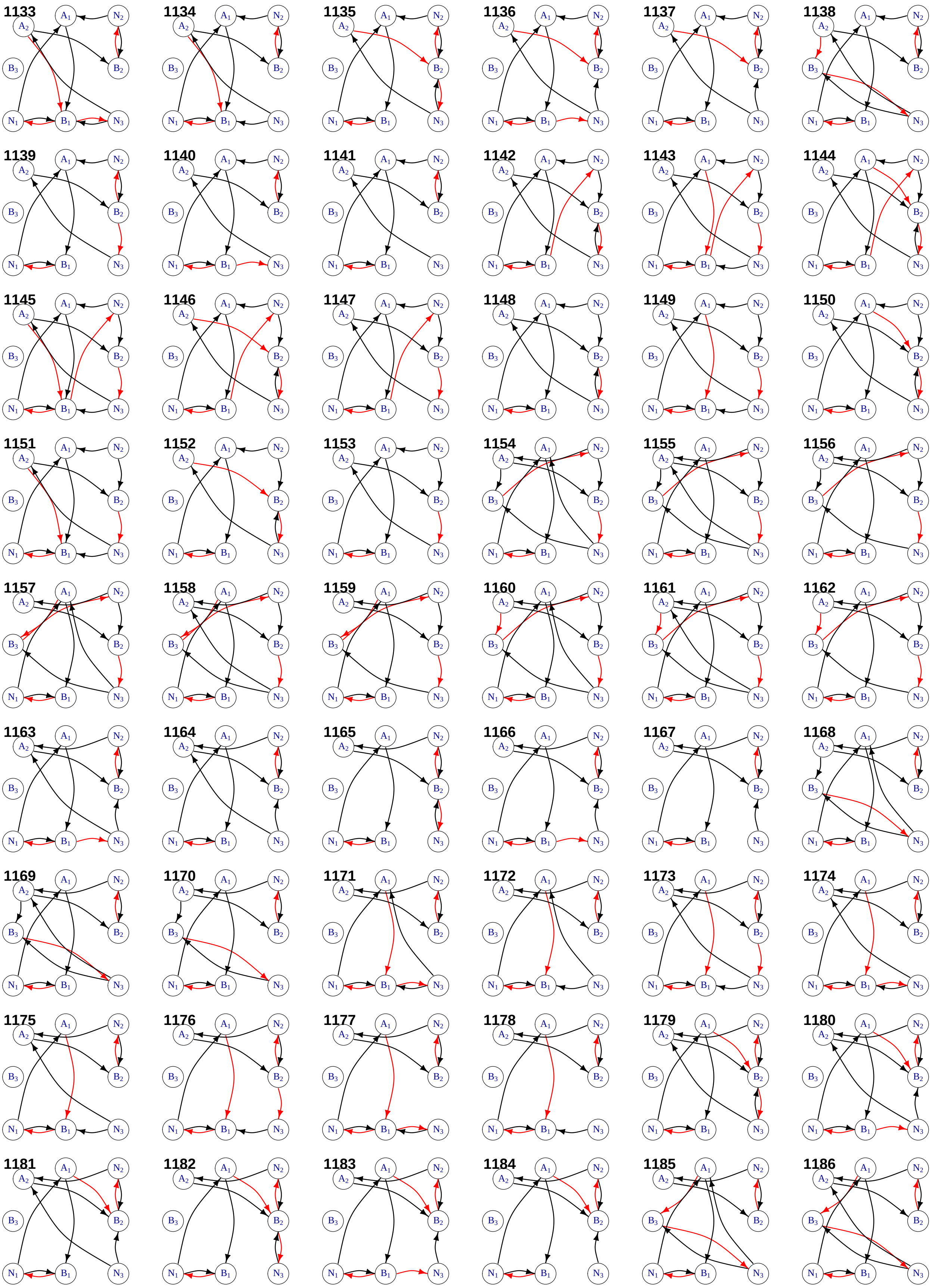
1133
1134
1135
1136
1137
1138
1139
1140
1141
1142
1143
1144
1145
1146
1147
1148
1149
1150
1151
1152
1153
1154
1155
1156
1157
1158
1159
1160
1161
1162
1163
1164
1165
1166
1167
1168
1169
1170
1171
1172
1173
1174
1175
1176
1177
1178
1179
1180
1181
1182
1183
1184
1185
1186

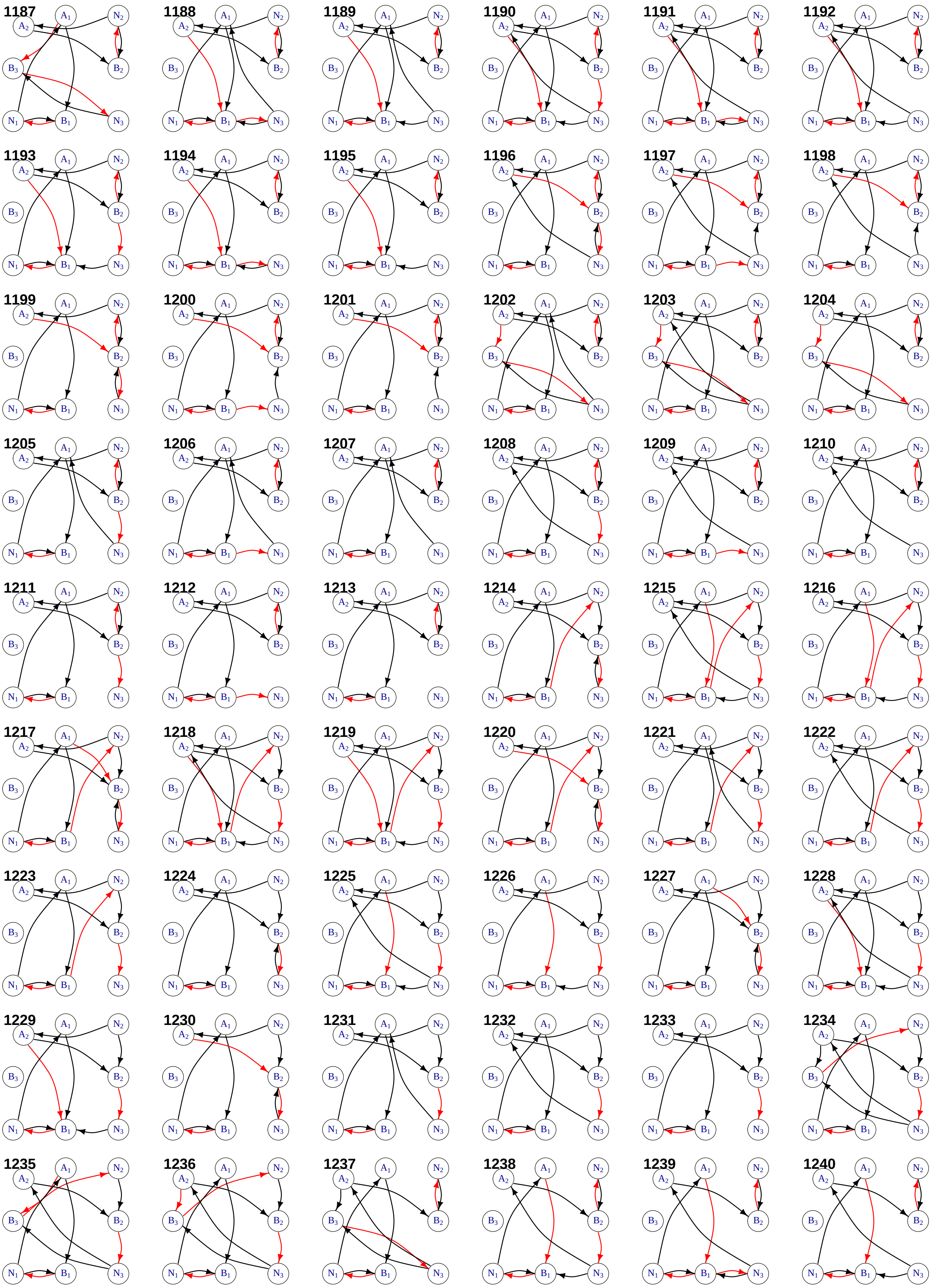
1187 1188 1189 1190 1191 1192
1193 1194 1195 1196 1197 1198
1199 1200 1201 1202 1203 1204
1205 1206 1207 1208 1209 1210
1211 1212 1213 1214 1215 1216
1217 1218 1219 1220 1221 1222
1223 1224 1225 1226 1227 1228
1229 1230 1231 1232 1233 1234
1235 1236 1237 1238 1239 1240

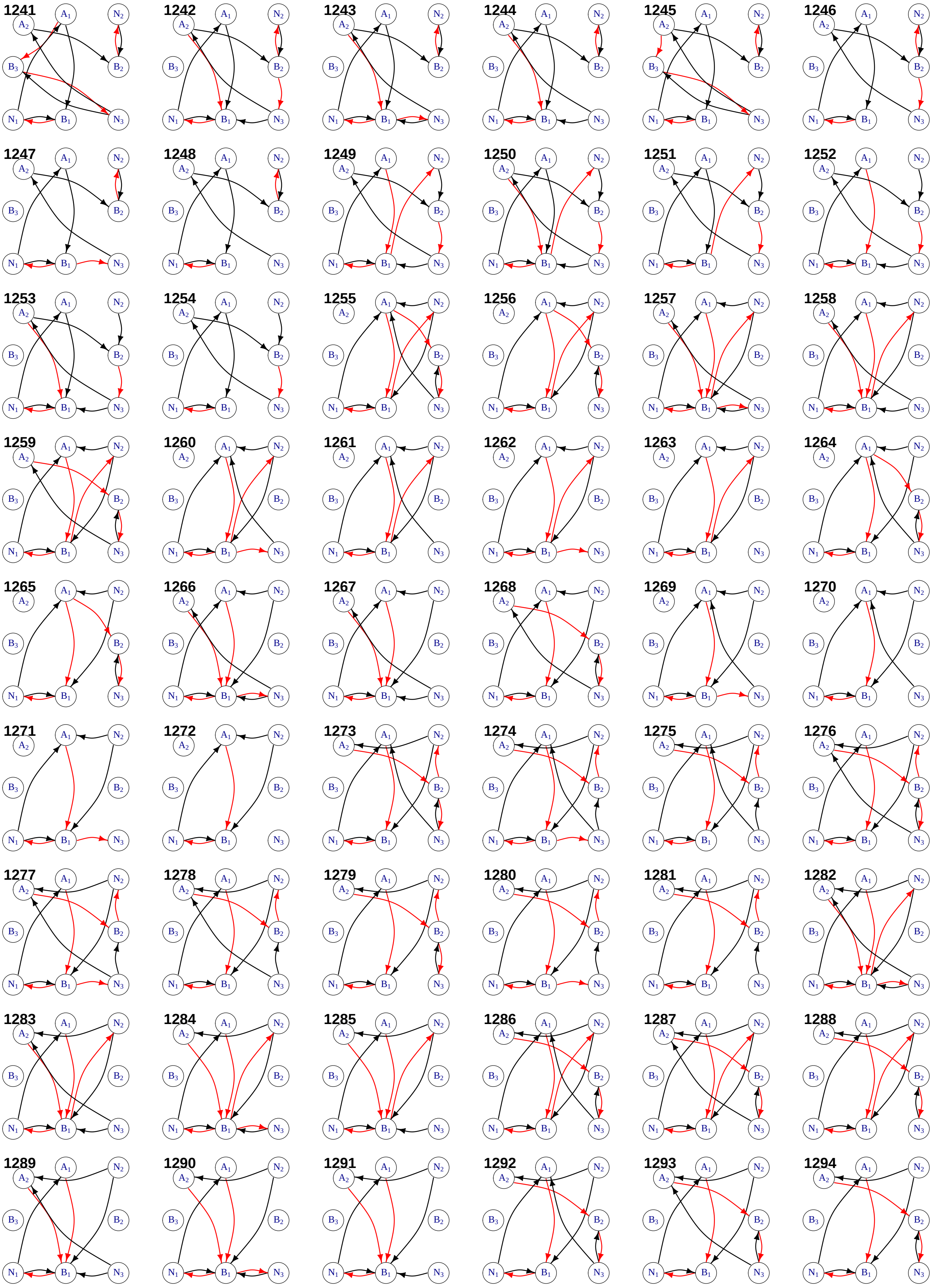
1241 1242 1243 1244 1245 1246
1247 1248 1249 1250 1251 1252
1253 1254 1255 1256 1257 1258
1259 1260 1261 1262 1263 1264
1265 1266 1267 1268 1269 1270
1271 1272 1273 1274 1275 1276
1277 1278 1279 1280 1281 1282
1283 1284 1285 1286 1287 1288
1289 1290 1291 1292 1293 1294

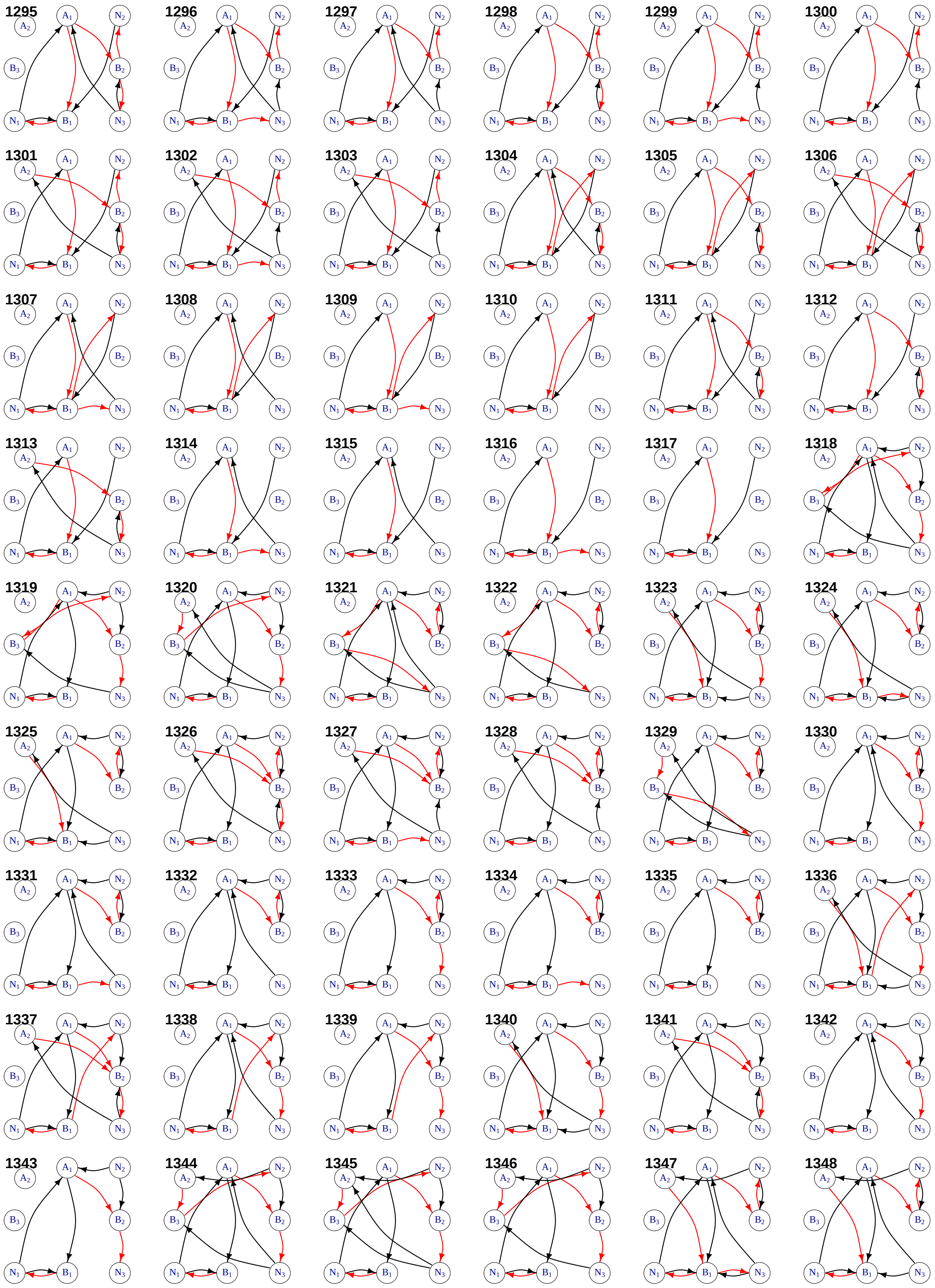
1295 1296 1297 1298 1299 1300
1301 1302 1303 1304 1305 1306
1307 1308 1309 1310 1311 1312
1313 1314 1315 1316 1317 1318
1319 1320 1321 1322 1323 1324
1325 1326 1327 1328 1329 1330
1331 1332 1333 1334 1335 1336
1337 1338 1339 1340 1341 1342
1343 1344 1345 1346 1347 1348
A1 A2 B1 B2 B3 N1 N2 N3

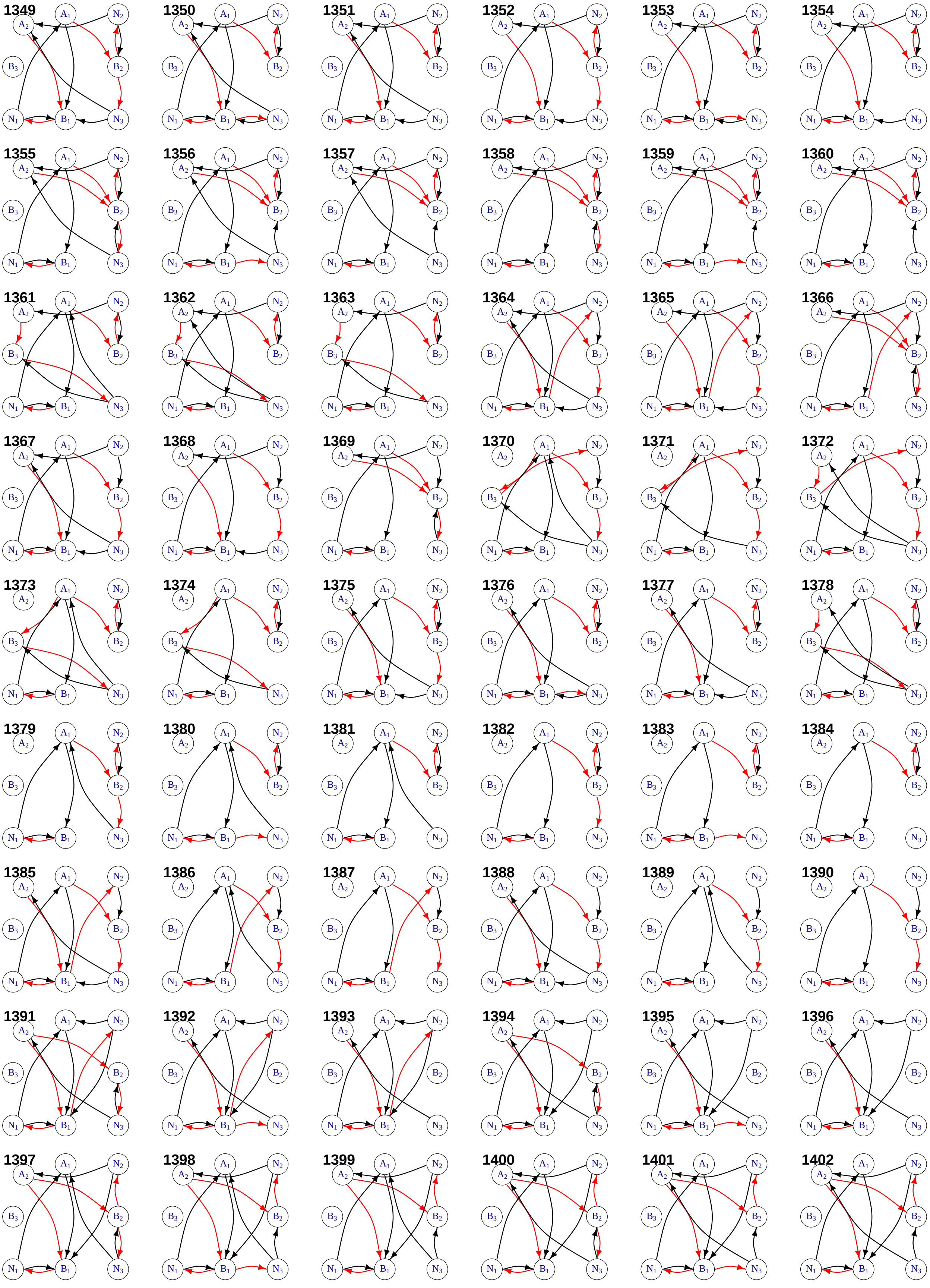
1349 1350 1351 1352 1353 1354
1355 1356 1357 1358 1359 1360
1361 1362 1363 1364 1365 1366
1367 1368 1369 1370 1371 1372
1373 1374 1375 1376 1377 1378
1379 1380 1381 1382 1383 1384
1385 1386 1387 1388 1389 1390
1391 1392 1393 1394 1395 1396
1397 1398 1399 1400 1401 1402

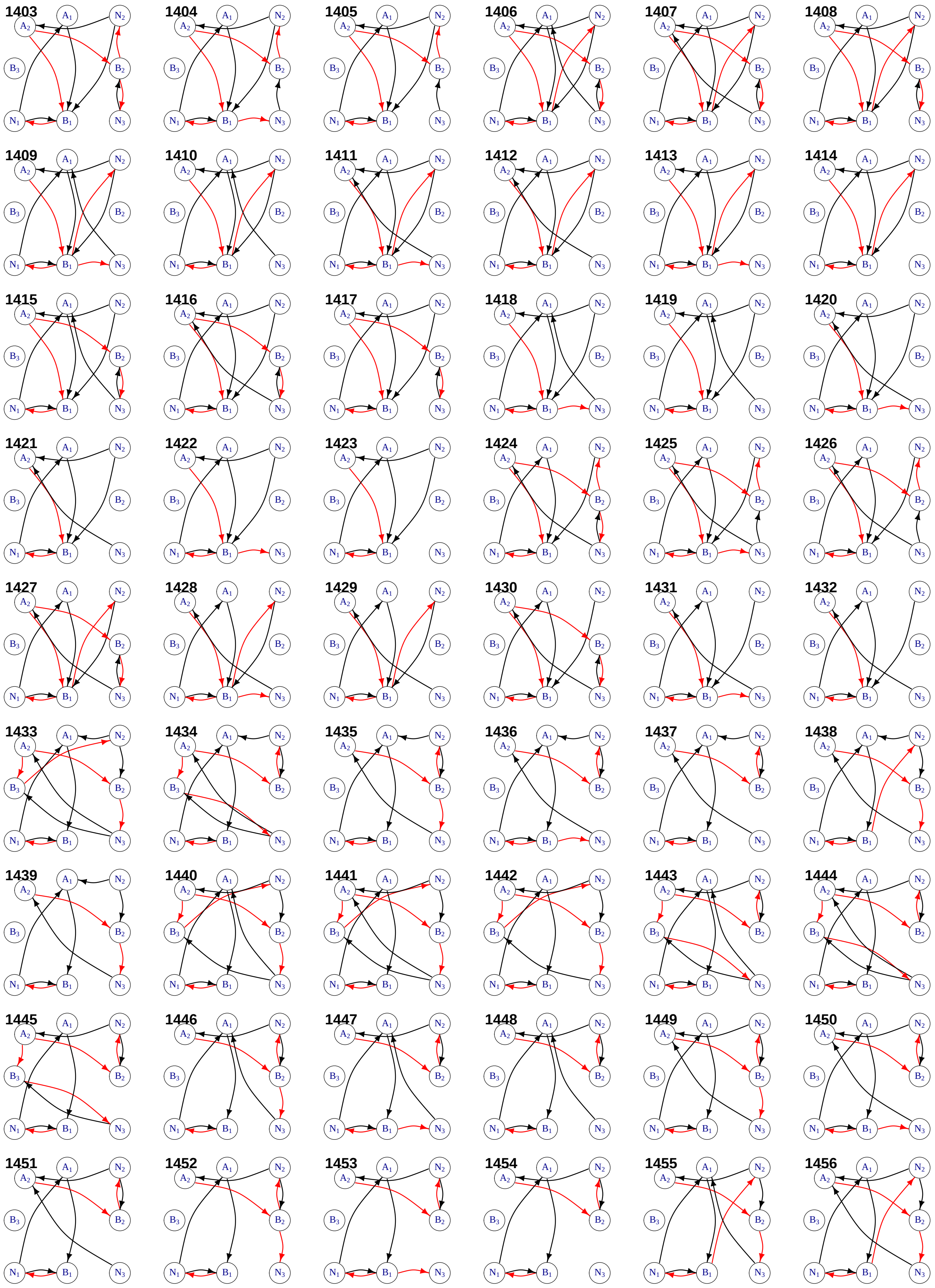
1403
1404
1405
1406
1407
1408
1409
1410
1411
1412
1413
1414
1415
1416
1417
1418
1419
1420
1421
1422
1423
1424
1425
1426
1427
1428
1429
1430
1431
1432
1433
1434
1435
1436
1437
1438
1439
1440
1441
1442
1443
1444
1445
1446
1447
1448
1449
1450
1451
1452
1453
1454
1455
1456

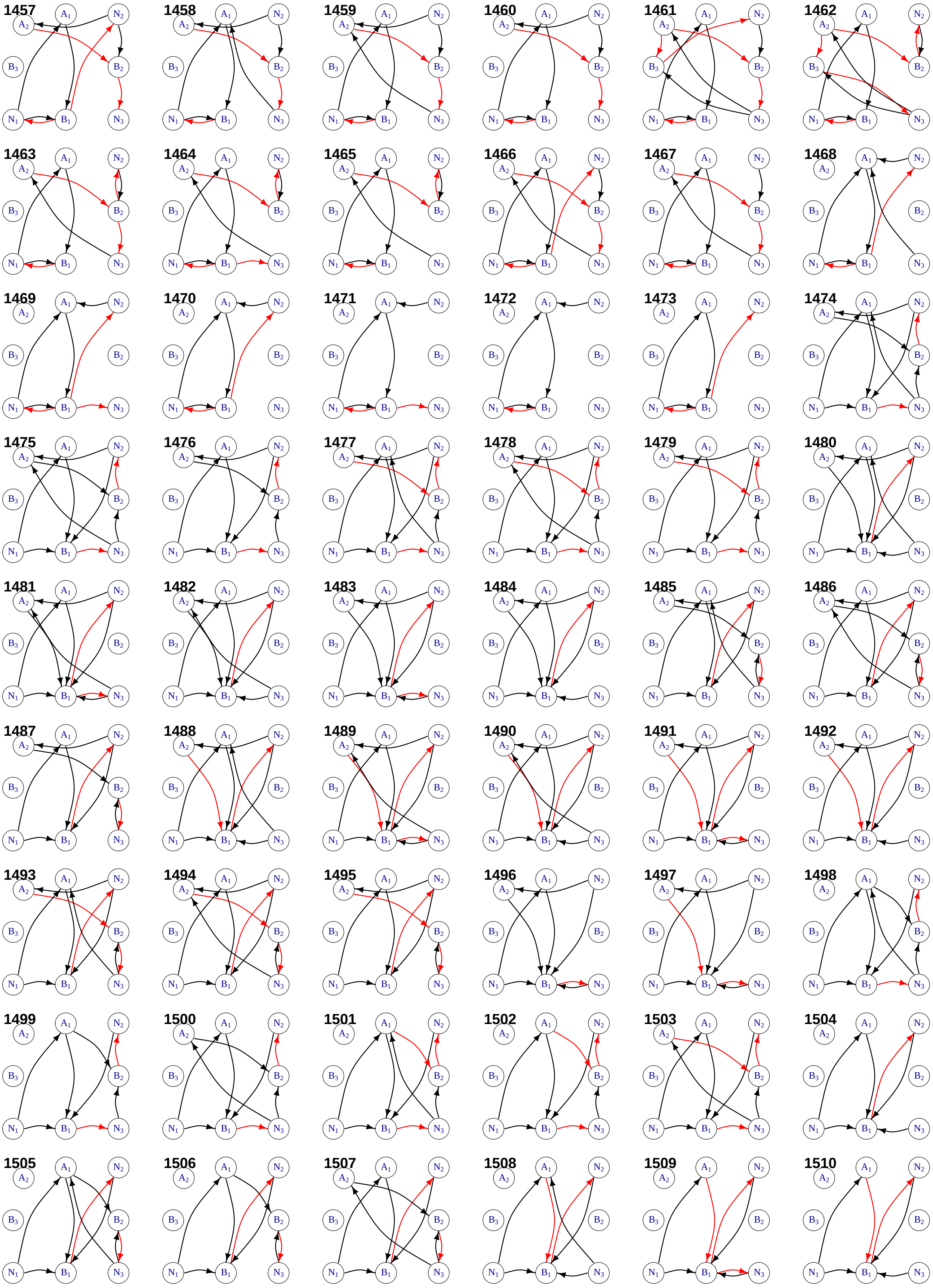
1457
1458
1459
1460
1461
1462
1463
1464
1465
1466
1467
1468
1469
1470
1471
1472
1473
1474
1475
1476
1477
1478
1479
1480
1481
1482
1483
1484
1485
1486
1487
1488
1489
1490
1491
1492
1493
1494
1495
1496
1497
1498
1499
1500
1501
1502
1503
1504
1505
1506
1507
1508
1509
1510

1511 1512 1513 1514 1515 1516

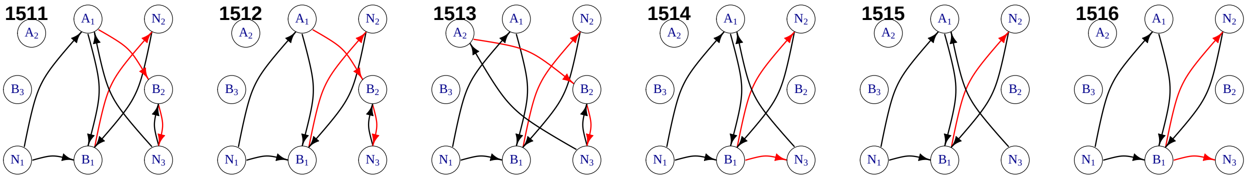

1517 1518 1519 1520 1521 1522

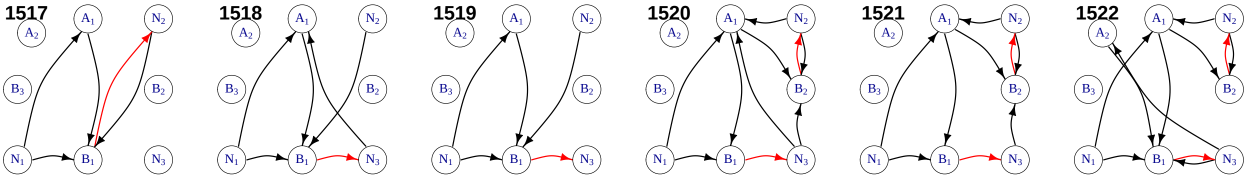

1523 1524 1525 1526 1527 1528

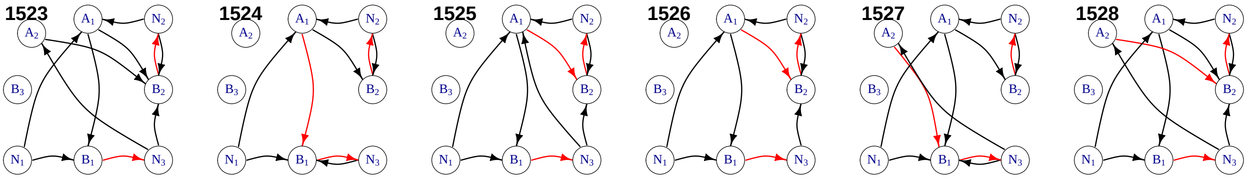

1529 1530 1531 1532 1533 1534

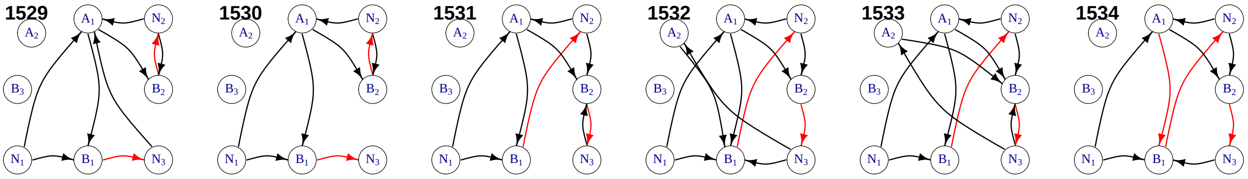

1535 1536 1537 1538 1539 1540

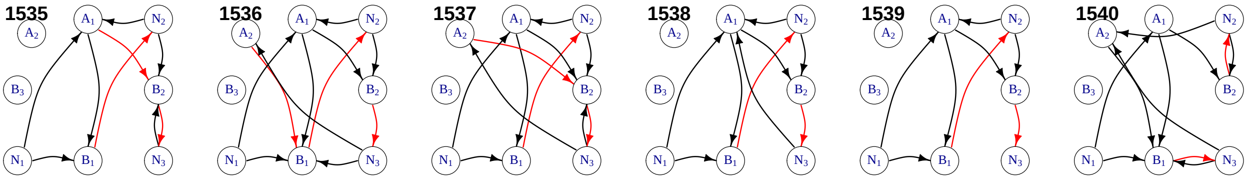

1541 1542 1543 1544 1545 1546

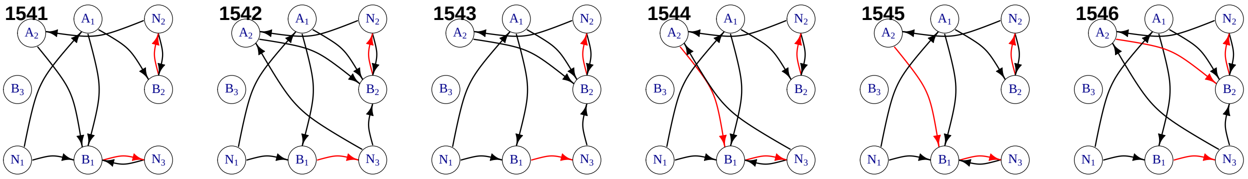

1547 1548 1549 1550 1551 1552

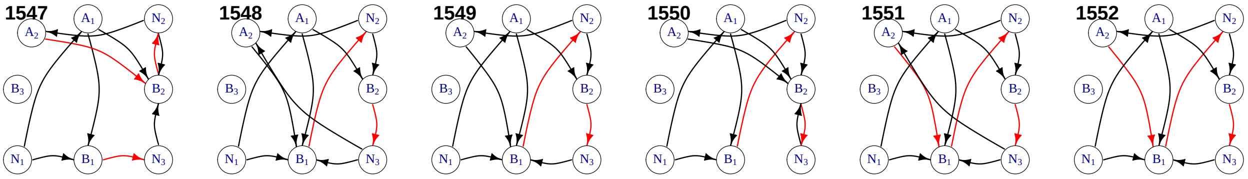

1553 1554 1555 1556 1557 1558

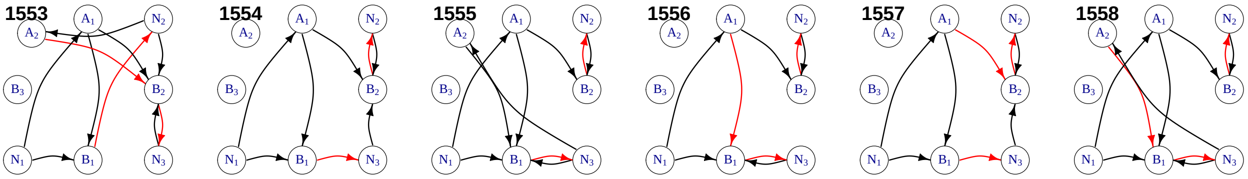

1559 1560 1561 1562 1563 1564

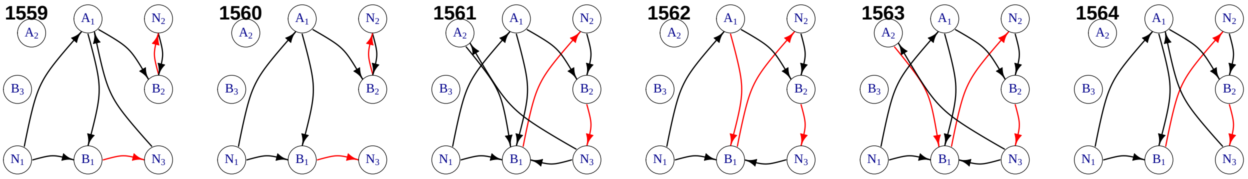

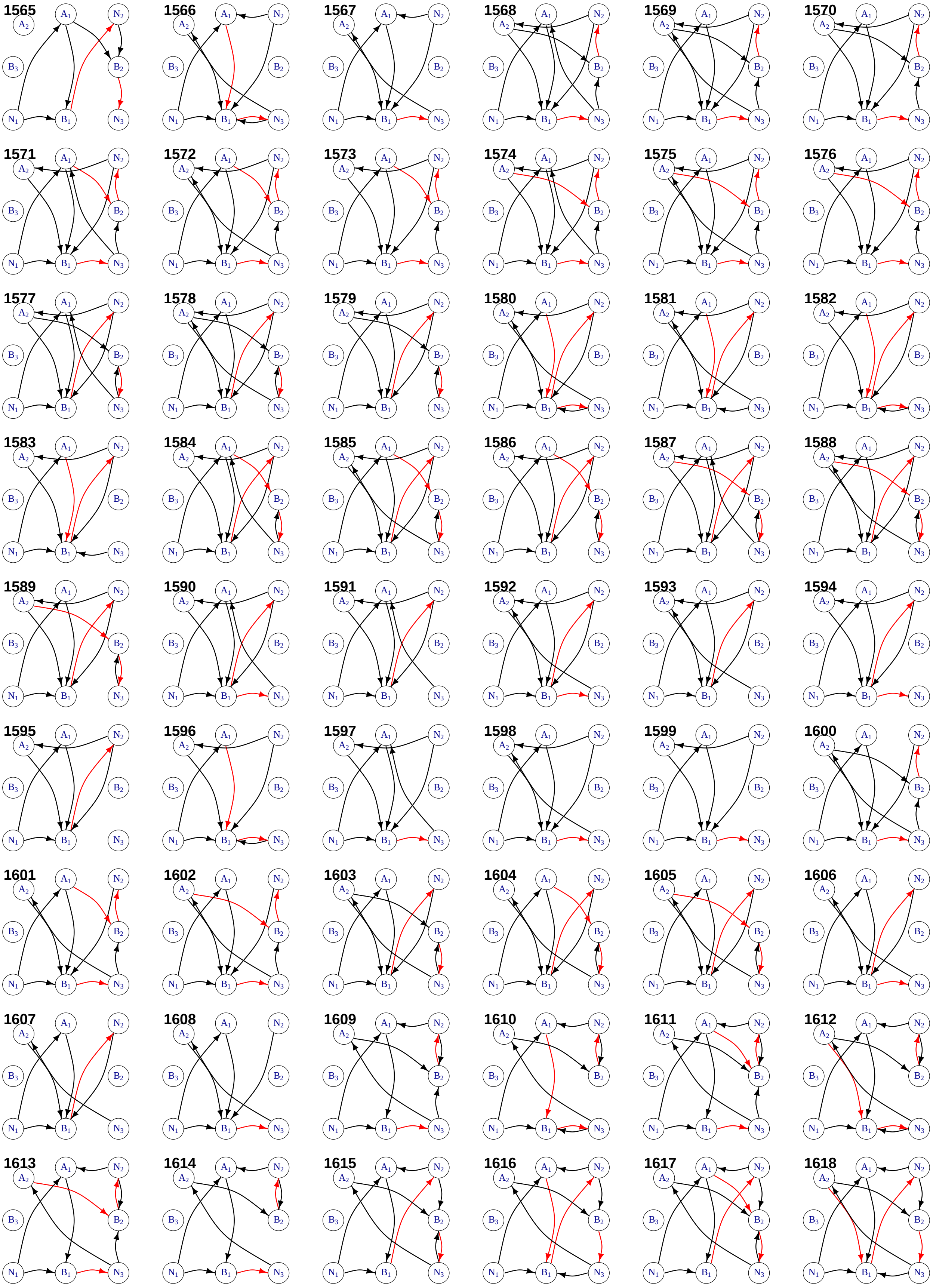
1565
1566
1567
1568
1569
1570
1571
1572
1573
1574
1575
1576
1577
1578
1579
1580
1581
1582
1583
1584
1585
1586
1587
1588
1589
1590
1591
1592
1593
1594
1595
1596
1597
1598
1599
1600
1601
1602
1603
1604
1605
1606
1607
1608
1609
1610
1611
1612
1613
1614
1615
1616
1617
1618

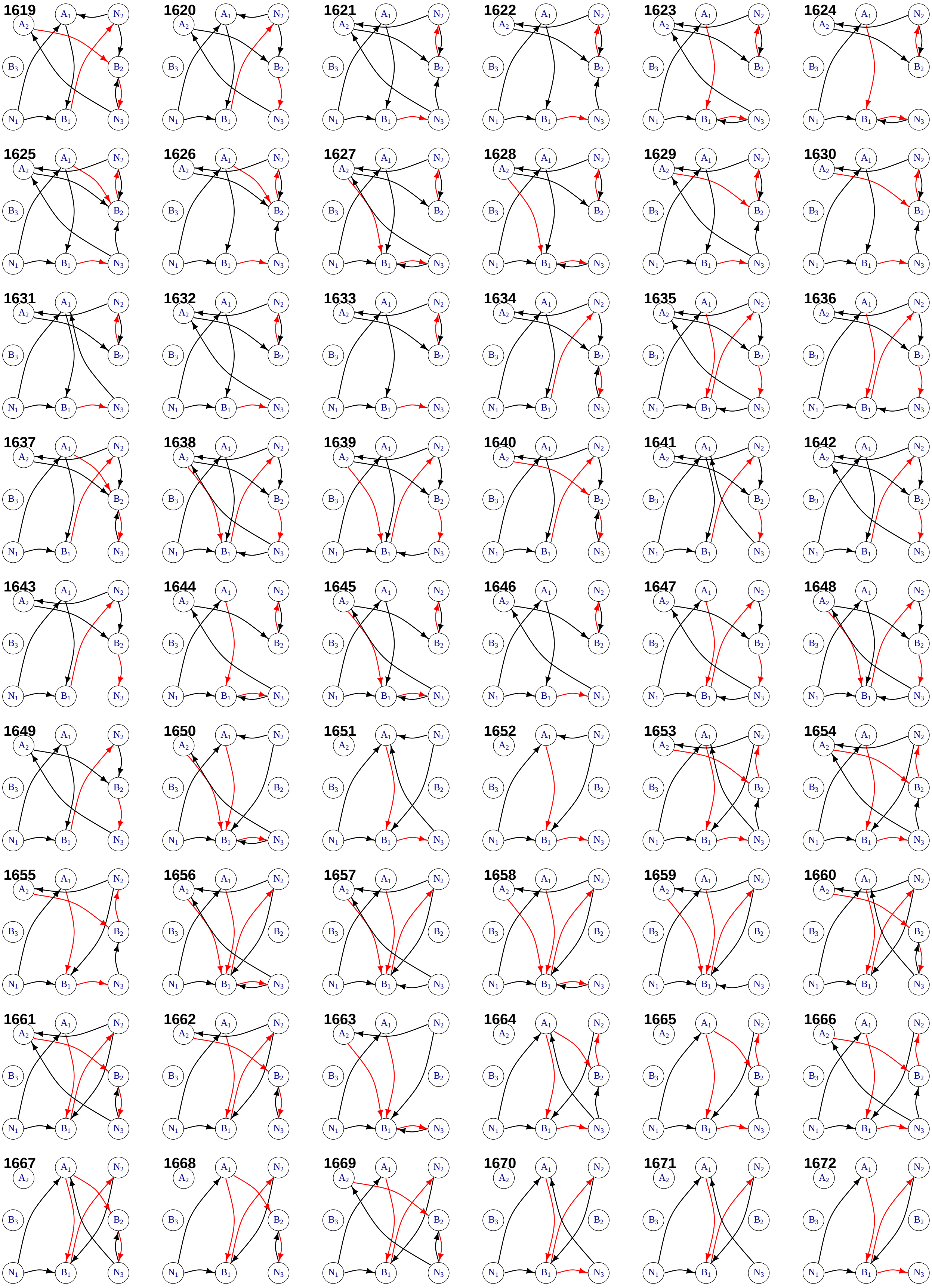

1619 1620 1621 1622 1623 1624

1625 1626 1627 1628 1629 1630

1631 1632 1633 1634 1635 1636

1637 1638 1639 1640 1641 1642

1643 1644 1645 1646 1647 1648

1649 1650 1651 1652 1653 1654

1655 1656 1657 1658 1659 1660

1661 1662 1663 1664 1665 1666

1667 1668 1669 1670 1671 1672

1673 1674 1675 1676 1677 1678

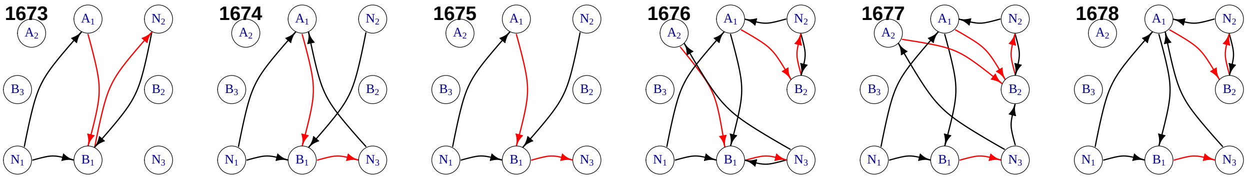

1679 1680 1681 1682 1683 1684

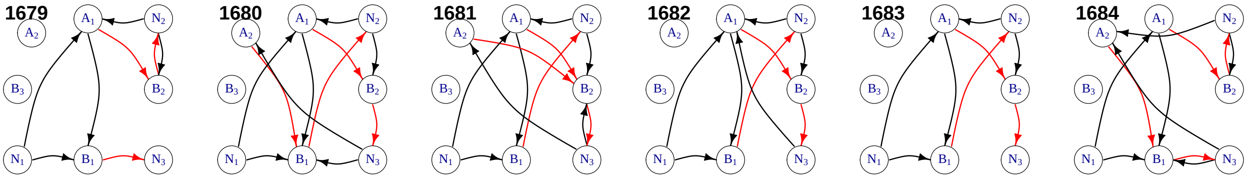

1685 1686 1687 1688 1689 1690

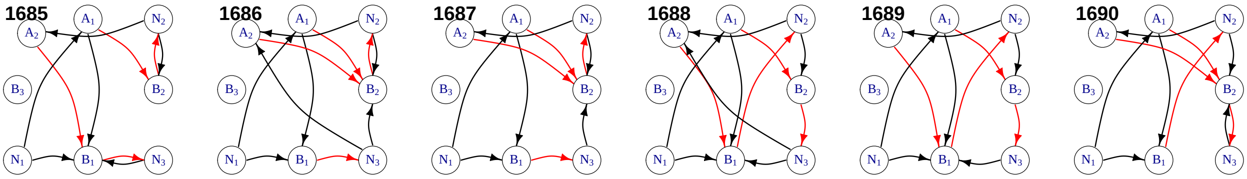

1691 1692 1693 1694 1695 1696

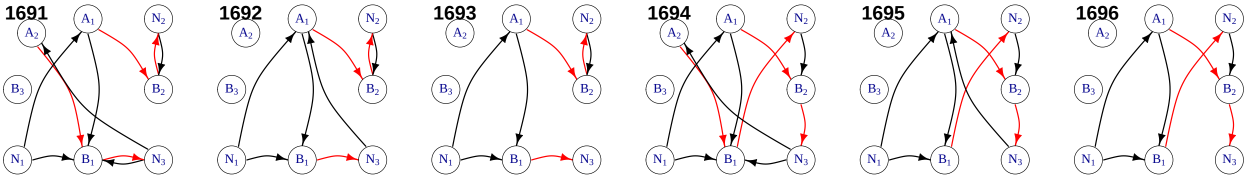

1697 1698 1699 1700 1701 1702

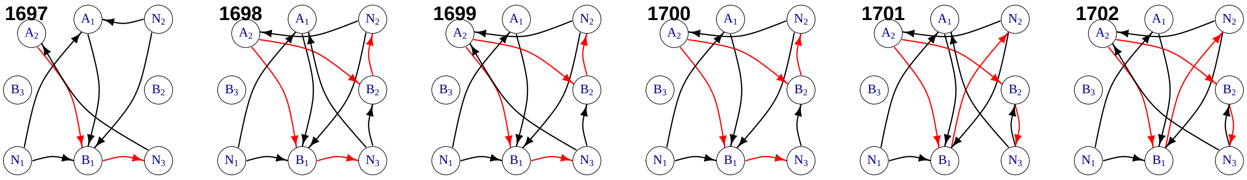

1703 1704 1705 1706 1707 1708

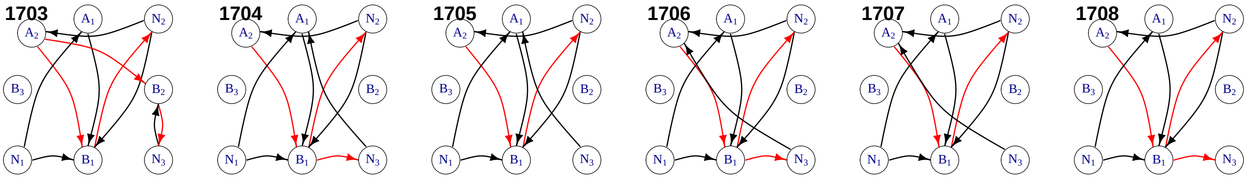

1709 1710 1711 1712 1713 1714

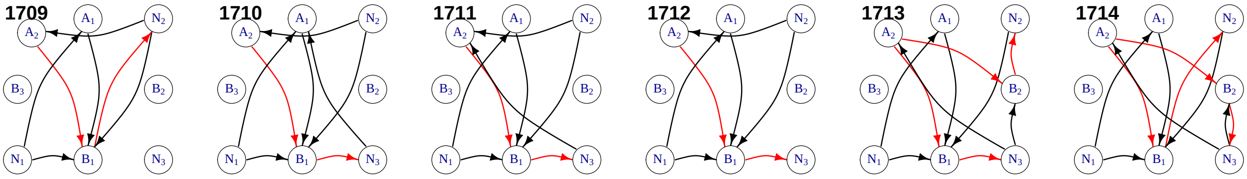

1715 1716 1717 1718 1719 1720

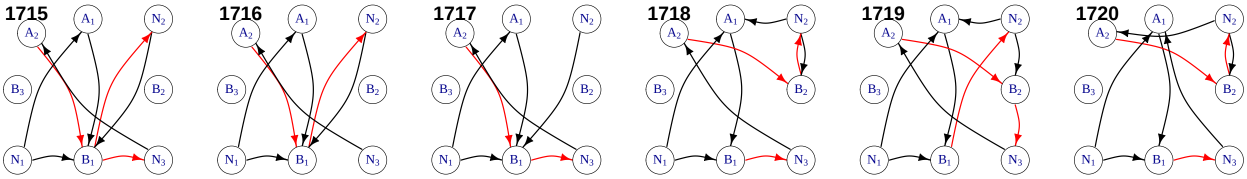

1721 1722 1723 1724 1725 1726

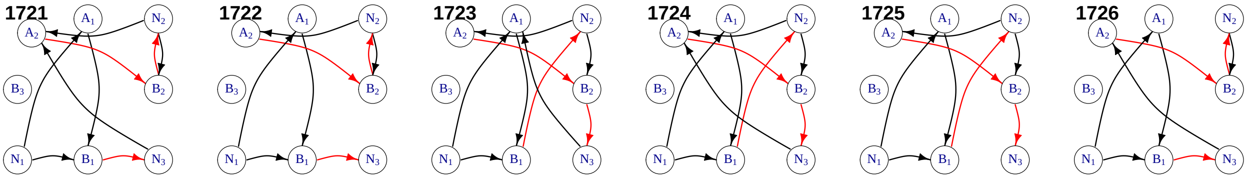

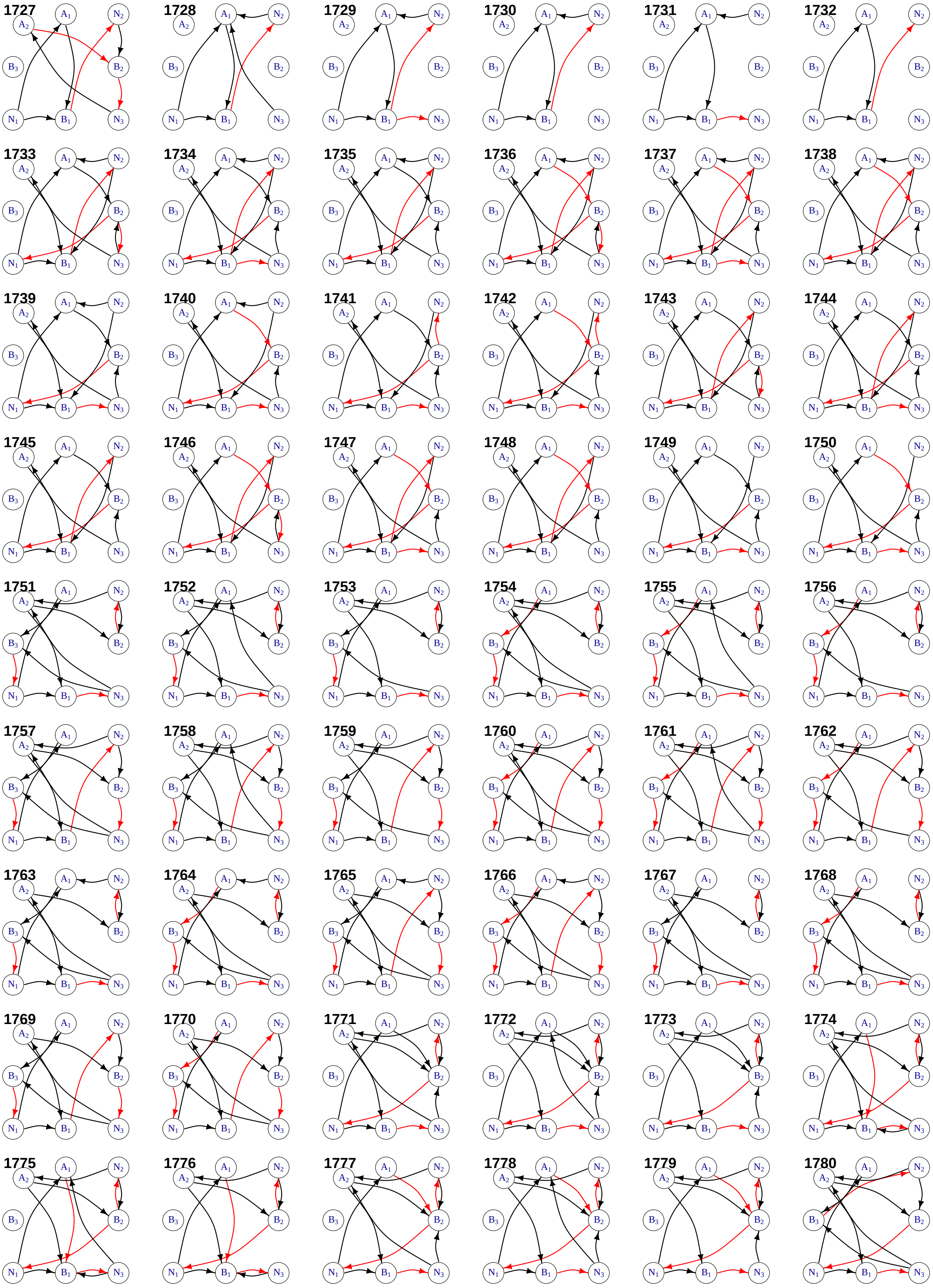
1727
1728
1729
1730
1731
1732
1733
1734
1735
1736
1737
1738
1739
1740
1741
1742
1743
1744
1745
1746
1747
1748
1749
1750
1751
1752
1753
1754
1755
1756
1757
1758
1759
1760
1761
1762
1763
1764
1765
1766
1767
1768
1769
1770
1771
1772
1773
1774
1775
1776
1777
1778
1779
1780

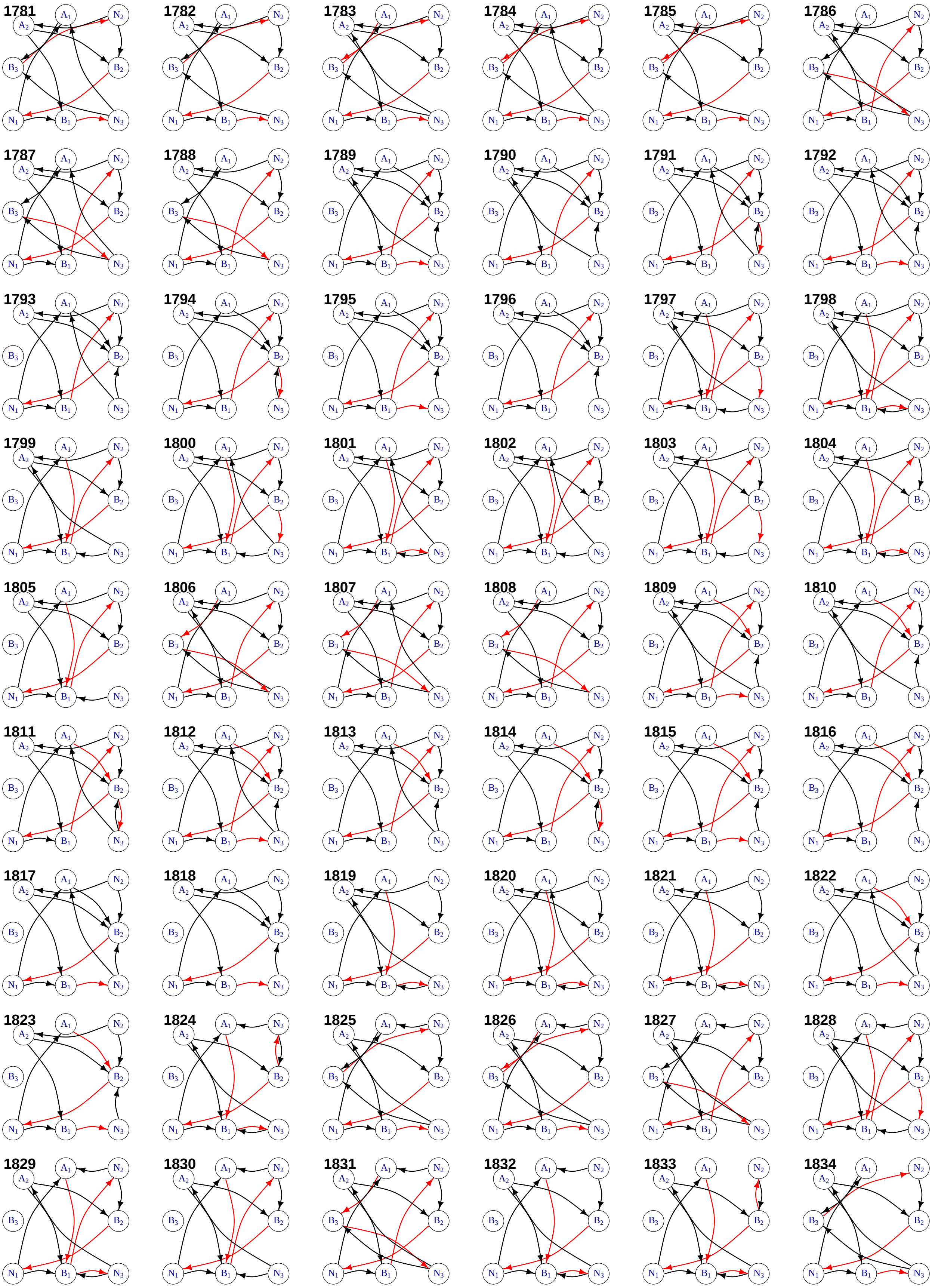
1781
1782
1783
1784
1785
1786
1787
1788
1789
1790
1791
1792
1793
1794
1795
1796
1797
1798
1799
1800
1801
1802
1803
1804
1805
1806
1807
1808
1809
1810
1811
1812
1813
1814
1815
1816
1817
1818
1819
1820
1821
1822
1823
1824
1825
1826
1827
1828
1829
1830
1831
1832
1833
1834

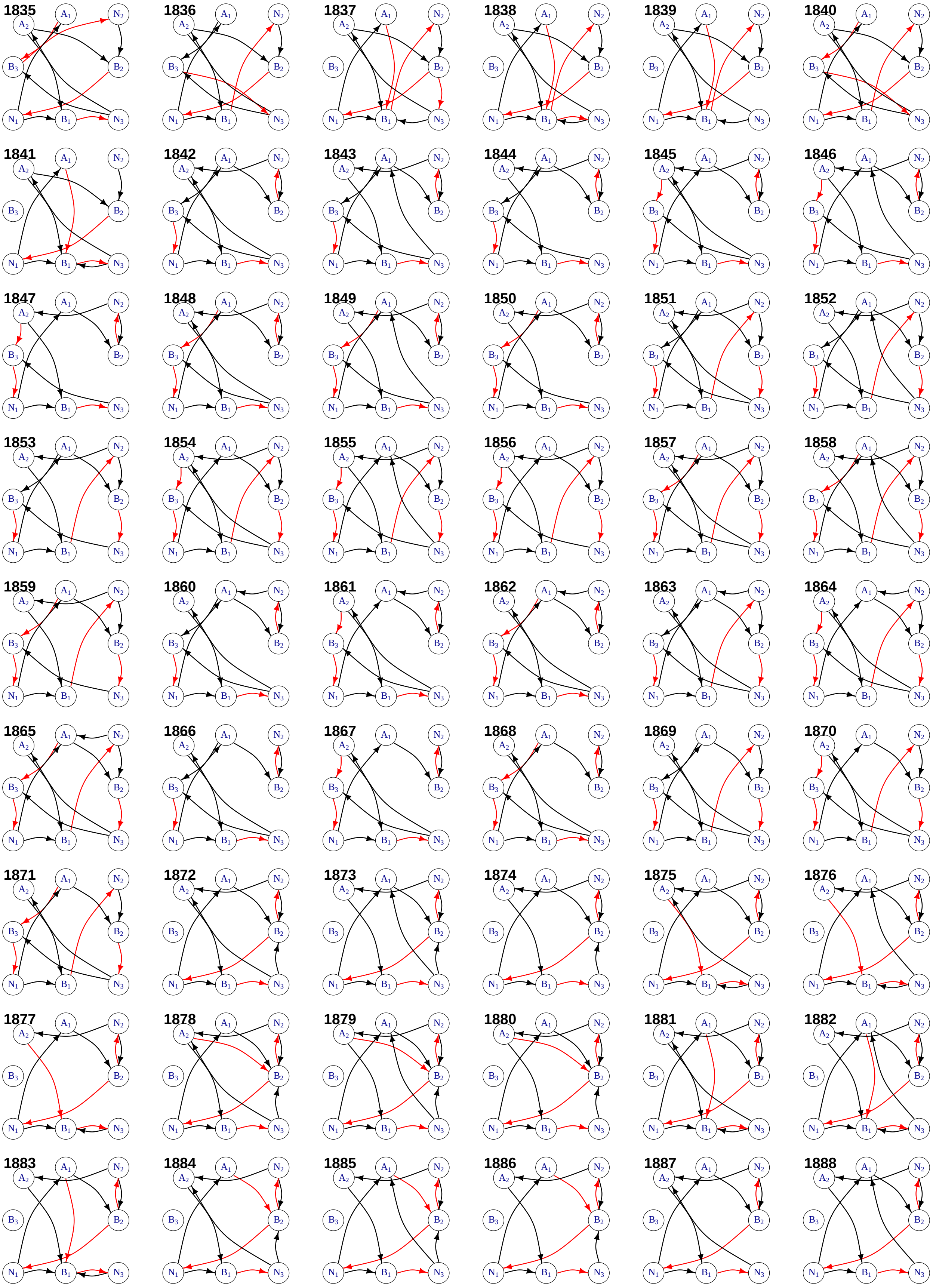
1835
1836
1837
1838
1839
1840
1841
1842
1843
1844
1845
1846
1847
1848
1849
1850
1851
1852
1853
1854
1855
1856
1857
1858
1859
1860
1861
1862
1863
1864
1865
1866
1867
1868
1869
1870
1871
1872
1873
1874
1875
1876
1877
1878
1879
1880
1881
1882
1883
1884
1885
1886
1887
1888

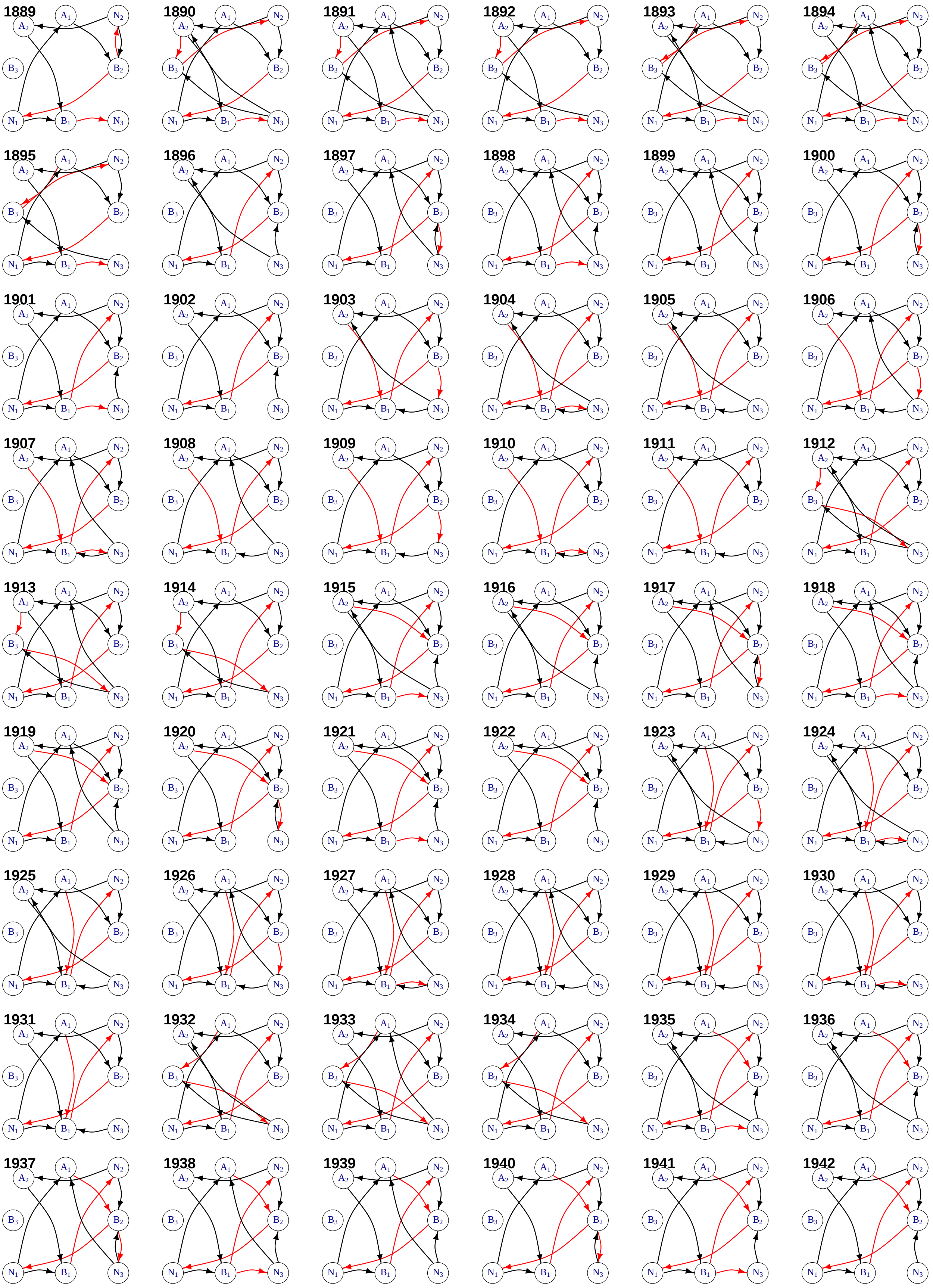
1889
1890
1891
1892
1893
1894
1895
1896
1897
1898
1899
1900
1901
1902
1903
1904
1905
1906
1907
1908
1909
1910
1911
1912
1913
1914
1915
1916
1917
1918
1919
1920
1921
1922
1923
1924
1925
1926
1927
1928
1929
1930
1931
1932
1933
1934
1935
1936
1937
1938
1939
1940
1941
1942

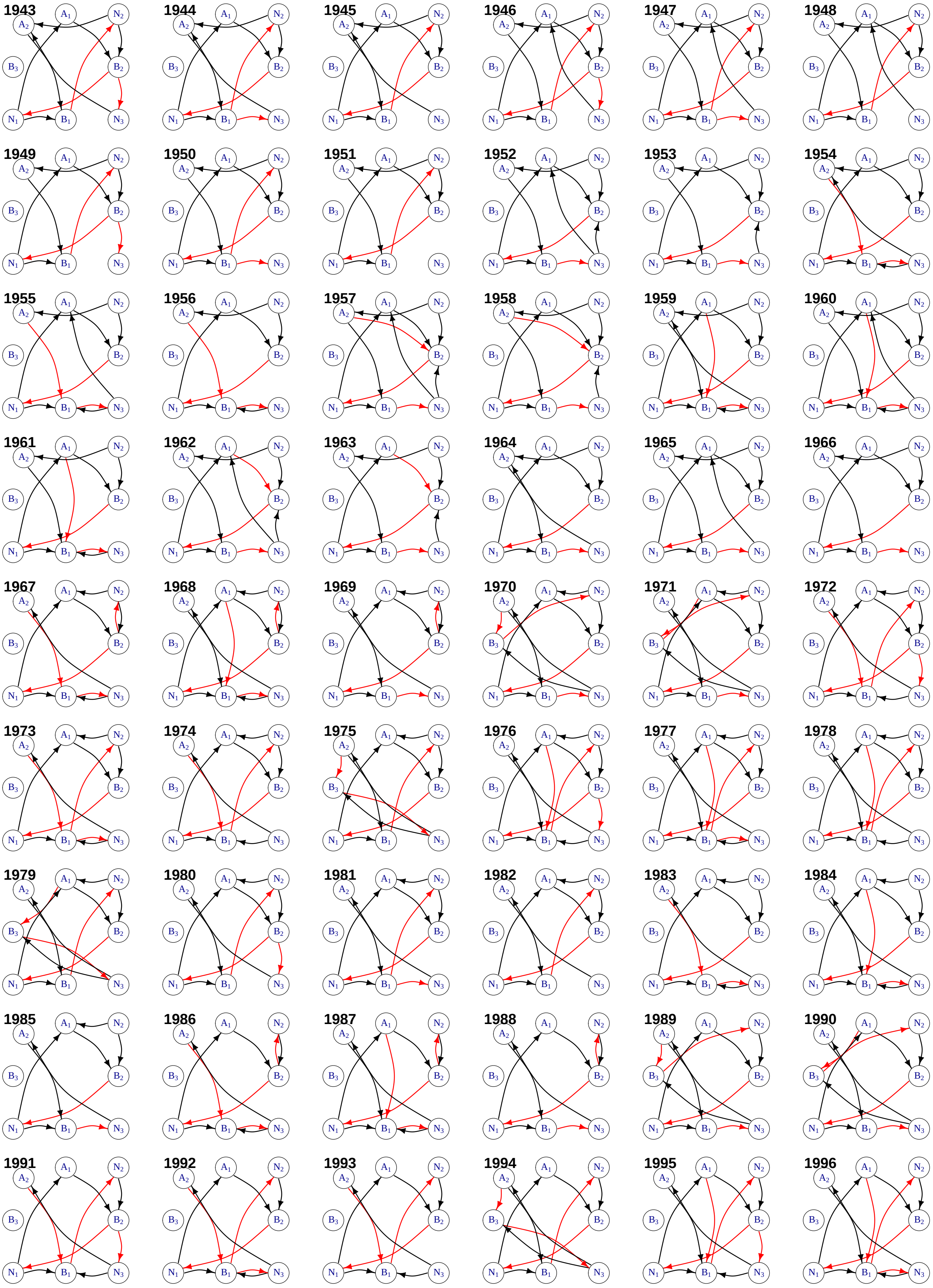
1943
1944
1945
1946
1947
1948
1949
1950
1951
1952
1953
1954
1955
1956
1957
1958
1959
1960
1961
1962
1963
1964
1965
1966
1967
1968
1969
1970
1971
1972
1973
1974
1975
1976
1977
1978
1979
1980
1981
1982
1983
1984
1985
1986
1987
1988
1989
1990
1991
1992
1993
1994
1995
1996

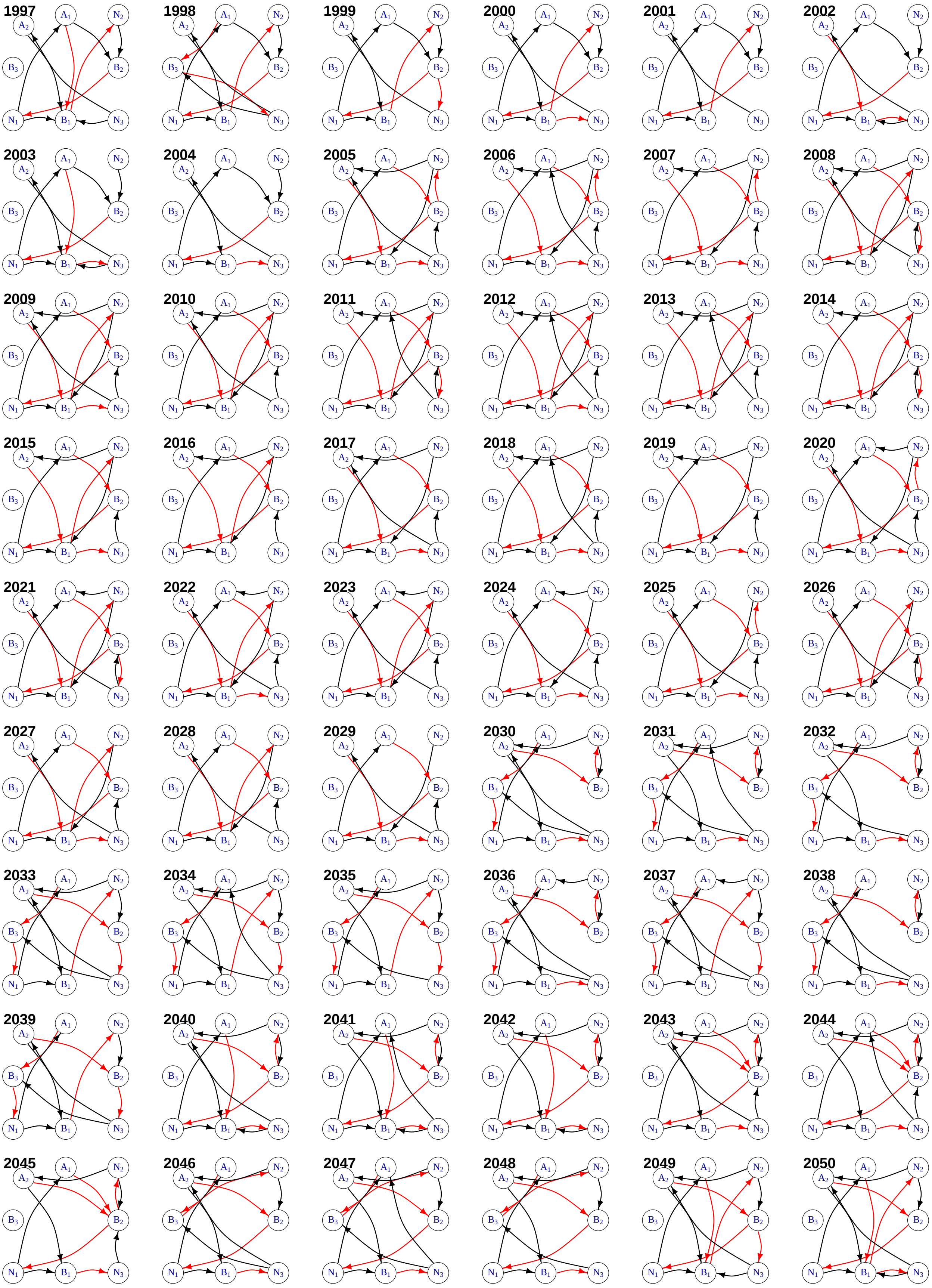
1997
1998
1999
2000
2001
2002
2003
2004
2005
2006
2007
2008
2009
2010
2011
2012
2013
2014
2015
2016
2017
2018
2019
2020
2021
2022
2023
2024
2025
2026
2027
2028
2029
2030
2031
2032
2033
2034
2035
2036
2037
2038
2039
2040
2041
2042
2043
2044
2045
2046
2047
2048
2049
2050

2051 2052 2053 2054 2055 2056

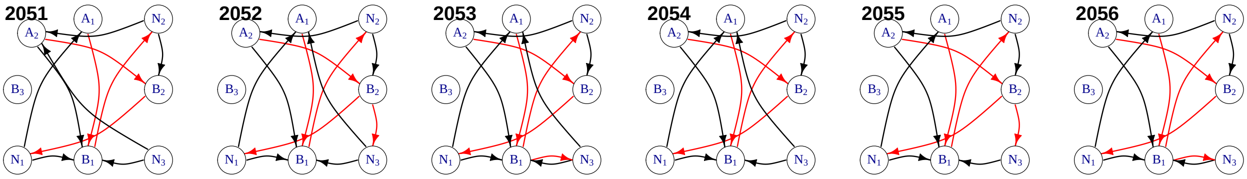

2057 2058 2059 2060 2061 2062

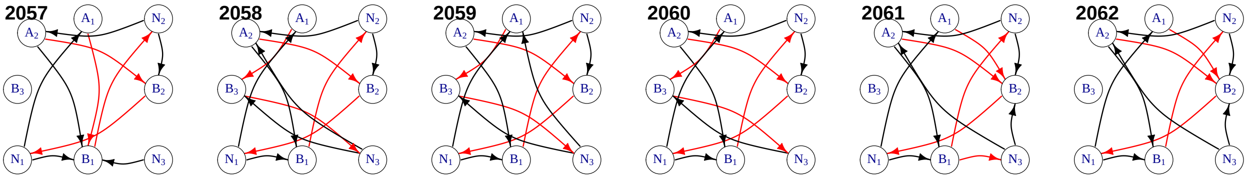

2063 2064 2065 2066 2067 2068

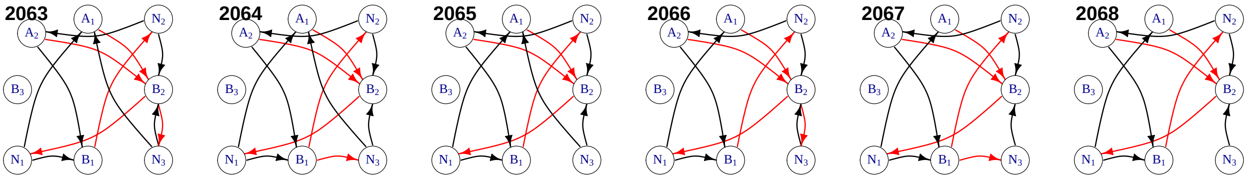

2069 2070 2071 2072 2073 2074

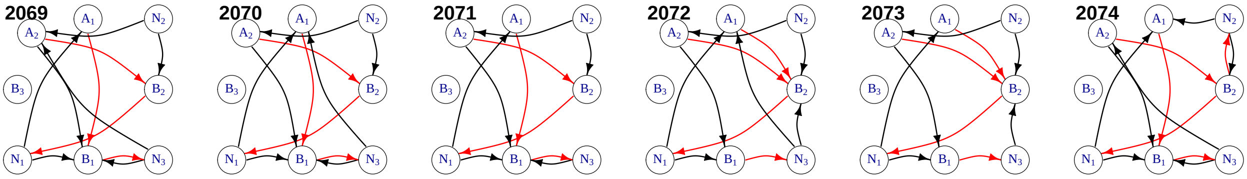

2075 2076 2077 2078 2079 2080

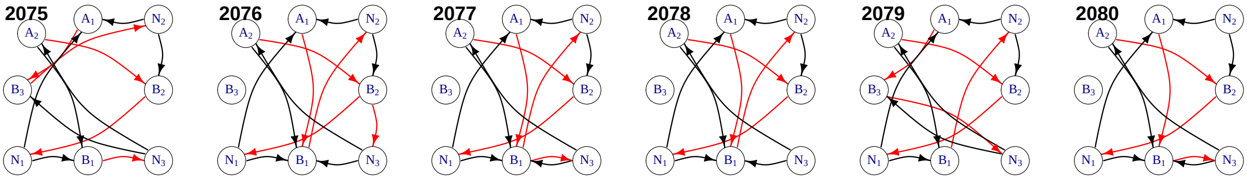

2081 2082 2083 2084 2085 2086

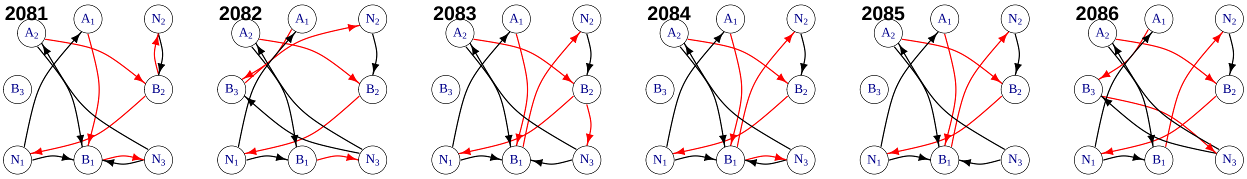

2087 2088 2089 2090 2091 2092

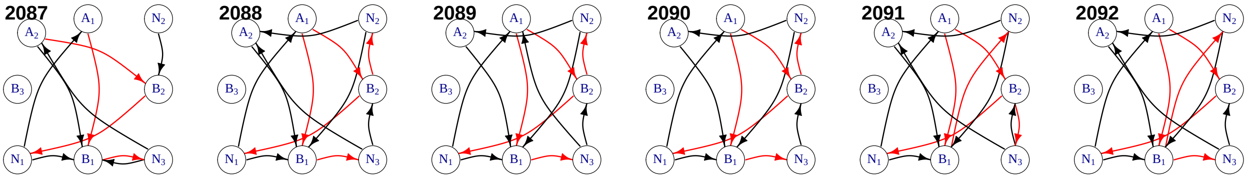

2093 2094 2095 2096 2097 2098

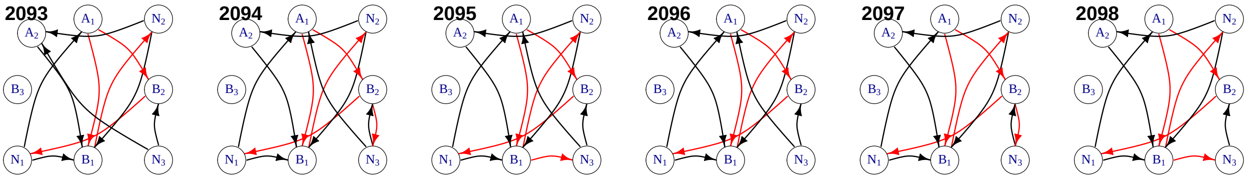

2099 2100 2101 2102 2103 2104

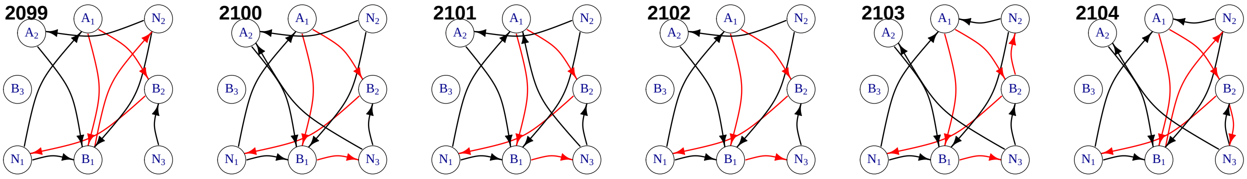

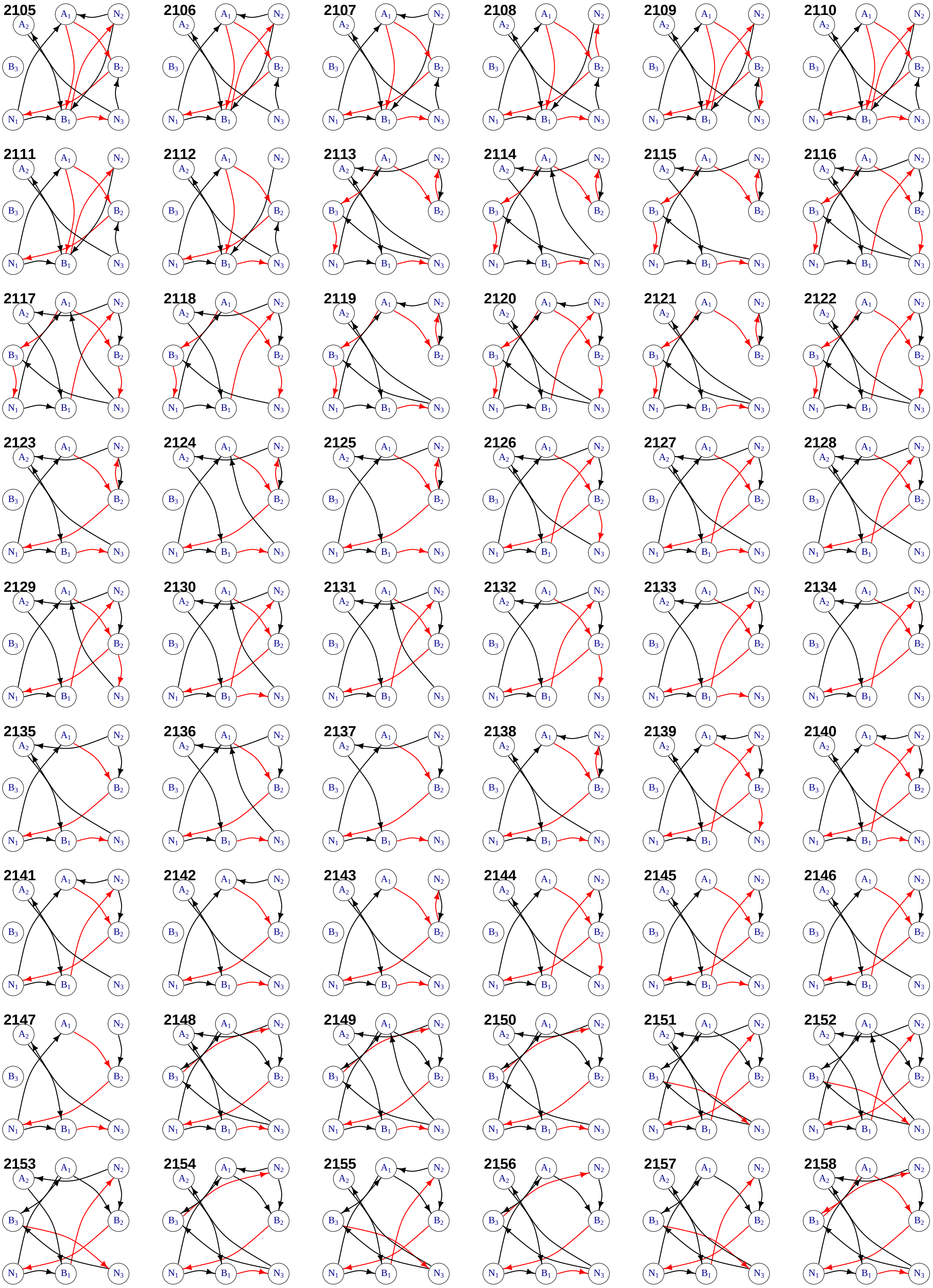
2105 2106 2107 2108 2109 2110
2111 2112 2113 2114 2115 2116
2117 2118 2119 2120 2121 2122
2123 2124 2125 2126 2127 2128
2129 2130 2131 2132 2133 2134
2135 2136 2137 2138 2139 2140
2141 2142 2143 2144 2145 2146
2147 2148 2149 2150 2151 2152
2153 2154 2155 2156 2157 2158

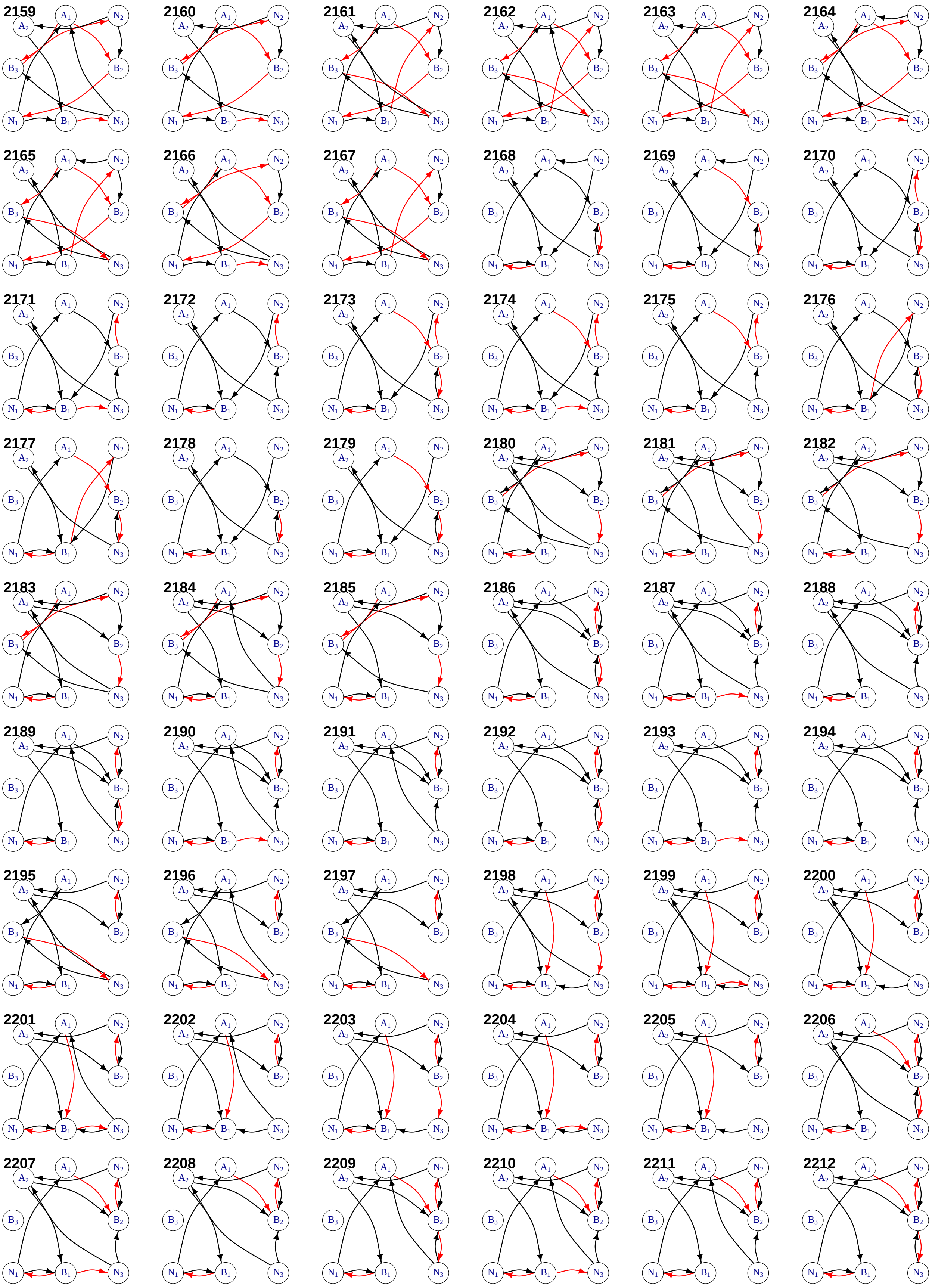
2159
2160
2161
2162
2163
2164
2165
2166
2167
2168
2169
2170
2171
2172
2173
2174
2175
2176
2177
2178
2179
2180
2181
2182
2183
2184
2185
2186
2187
2188
2189
2190
2191
2192
2193
2194
2195
2196
2197
2198
2199
2200
2201
2202
2203
2204
2205
2206
2207
2208
2209
2210
2211
2212

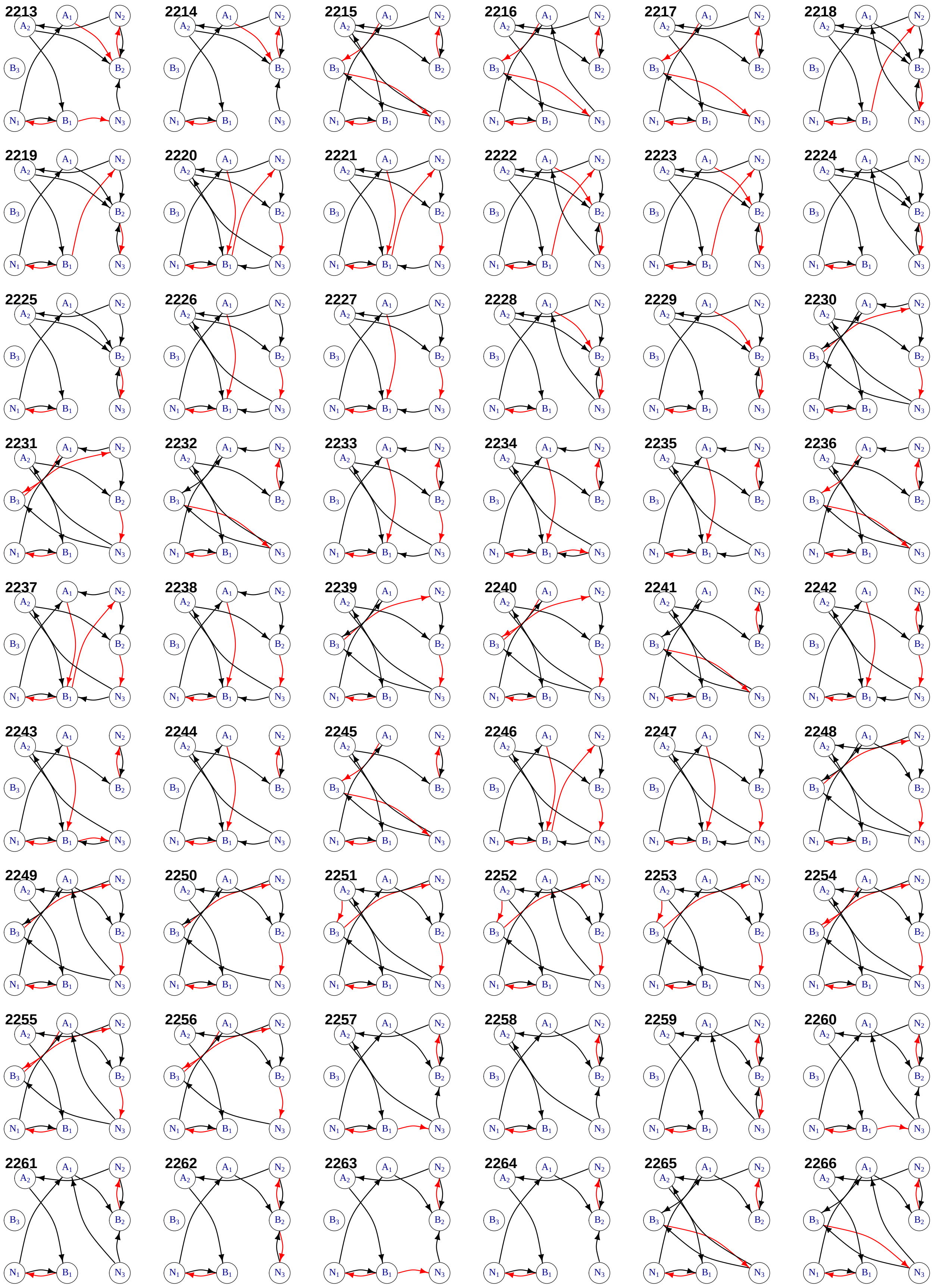
2213
2214
2215
2216
2217
2218
2219
2220
2221
2222
2223
2224
2225
2226
2227
2228
2229
2230
2231
2232
2233
2234
2235
2236
2237
2238
2239
2240
2241
2242
2243
2244
2245
2246
2247
2248
2249
2250
2251
2252
2253
2254
2255
2256
2257
2258
2259
2260
2261
2262
2263
2264
2265
2266

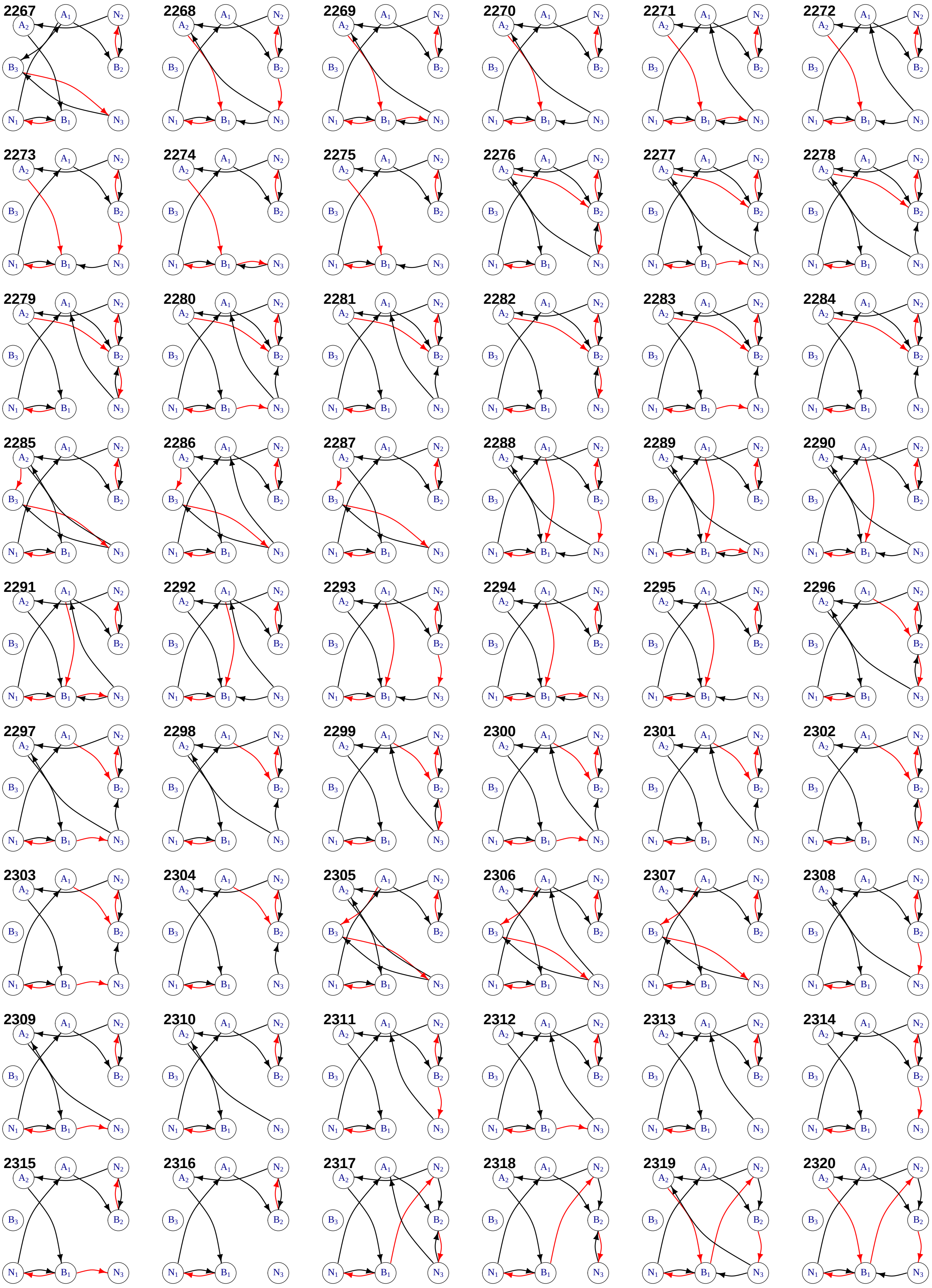
2267
2268
2269
2270
2271
2272
2273
2274
2275
2276
2277
2278
2279
2280
2281
2282
2283
2284
2285
2286
2287
2288
2289
2290
2291
2292
2293
2294
2295
2296
2297
2298
2299
2300
2301
2302
2303
2304
2305
2306
2307
2308
2309
2310
2311
2312
2313
2314
2315
2316
2317
2318
2319
2320

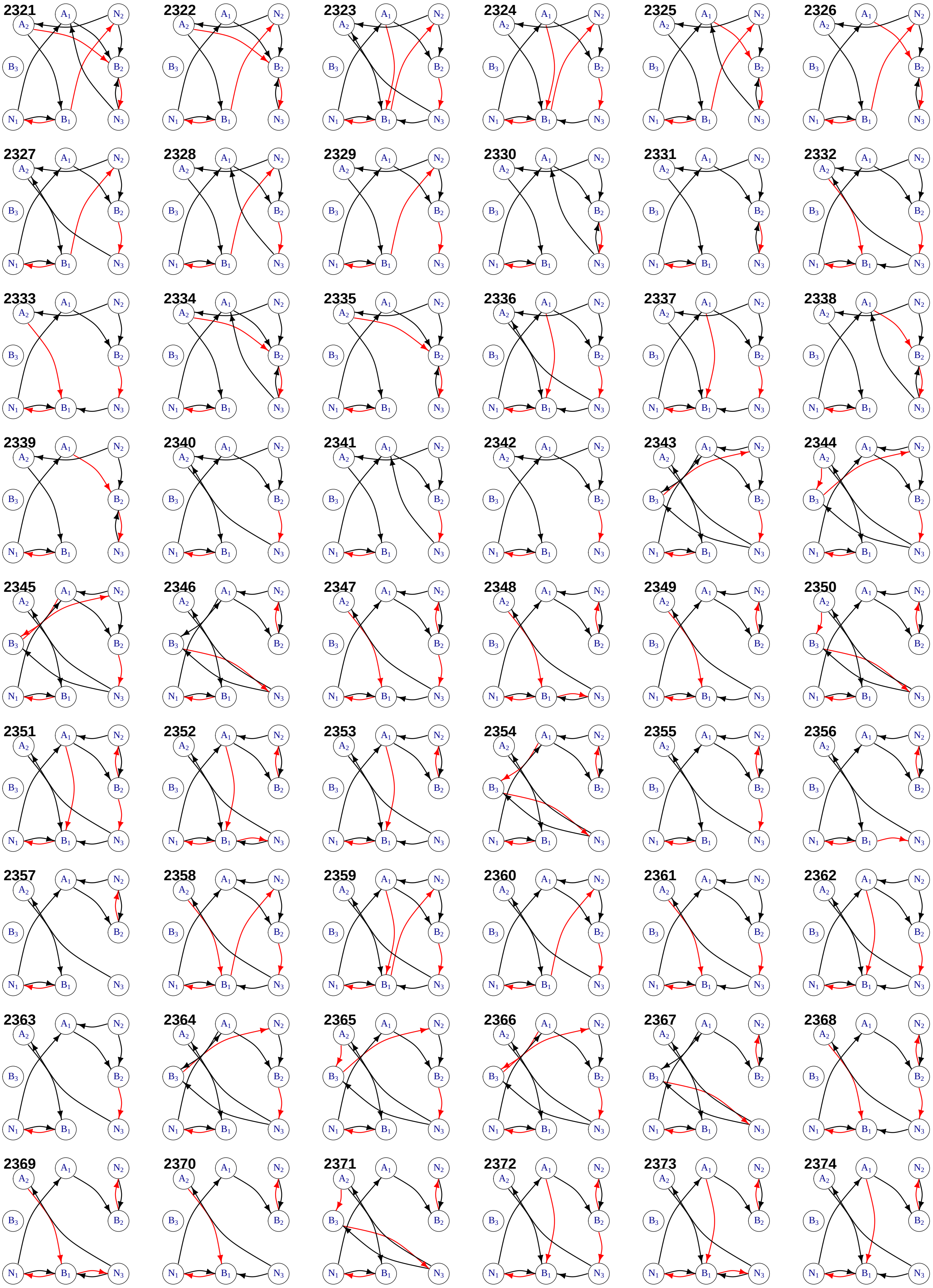

2321 2322 2323 2324 2325 2326
2327 2328 2329 2330 2331 2332
2333 2334 2335 2336 2337 2338
2339 2340 2341 2342 2343 2344
2345 2346 2347 2348 2349 2350
2351 2352 2353 2354 2355 2356
2357 2358 2359 2360 2361 2362
2363 2364 2365 2366 2367 2368
2369 2370 2371 2372 2373 2374

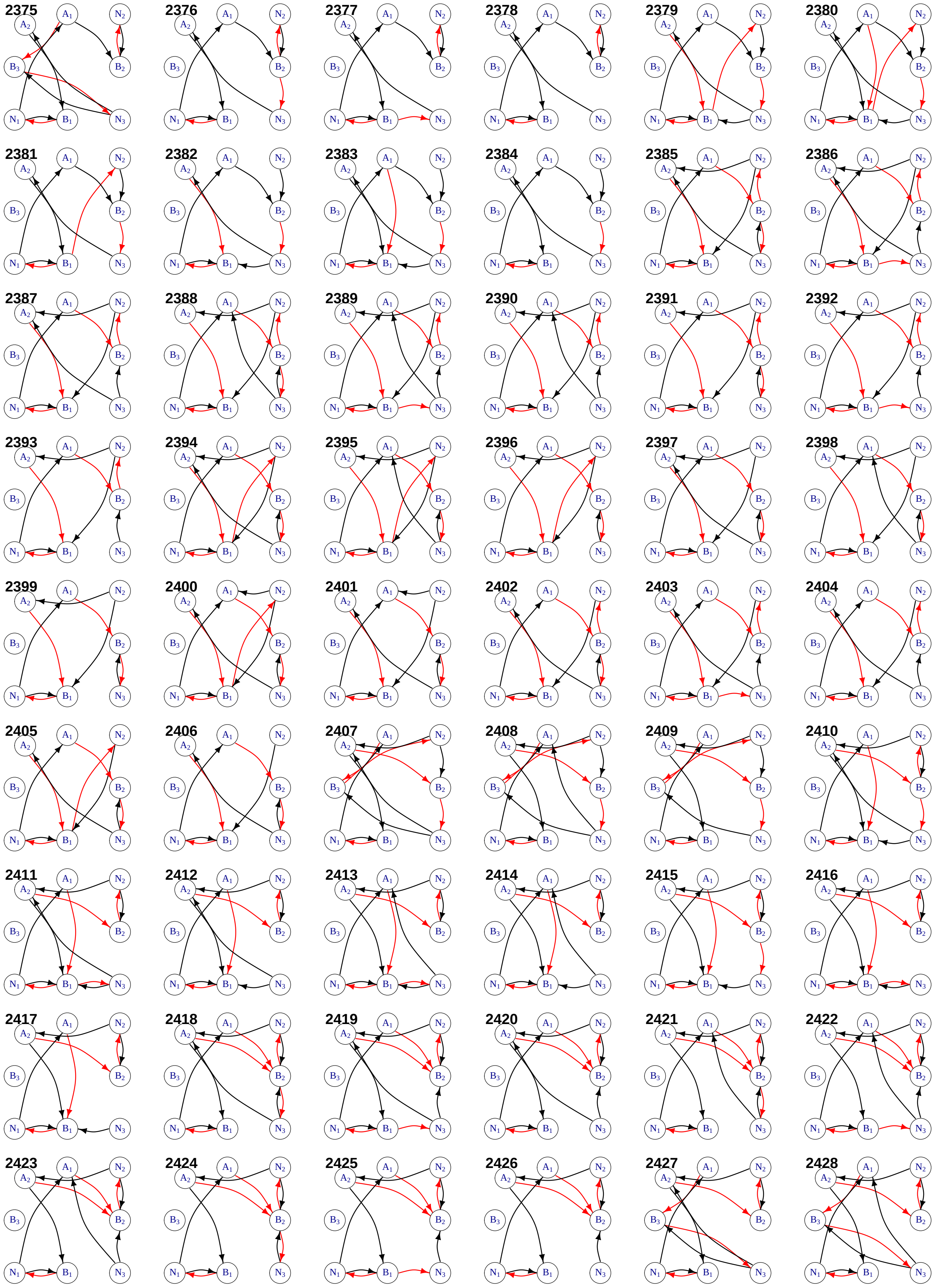
2375
2376
2377
2378
2379
2380
2381
2382
2383
2384
2385
2386
2387
2388
2389
2390
2391
2392
2393
2394
2395
2396
2397
2398
2399
2400
2401
2402
2403
2404
2405
2406
2407
2408
2409
2410
2411
2412
2413
2414
2415
2416
2417
2418
2419
2420
2421
2422
2423
2424
2425
2426
2427
2428

2429 2430 2431 2432 2433 2434

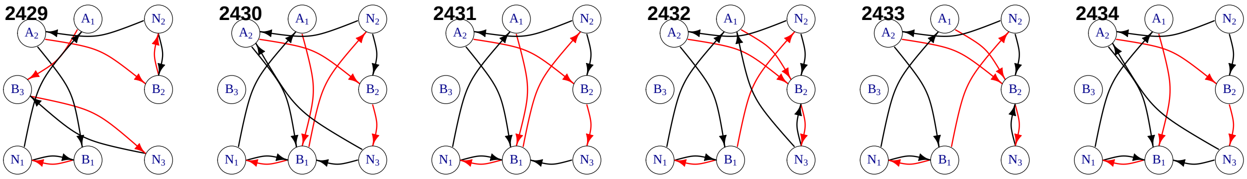

2435 2436 2437 2438 2439 2440

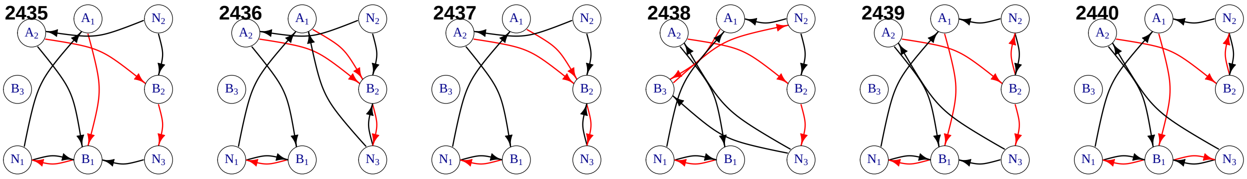

2441 2442 2443 2444 2445 2446

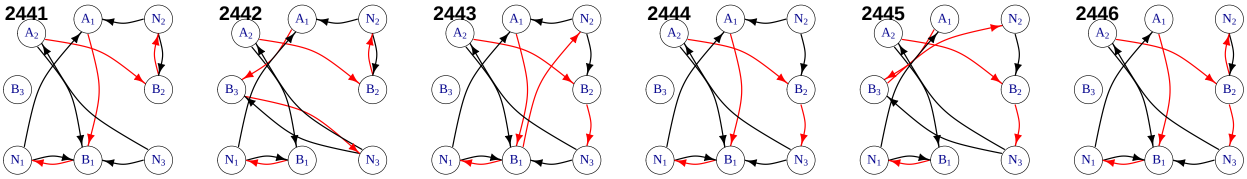

2447 2448 2449 2450 2451 2452

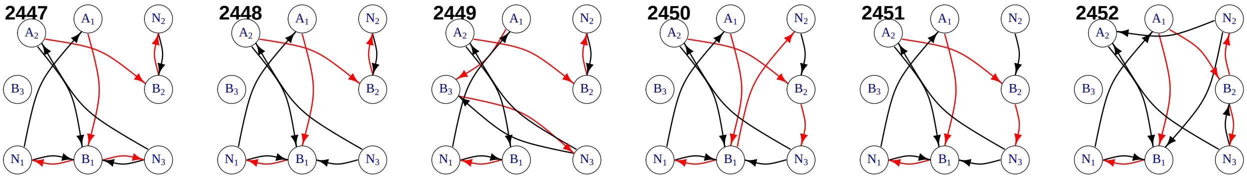

2453 2454 2455 2456 2457 2458

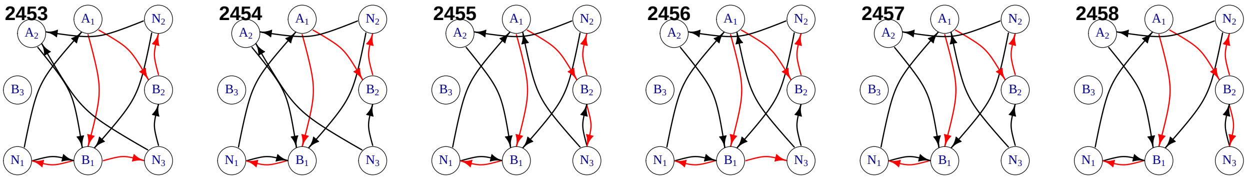

2459 2460 2461 2462 2463 2464

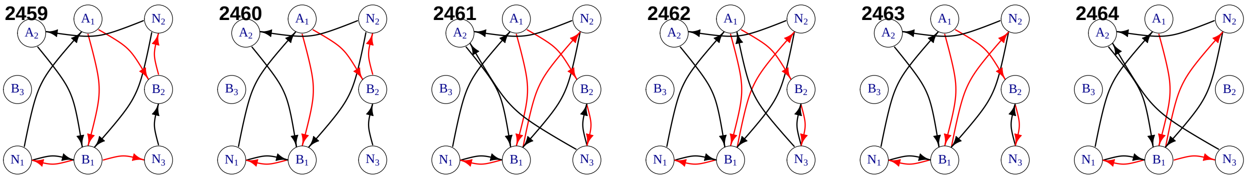

2465 2466 2467 2468 2469 2470

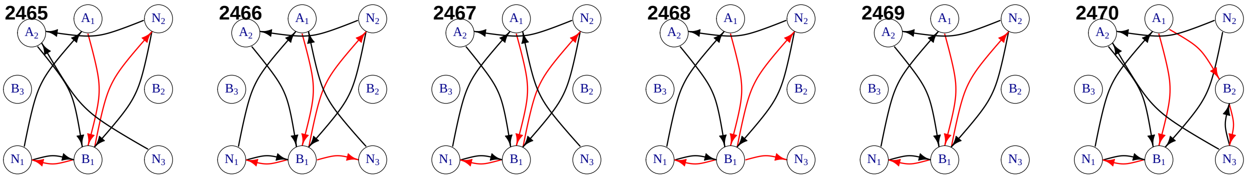

2471 2472 2473 2474 2475 2476

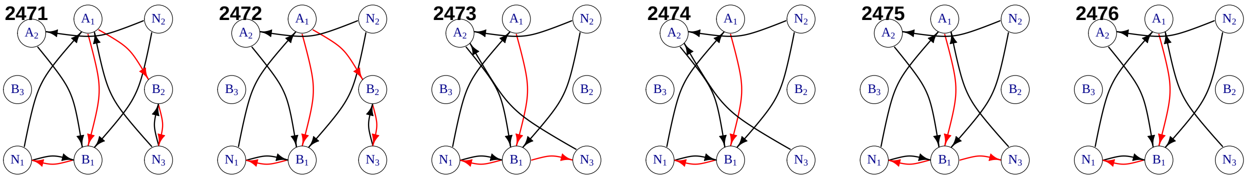

2477 2478 2479 2480 2481 2482

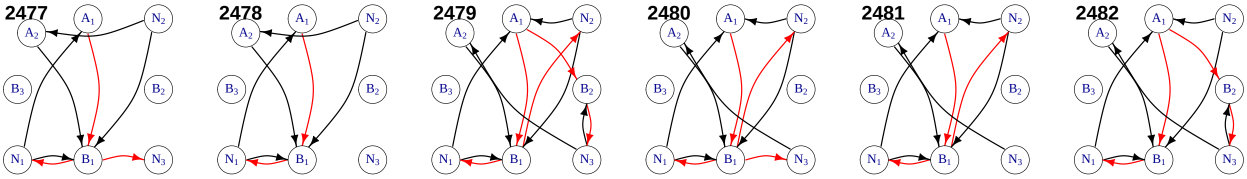

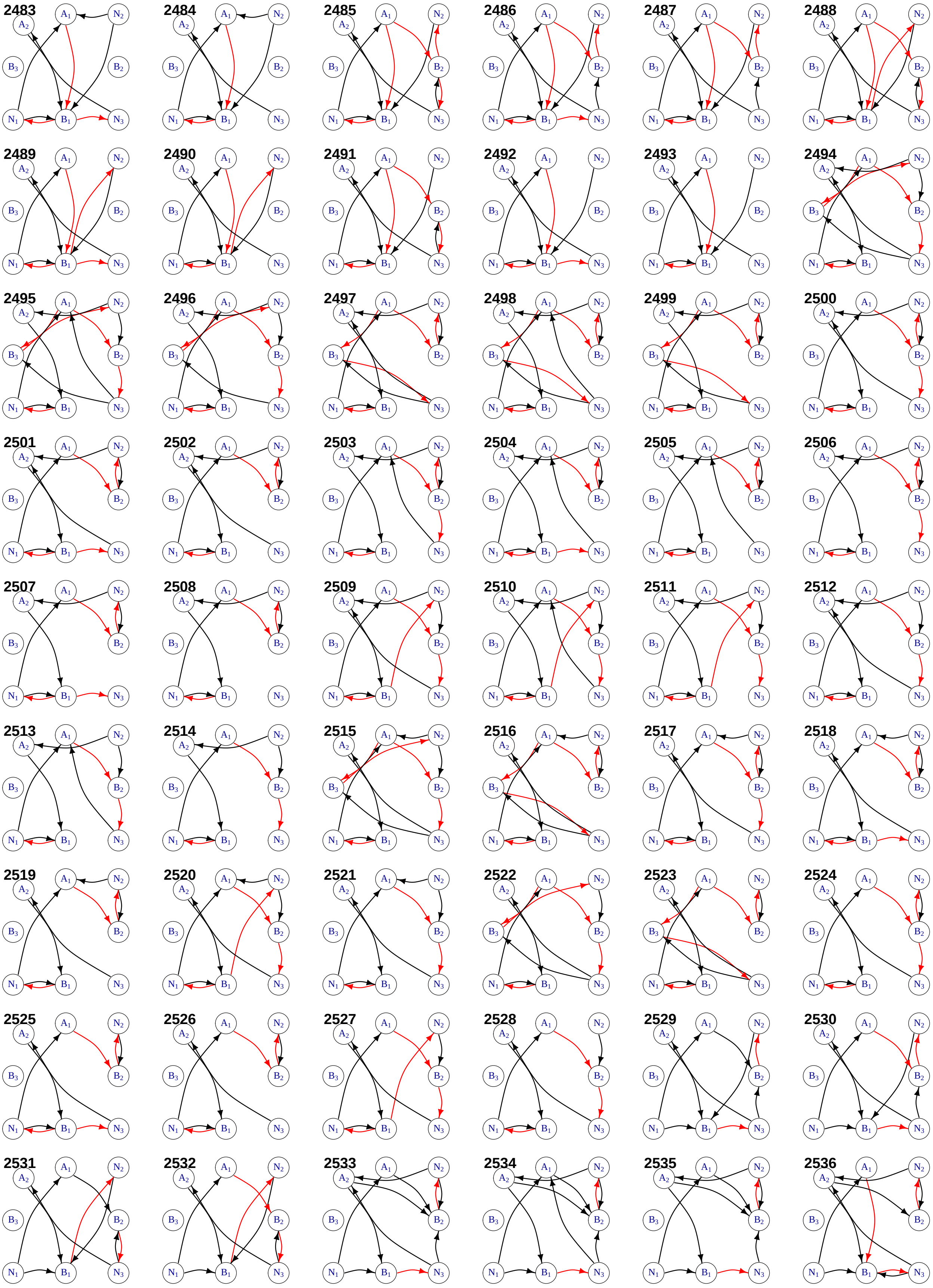
2483
2484
2485
2486
2487
2488
2489
2490
2491
2492
2493
2494
2495
2496
2497
2498
2499
2500
2501
2502
2503
2504
2505
2506
2507
2508
2509
2510
2511
2512
2513
2514
2515
2516
2517
2518
2519
2520
2521
2522
2523
2524
2525
2526
2527
2528
2529
2530
2531
2532
2533
2534
2535
2536

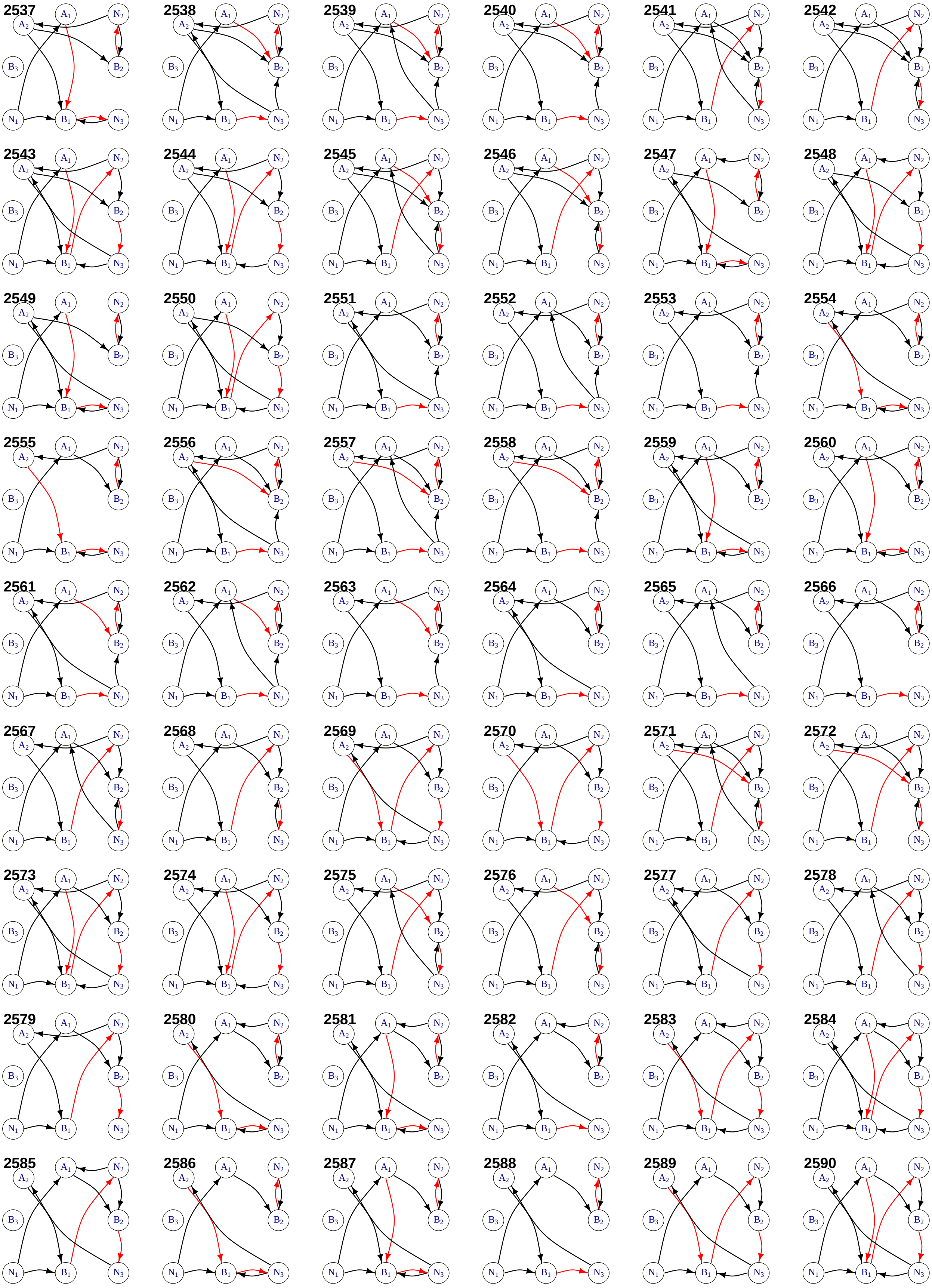
2537
2538
2539
2540
2541
2542
2543
2544
2545
2546
2547
2548
2549
2550
2551
2552
2553
2554
2555
2556
2557
2558
2559
2560
2561
2562
2563
2564
2565
2566
2567
2568
2569
2570
2571
2572
2573
2574
2575
2576
2577
2578
2579
2580
2581
2582
2583
2584
2585
2586
2587
2588
2589
2590

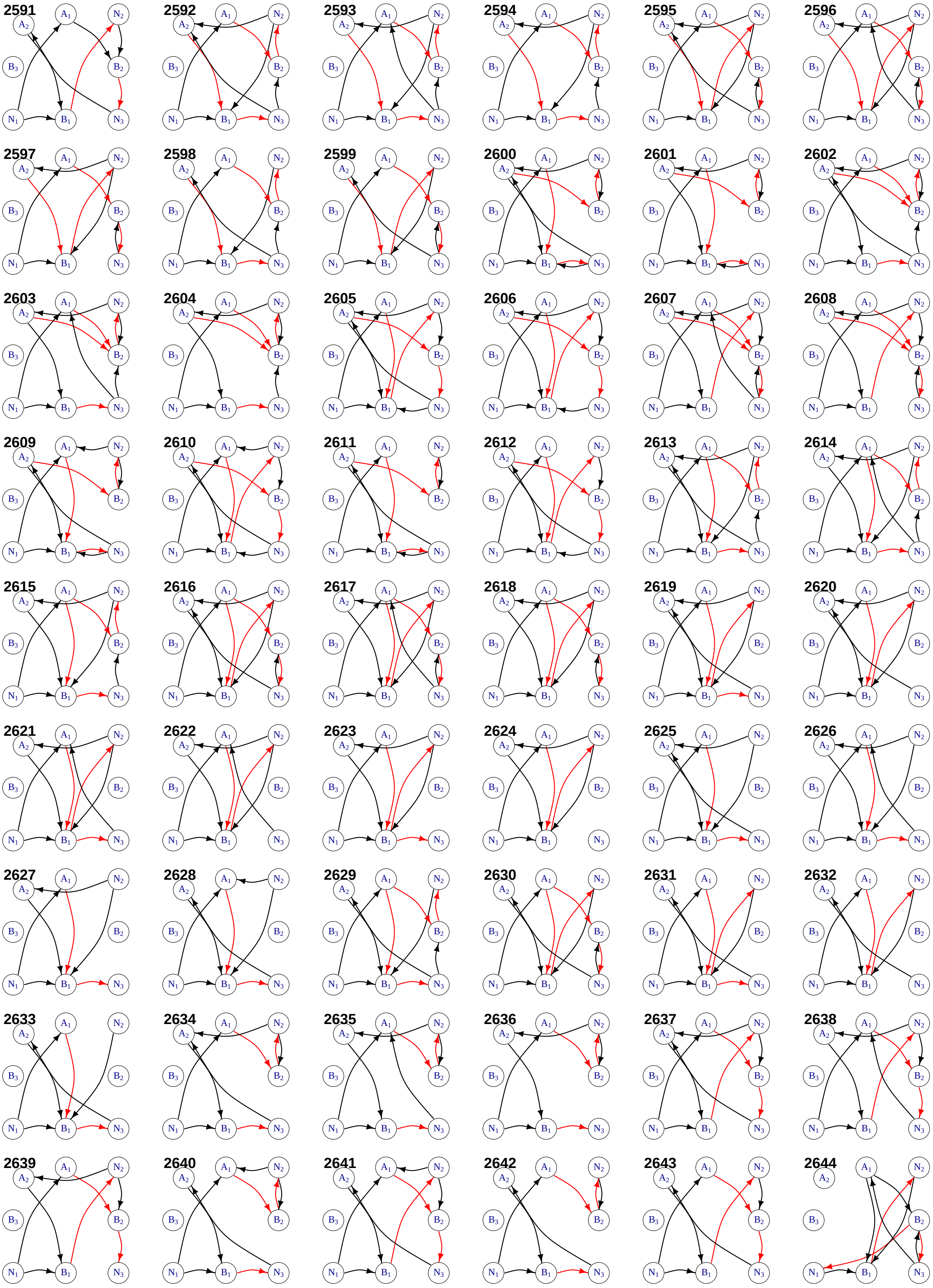
2591
2592
2593
2594
2595
2596
2597
2598
2599
2600
2601
2602
2603
2604
2605
2606
2607
2608
2609
2610
2611
2612
2613
2614
2615
2616
2617
2618
2619
2620
2621
2622
2623
2624
2625
2626
2627
2628
2629
2630
2631
2632
2633
2634
2635
2636
2637
2638
2639
2640
2641
2642
2643
2644

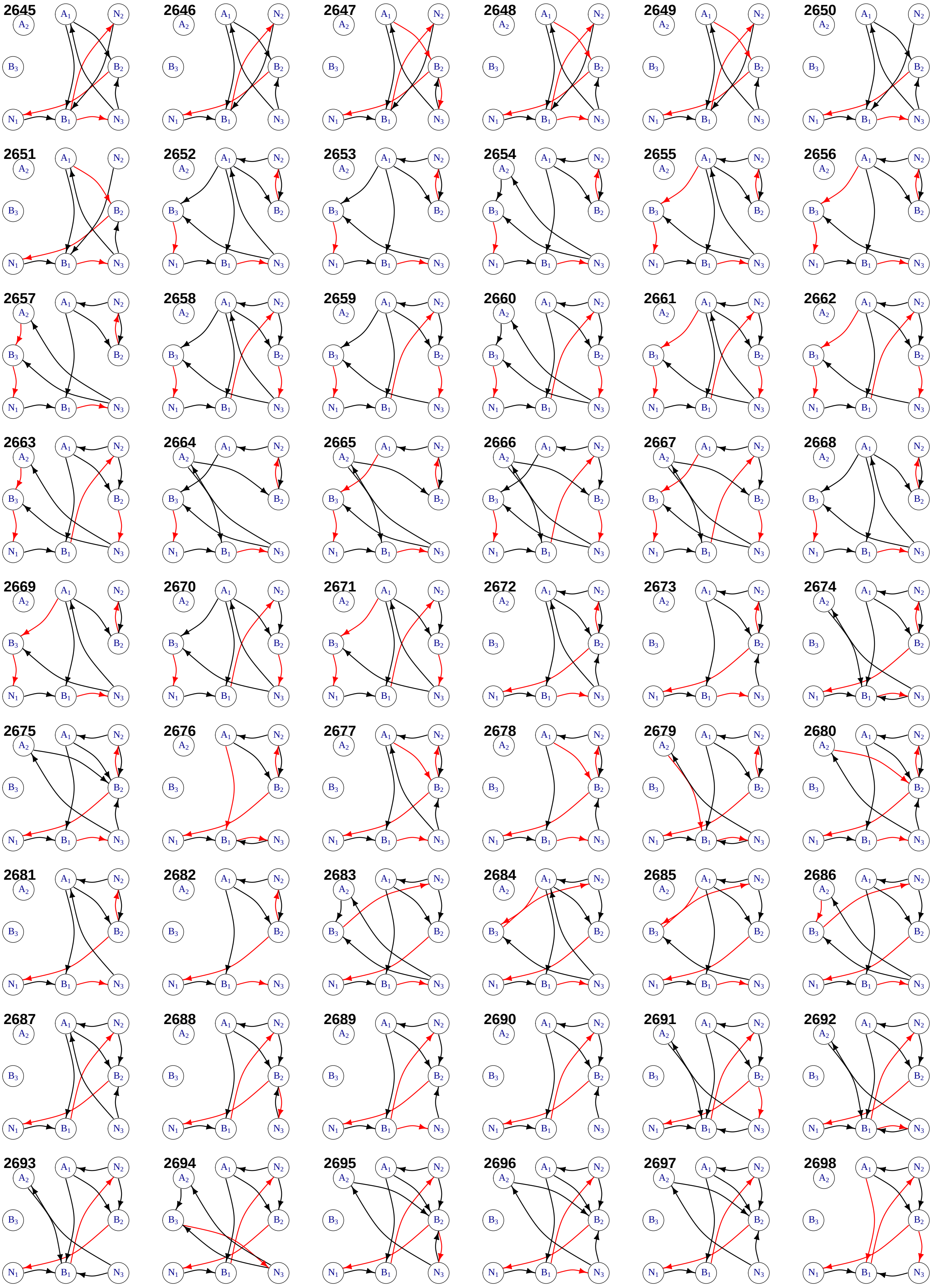
2645
2646
2647
2648
2649
2650
2651
2652
2653
2654
2655
2656
2657
2658
2659
2660
2661
2662
2663
2664
2665
2666
2667
2668
2669
2670
2671
2672
2673
2674
2675
2676
2677
2678
2679
2680
2681
2682
2683
2684
2685
2686
2687
2688
2689
2690
2691
2692
2693
2694
2695
2696
2697
2698

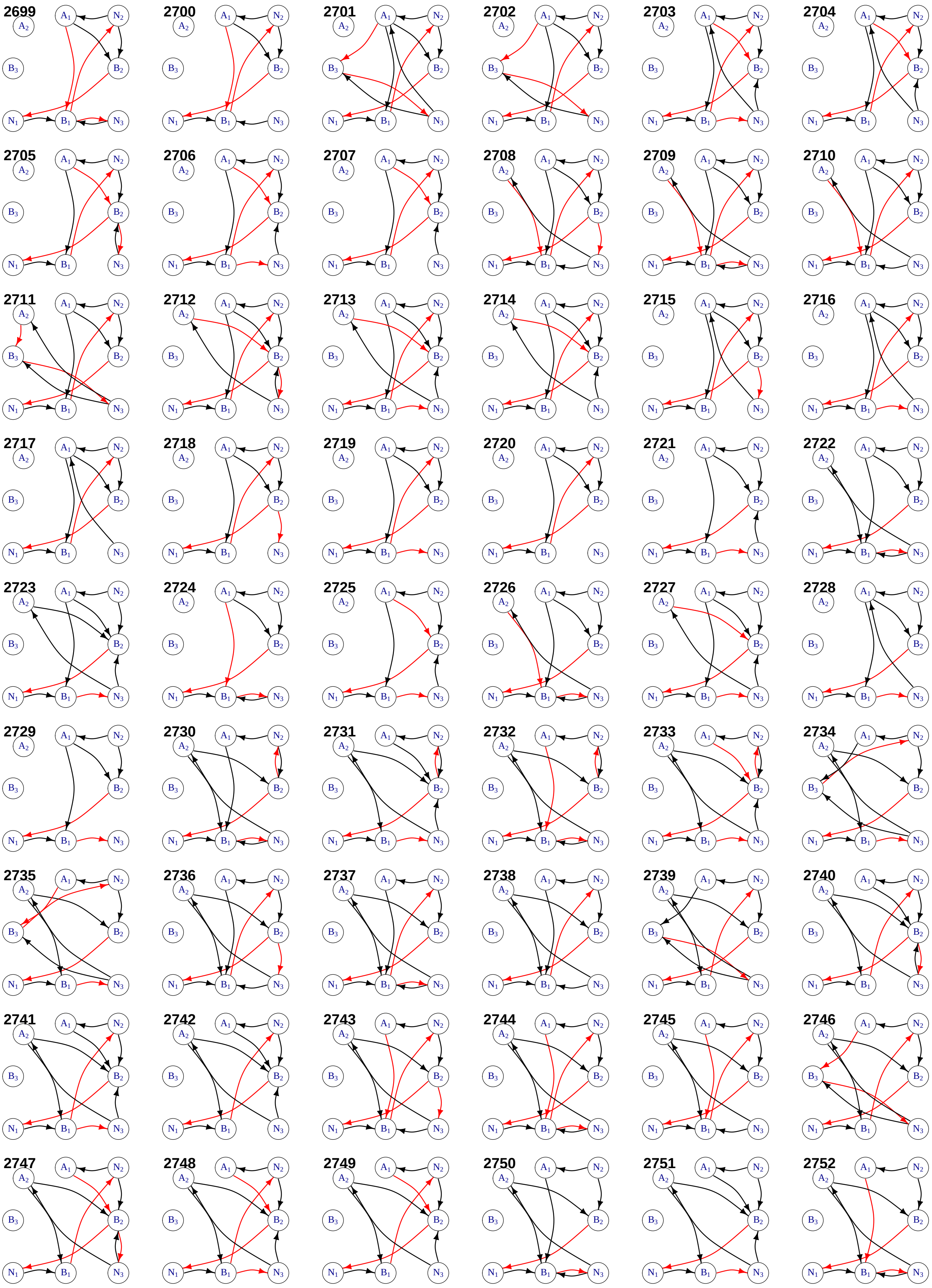
2699
2700
2701
2702
2703
2704
2705
2706
2707
2708
2709
2710
2711
2712
2713
2714
2715
2716
2717
2718
2719
2720
2721
2722
2723
2724
2725
2726
2727
2728
2729
2730
2731
2732
2733
2734
2735
2736
2737
2738
2739
2740
2741
2742
2743
2744
2745
2746
2747
2748
2749
2750
2751
2752

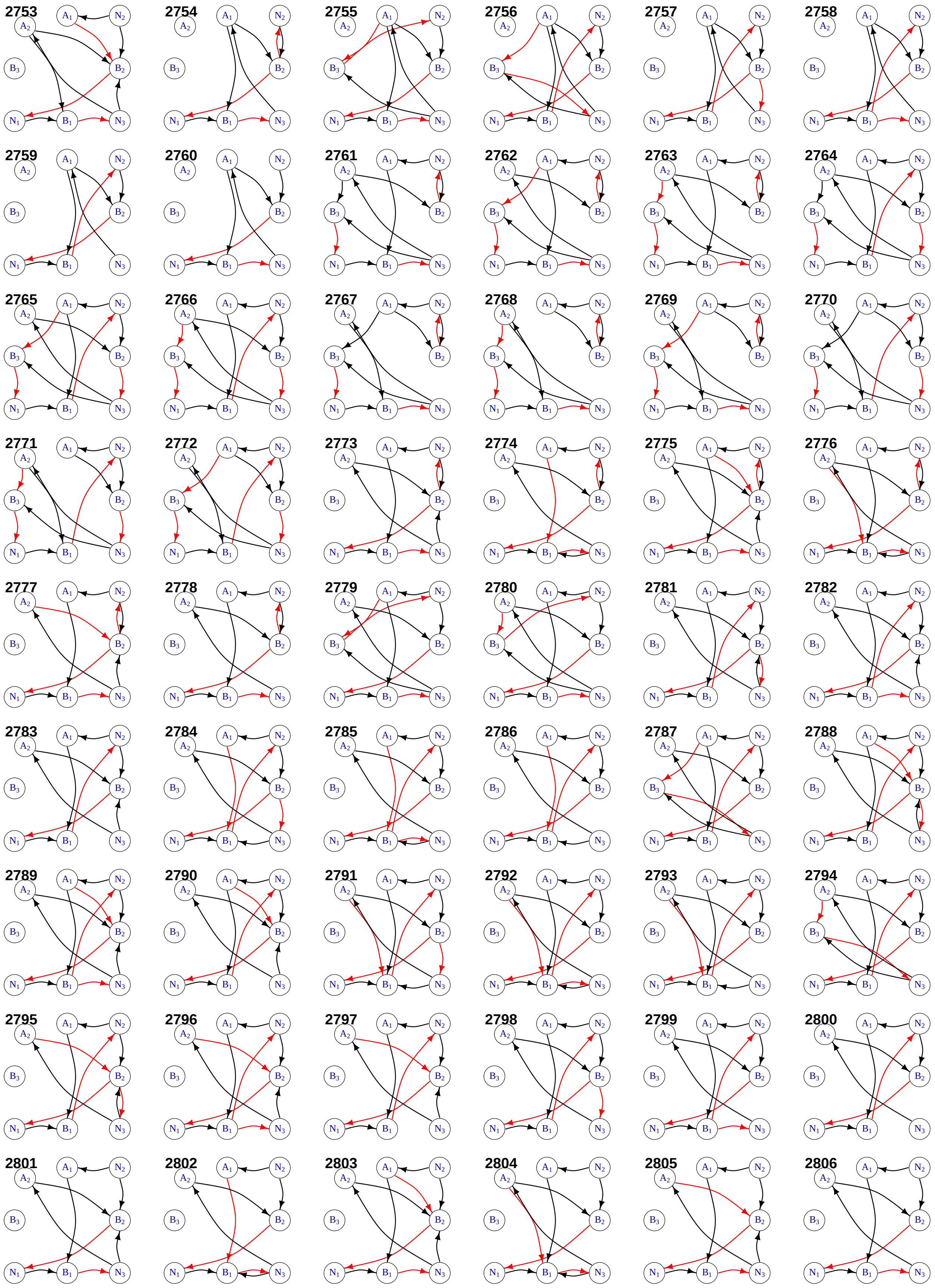
2753
2754
2755
2756
2757
2758
2759
2760
2761
2762
2763
2764
2765
2766
2767
2768
2769
2770
2771
2772
2773
2774
2775
2776
2777
2778
2779
2780
2781
2782
2783
2784
2785
2786
2787
2788
2789
2790
2791
2792
2793
2794
2795
2796
2797
2798
2799
2800
2801
2802
2803
2804
2805
2806

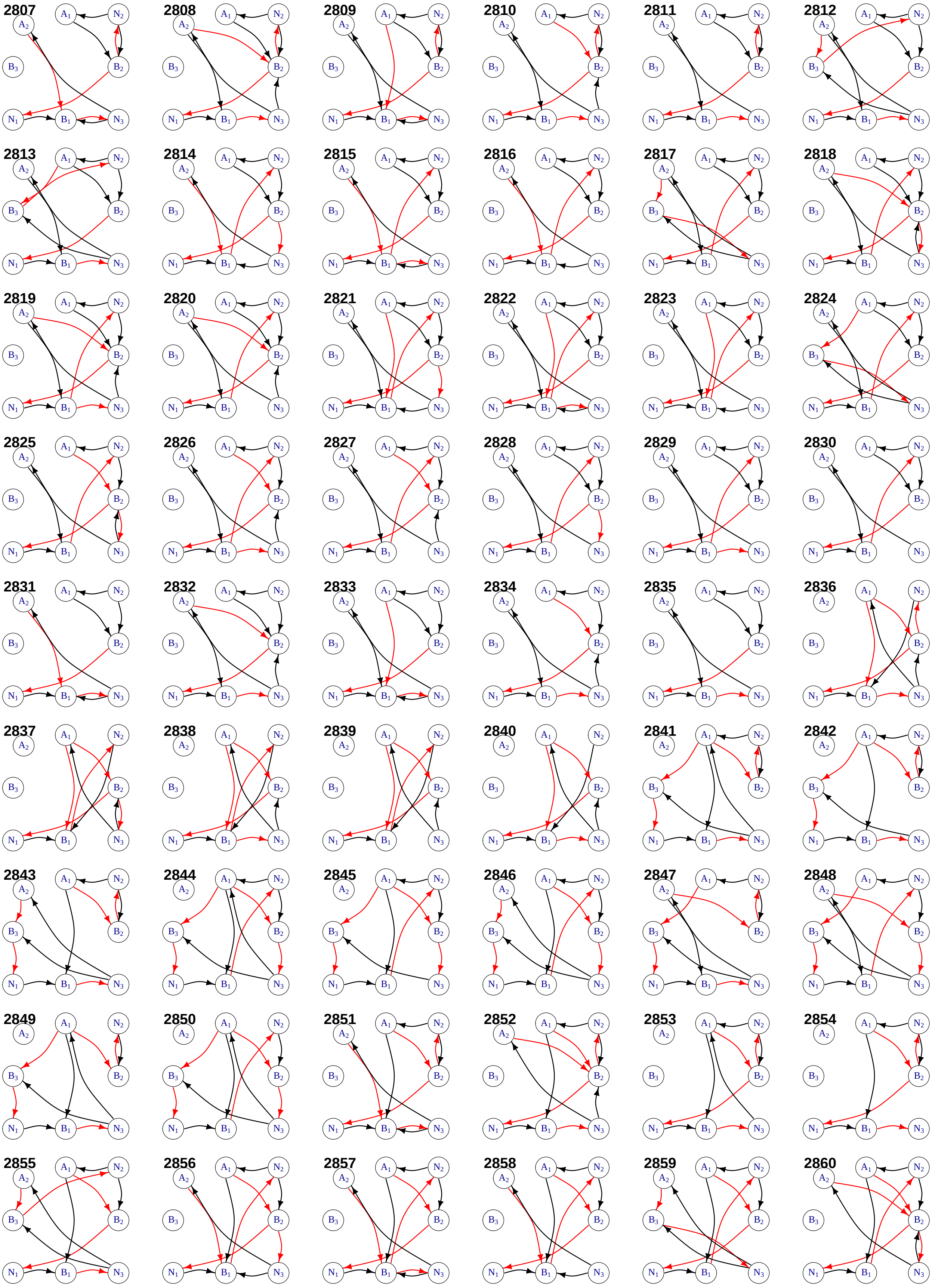

2807 2808 2809 2810 2811 2812
2813 2814 2815 2816 2817 2818
2819 2820 2821 2822 2823 2824
2825 2826 2827 2828 2829 2830
2831 2832 2833 2834 2835 2836
2837 2838 2839 2840 2841 2842
2843 2844 2845 2846 2847 2848
2849 2850 2851 2852 2853 2854
2855 2856 2857 2858 2859 2860

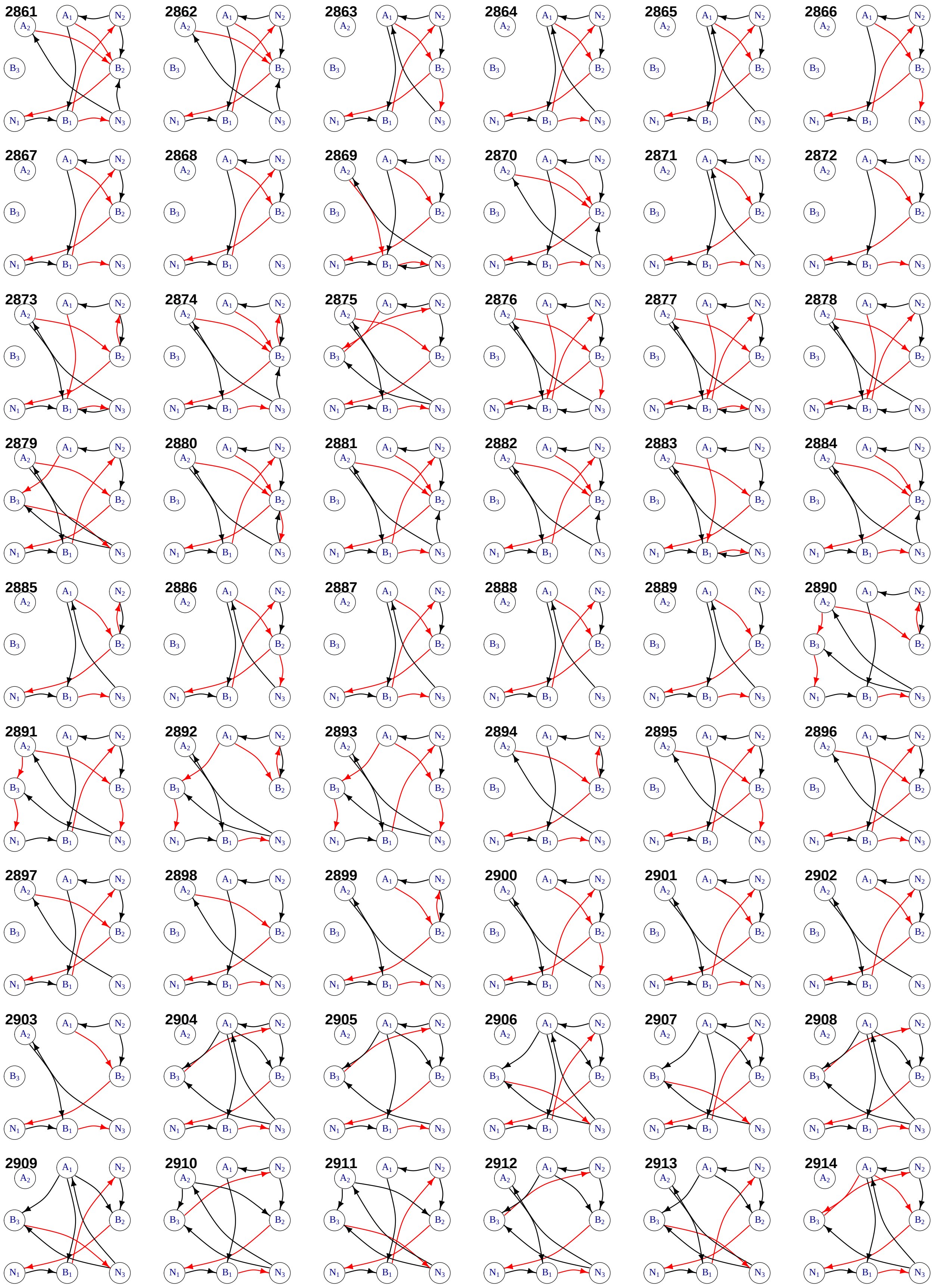
2861 2862 2863 2864 2865 2866
2867 2868 2869 2870 2871 2872
2873 2874 2875 2876 2877 2878
2879 2880 2881 2882 2883 2884
2885 2886 2887 2888 2889 2890
2891 2892 2893 2894 2895 2896
2897 2898 2899 2900 2901 2902
2903 2904 2905 2906 2907 2908
2909 2910 2911 2912 2913 2914

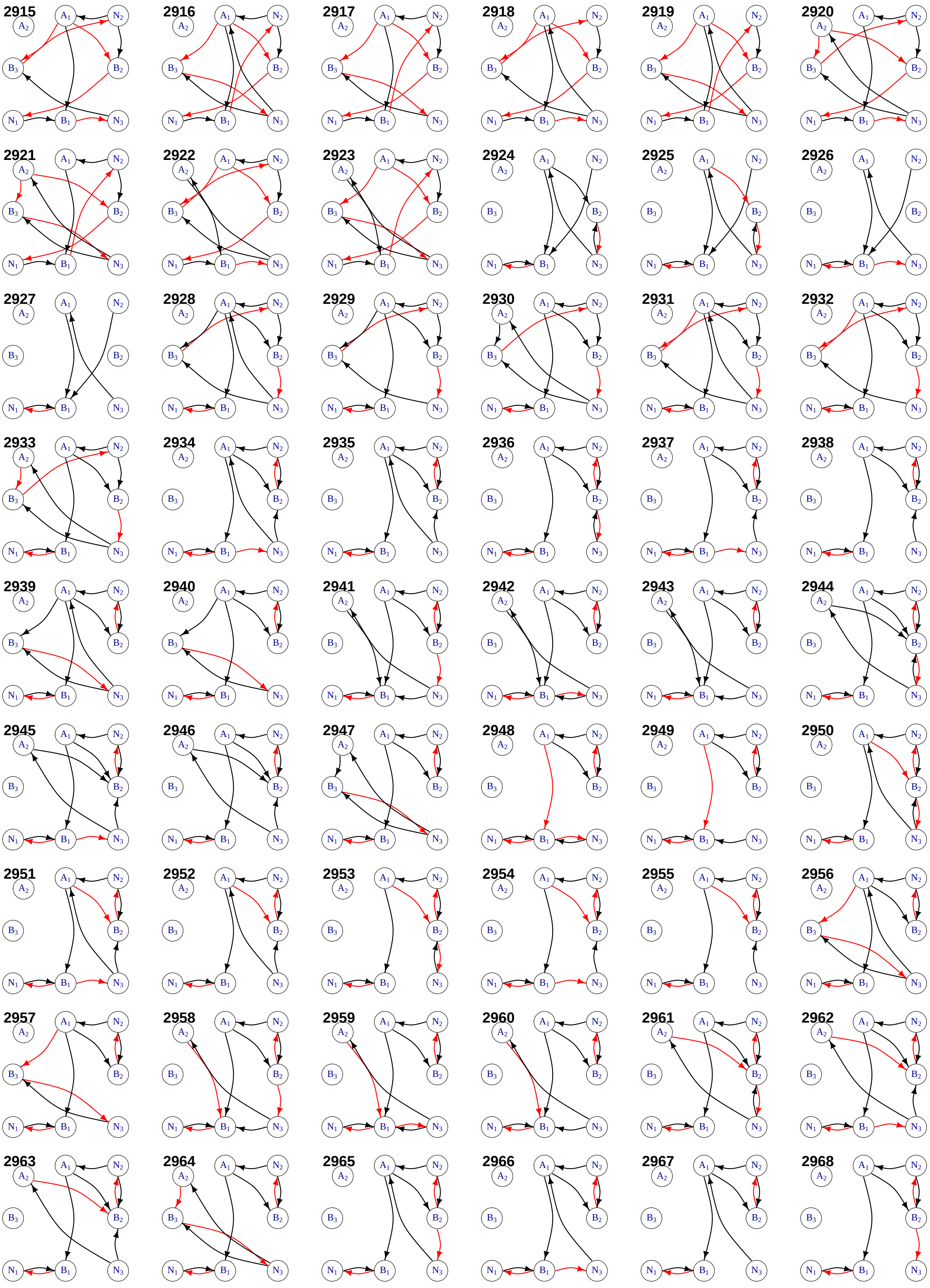
2915
2916
2917
2918
2919
2920
2921
2922
2923
2924
2925
2926
2927
2928
2929
2930
2931
2932
2933
2934
2935
2936
2937
2938
2939
2940
2941
2942
2943
2944
2945
2946
2947
2948
2949
2950
2951
2952
2953
2954
2955
2956
2957
2958
2959
2960
2961
2962
2963
2964
2965
2966
2967
2968

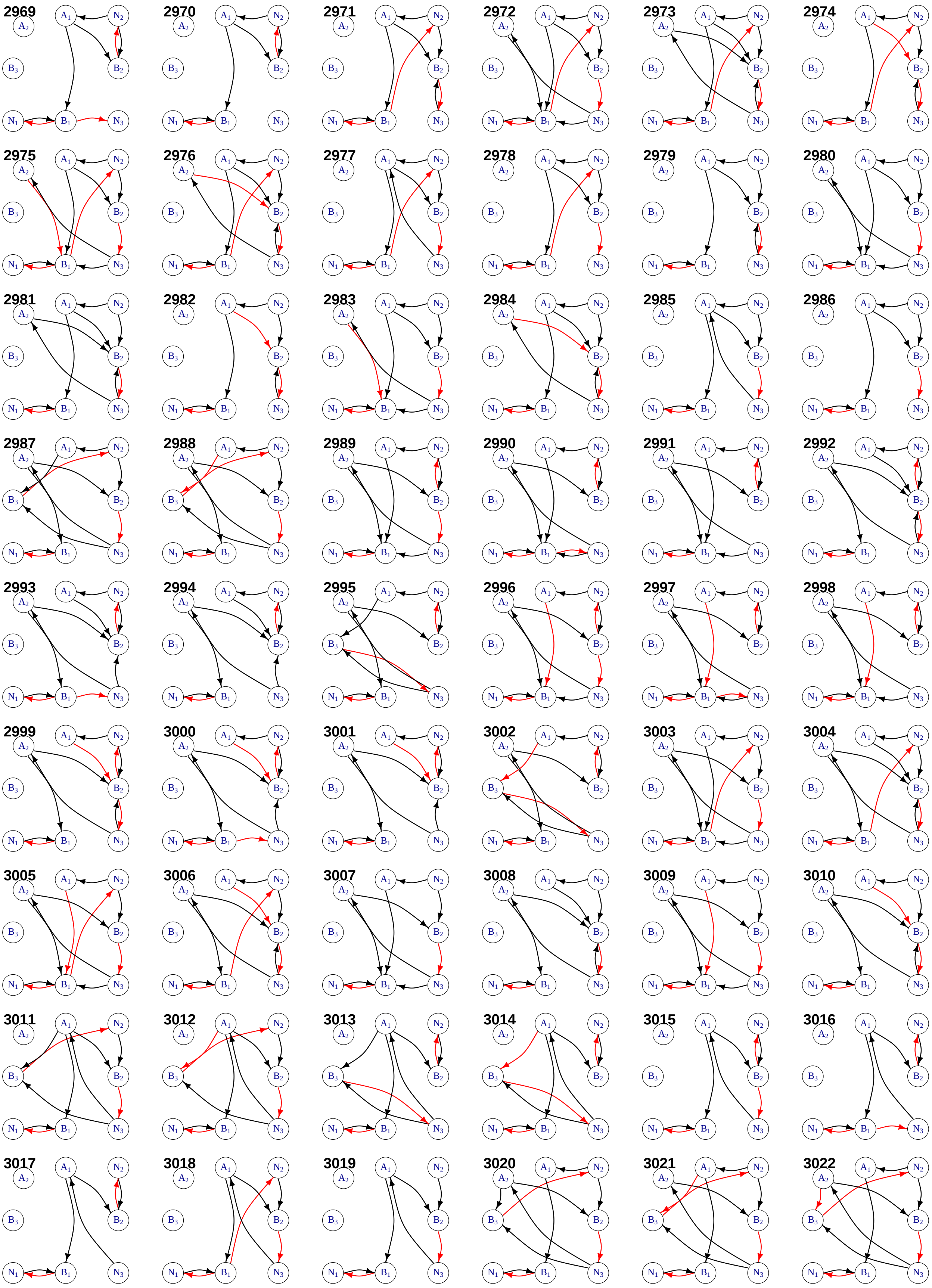
2969
2970
2971
2972
2973
2974
2975
2976
2977
2978
2979
2980
2981
2982
2983
2984
2985
2986
2987
2988
2989
2990
2991
2992
2993
2994
2995
2996
2997
2998
2999
3000
3001
3002
3003
3004
3005
3006
3007
3008
3009
3010
3011
3012
3013
3014
3015
3016
3017
3018
3019
3020
3021
3022

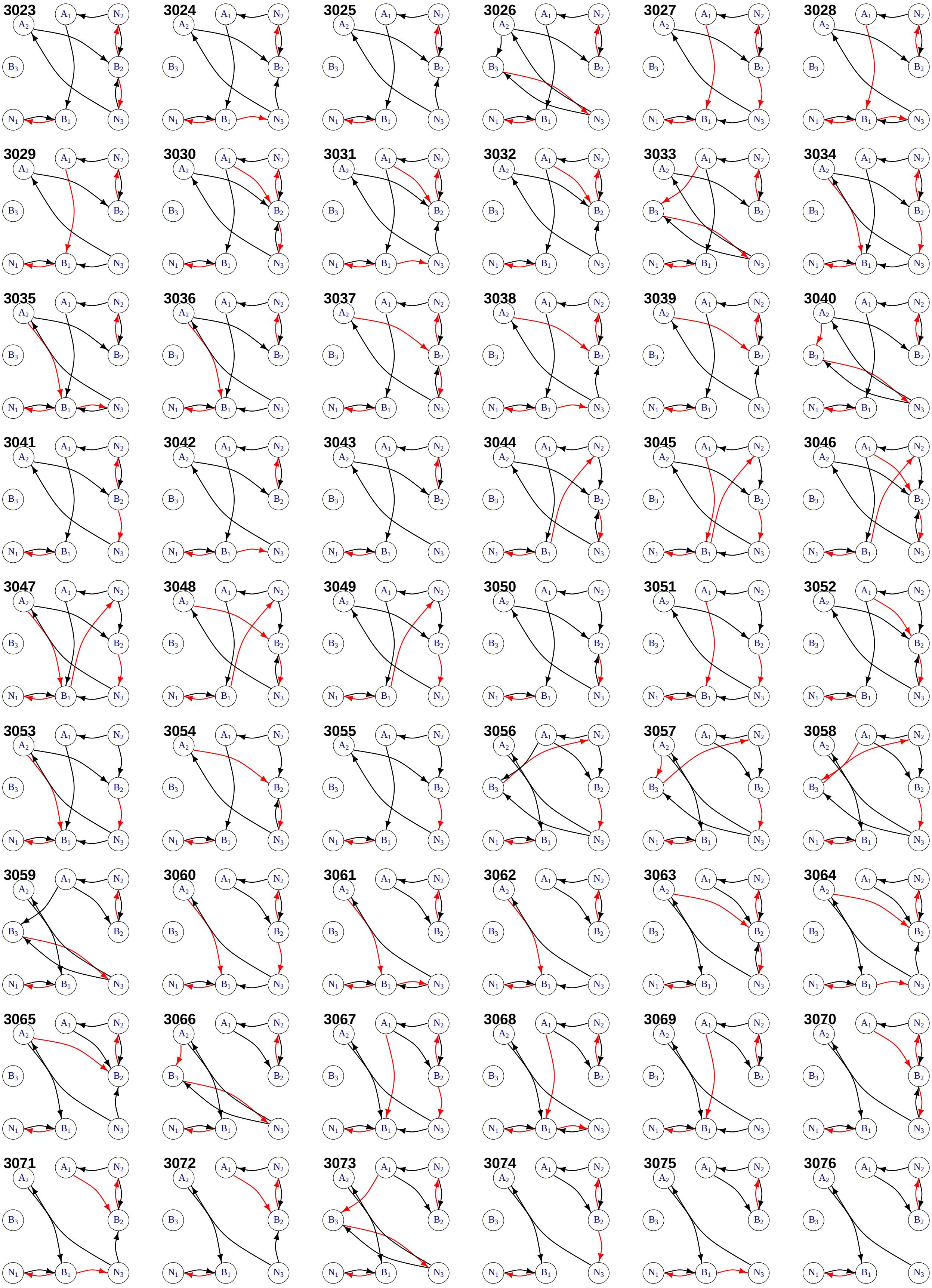
3023
3024
3025
3026
3027
3028
3029
3030
3031
3032
3033
3034
3035
3036
3037
3038
3039
3040
3041
3042
3043
3044
3045
3046
3047
3048
3049
3050
3051
3052
3053
3054
3055
3056
3057
3058
3059
3060
3061
3062
3063
3064
3065
3066
3067
3068
3069
3070
3071
3072
3073
3074
3075
3076

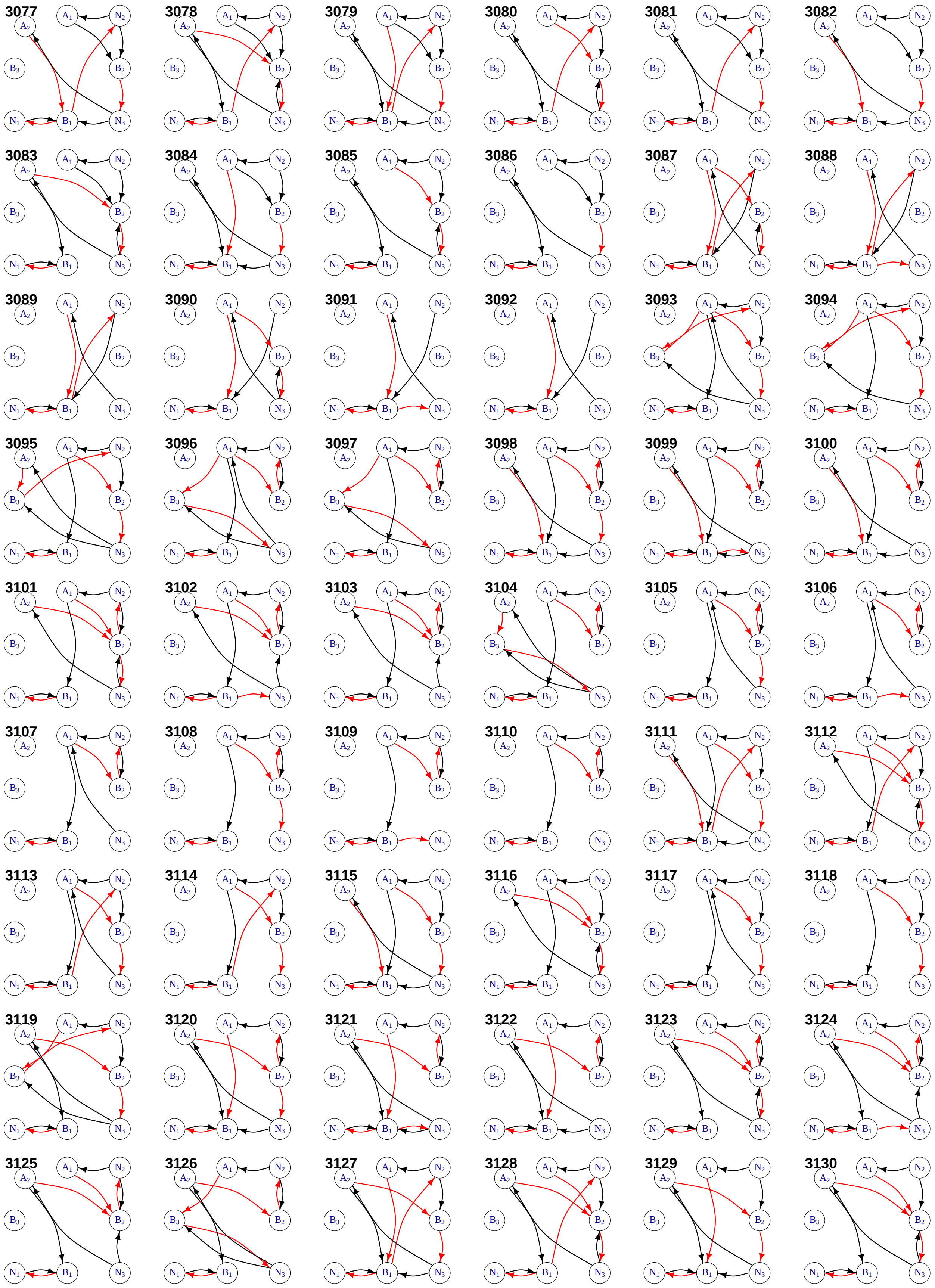
3077
3078
3079
3080
3081
3082
3083
3084
3085
3086
3087
3088
3089
3090
3091
3092
3093
3094
3095
3096
3097
3098
3099
3100
3101
3102
3103
3104
3105
3106
3107
3108
3109
3110
3111
3112
3113
3114
3115
3116
3117
3118
3119
3120
3121
3122
3123
3124
3125
3126
3127
3128
3129
3130

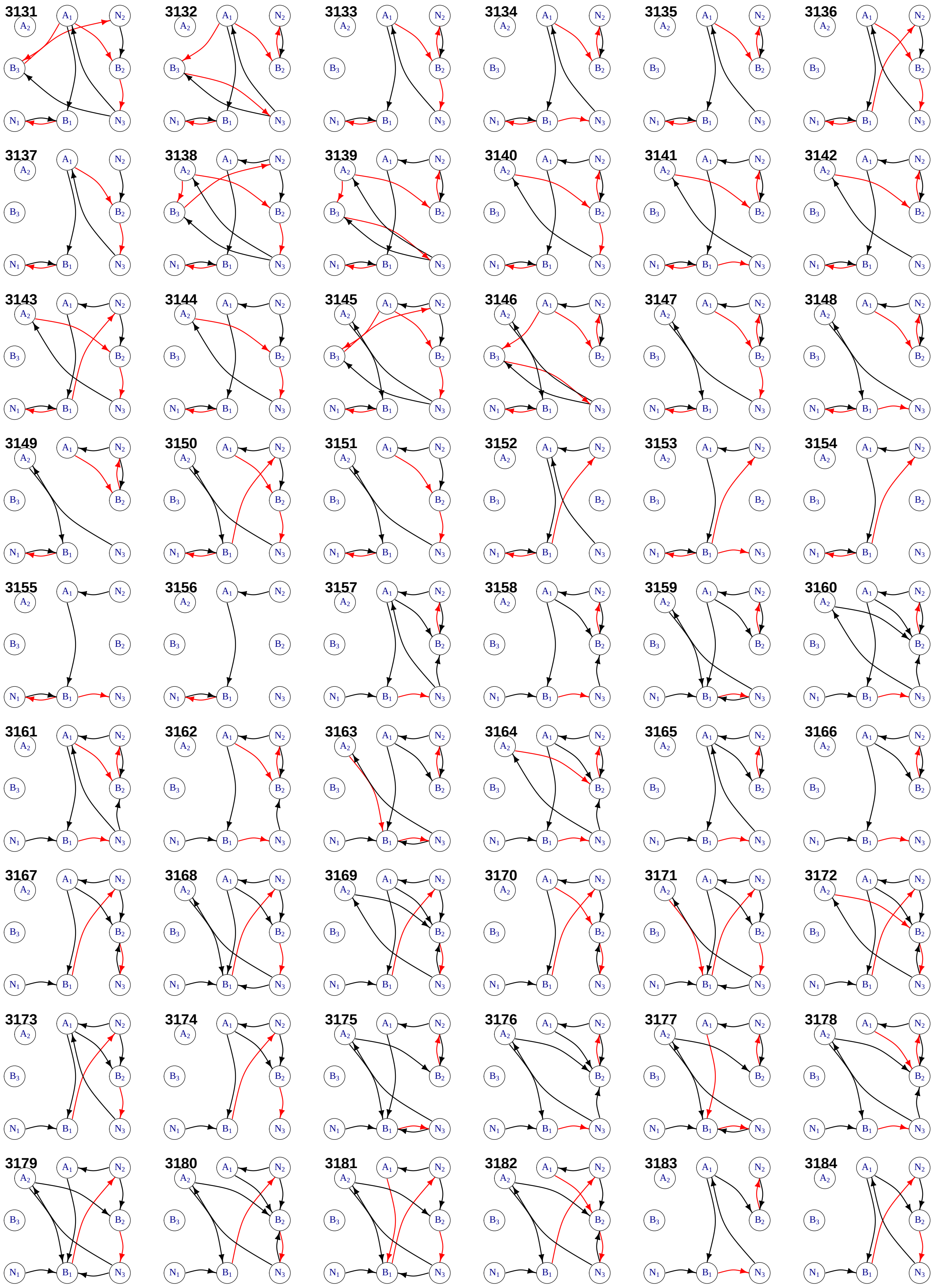
3131 3132 3133 3134 3135 3136
3137 3138 3139 3140 3141 3142
3143 3144 3145 3146 3147 3148
3149 3150 3151 3152 3153 3154
3155 3156 3157 3158 3159 3160
3161 3162 3163 3164 3165 3166
3167 3168 3169 3170 3171 3172
3173 3174 3175 3176 3177 3178
3179 3180 3181 3182 3183 3184

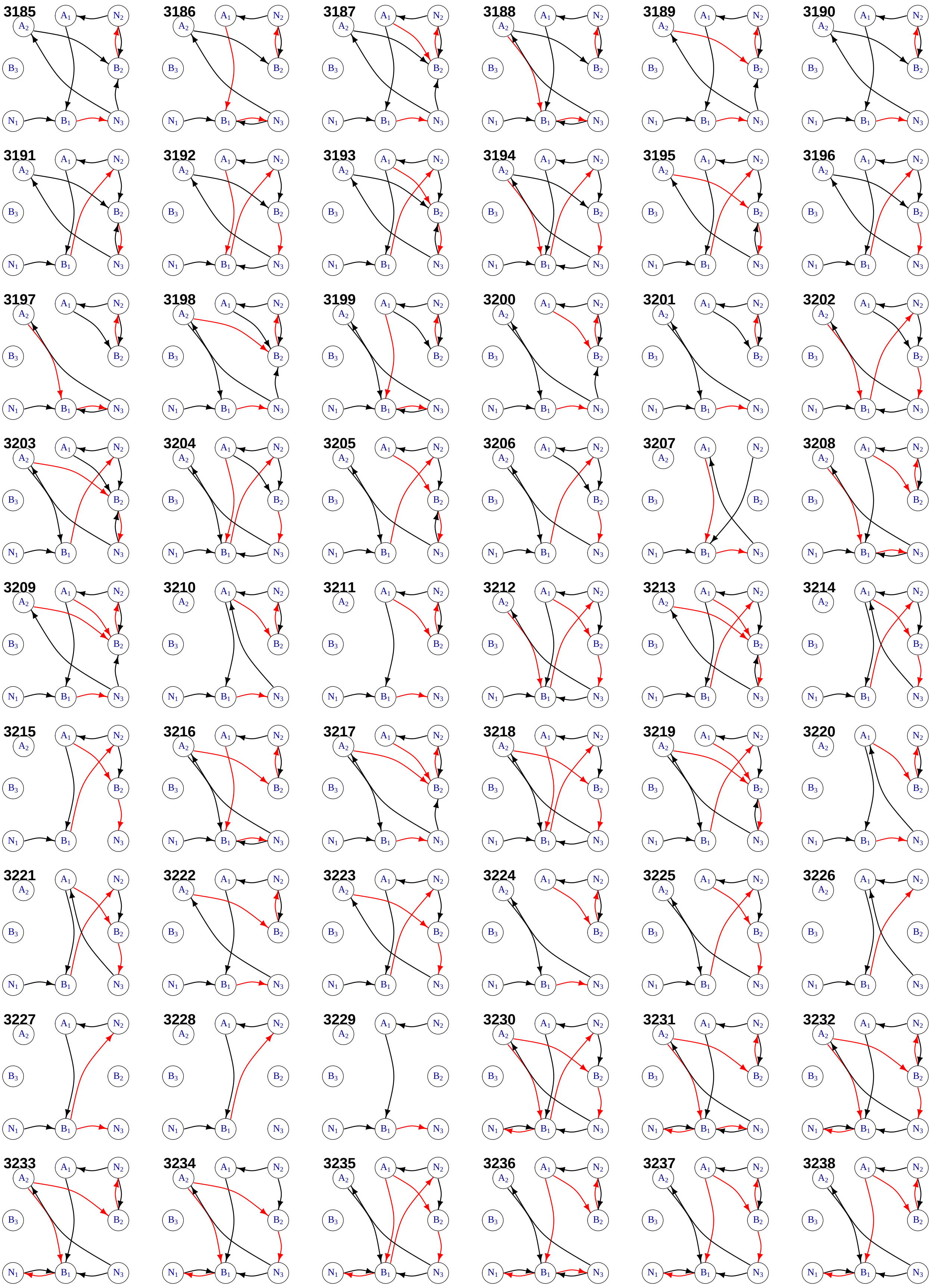
3185
3186
3187
3188
3189
3190
3191
3192
3193
3194
3195
3196
3197
3198
3199
3200
3201
3202
3203
3204
3205
3206
3207
3208
3209
3210
3211
3212
3213
3214
3215
3216
3217
3218
3219
3220
3221
3222
3223
3224
3225
3226
3227
3228
3229
3230
3231
3232
3233
3234
3235
3236
3237
3238

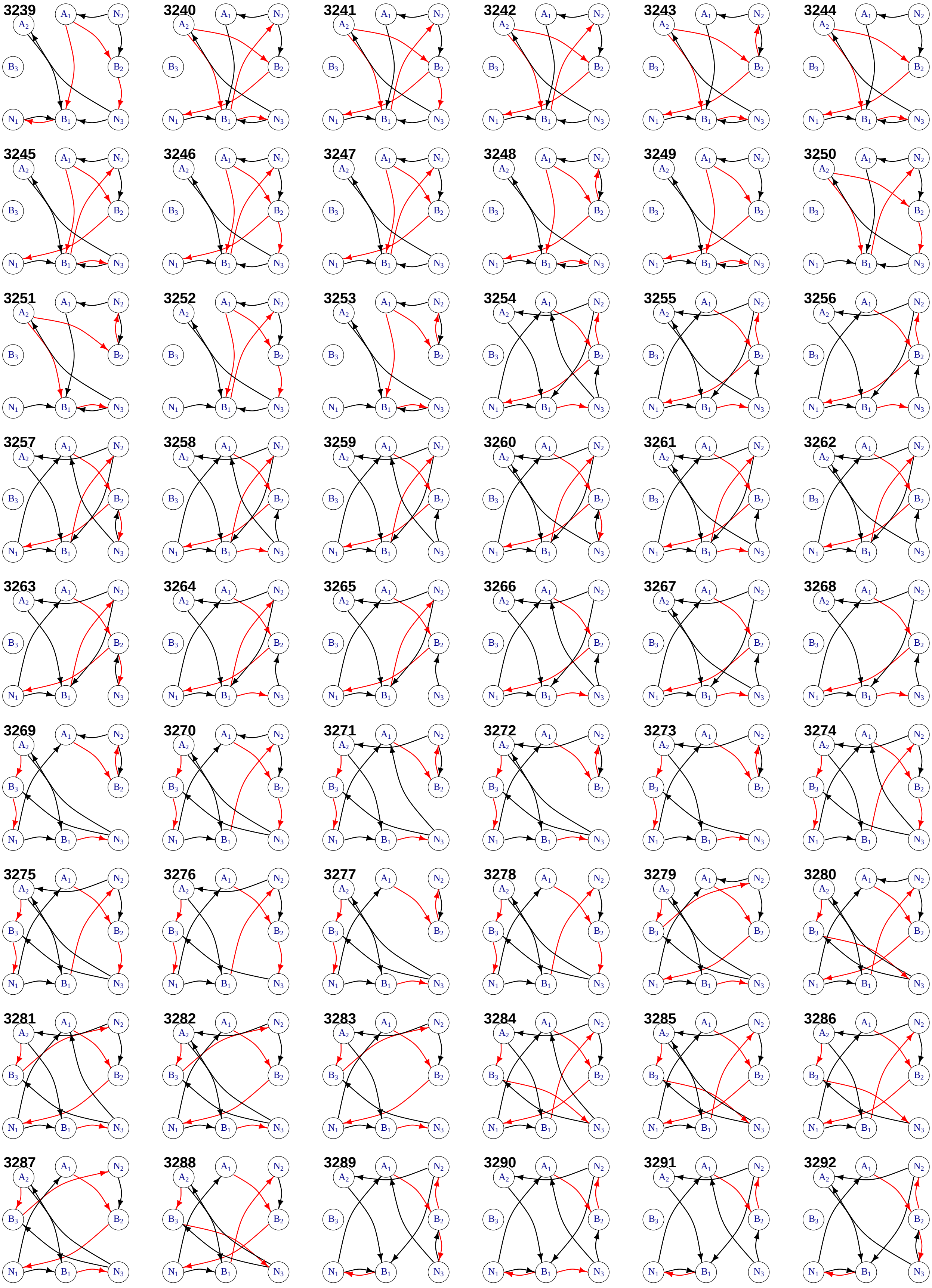
3239 3240 3241 3242 3243 3244
3245 3246 3247 3248 3249 3250
3251 3252 3253 3254 3255 3256
3257 3258 3259 3260 3261 3262
3263 3264 3265 3266 3267 3268
3269 3270 3271 3272 3273 3274
3275 3276 3277 3278 3279 3280
3281 3282 3283 3284 3285 3286
3287 3288 3289 3290 3291 3292

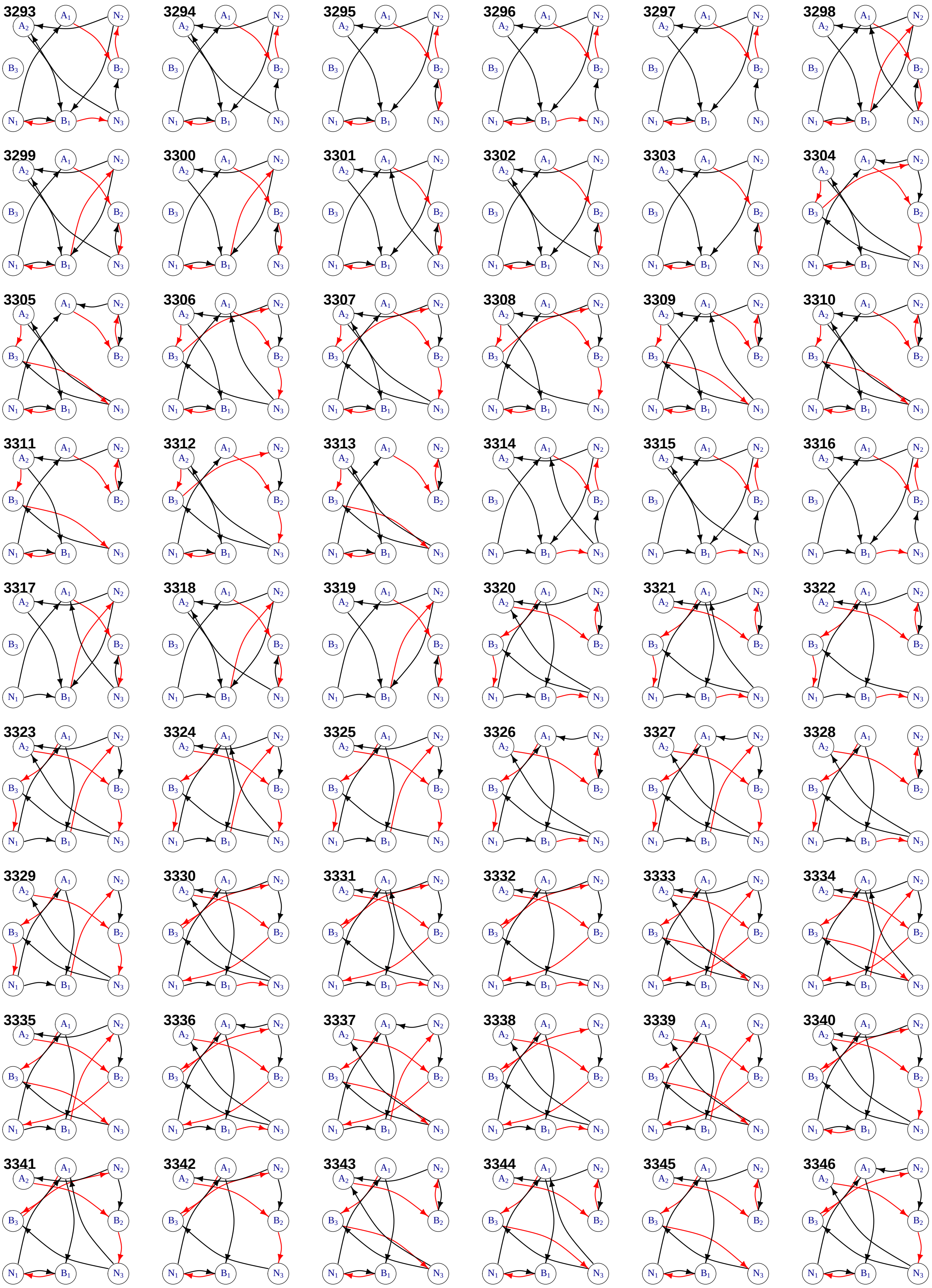
3293
3294
3295
3296
3297
3298
3299
3300
3301
3302
3303
3304
3305
3306
3307
3308
3309
3310
3311
3312
3313
3314
3315
3316
3317
3318
3319
3320
3321
3322
3323
3324
3325
3326
3327
3328
3329
3330
3331
3332
3333
3334
3335
3336
3337
3338
3339
3340
3341
3342
3343
3344
3345
3346

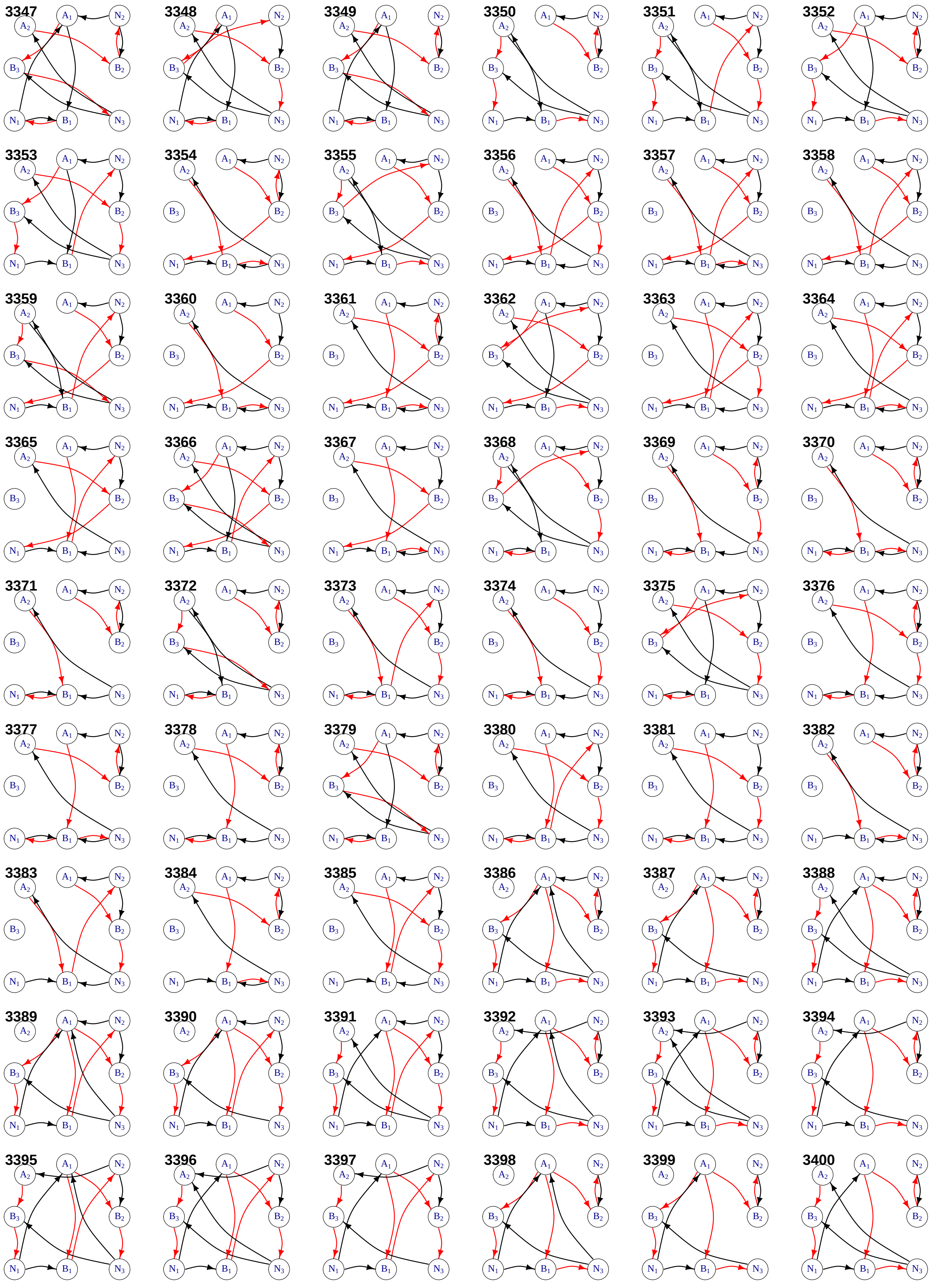
3347
3348
3349
3350
3351
3352
3353
3354
3355
3356
3357
3358
3359
3360
3361
3362
3363
3364
3365
3366
3367
3368
3369
3370
3371
3372
3373
3374
3375
3376
3377
3378
3379
3380
3381
3382
3383
3384
3385
3386
3387
3388
3389
3390
3391
3392
3393
3394
3395
3396
3397
3398
3399
3400

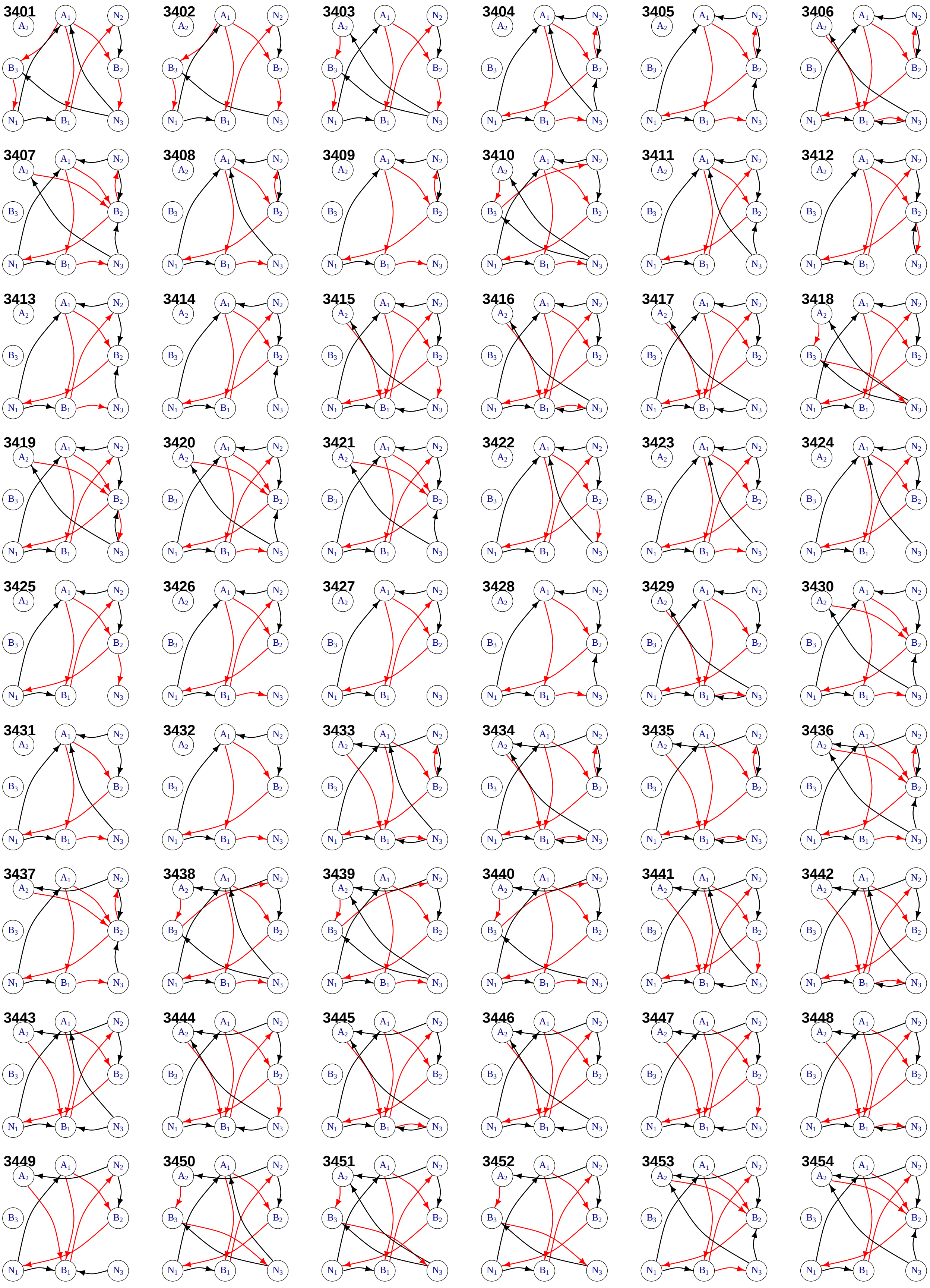
3401
3402
3403
3404
3405
3406
3407
3408
3409
3410
3411
3412
3413
3414
3415
3416
3417
3418
3419
3420
3421
3422
3423
3424
3425
3426
3427
3428
3429
3430
3431
3432
3433
3434
3435
3436
3437
3438
3439
3440
3441
3442
3443
3444
3445
3446
3447
3448
3449
3450
3451
3452
3453
3454

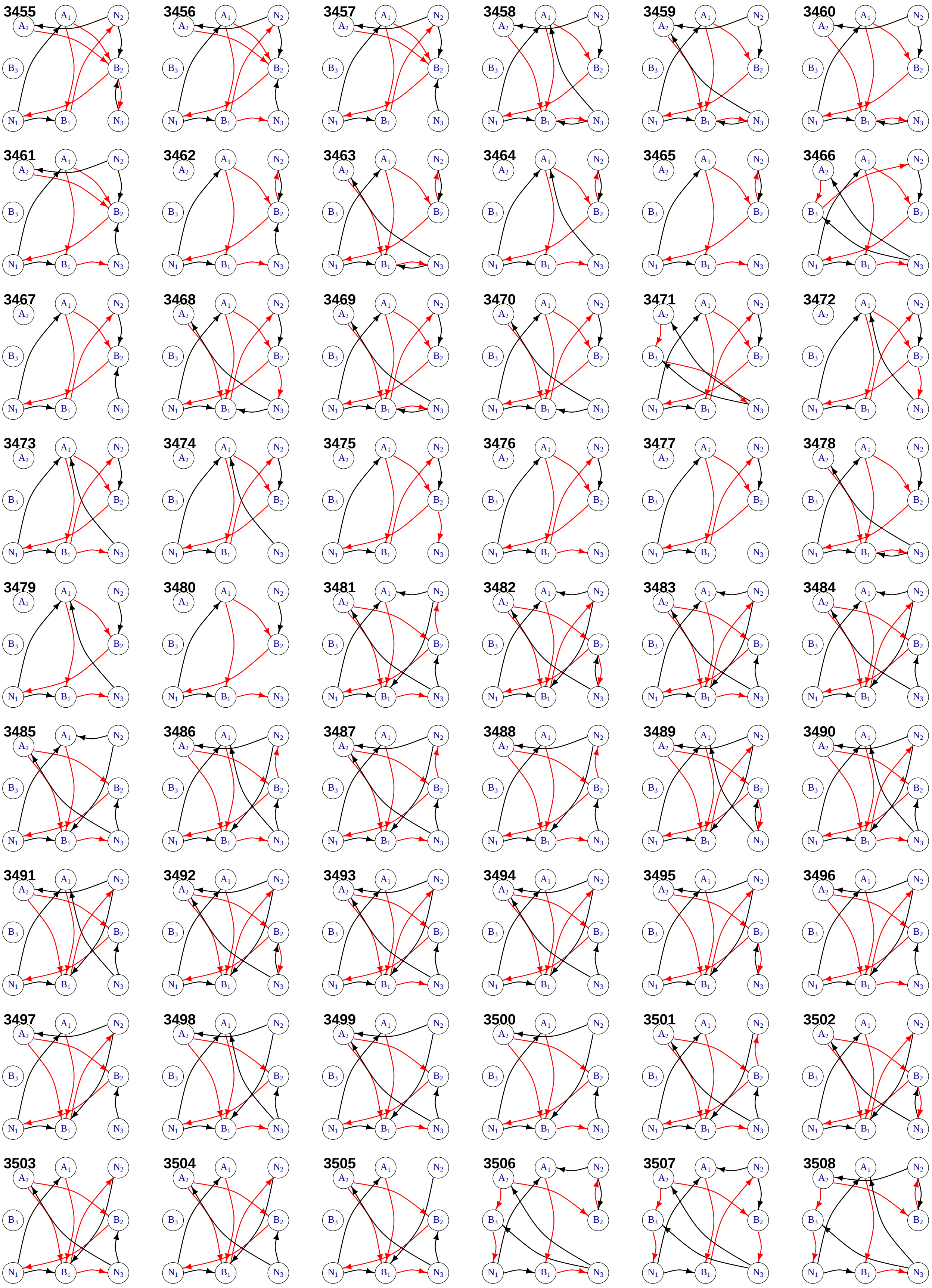
3455 3456 3457 3458 3459 3460
3461 3462 3463 3464 3465 3466
3467 3468 3469 3470 3471 3472
3473 3474 3475 3476 3477 3478
3479 3480 3481 3482 3483 3484
3485 3486 3487 3488 3489 3490
3491 3492 3493 3494 3495 3496
3497 3498 3499 3500 3501 3502
3503 3504 3505 3506 3507 3508

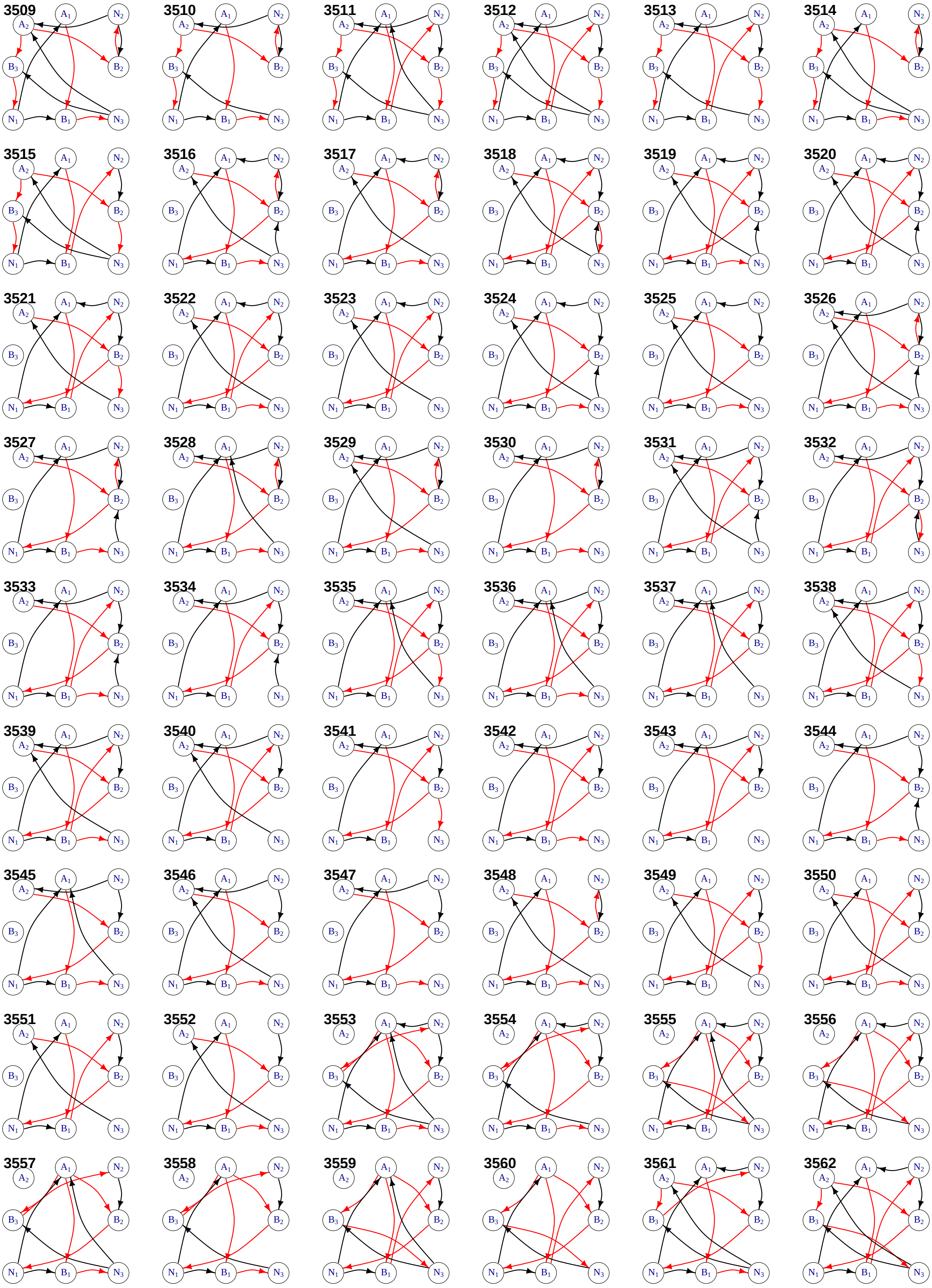
3509
3510
3511
3512
3513
3514
3515
3516
3517
3518
3519
3520
3521
3522
3523
3524
3525
3526
3527
3528
3529
3530
3531
3532
3533
3534
3535
3536
3537
3538
3539
3540
3541
3542
3543
3544
3545
3546
3547
3548
3549
3550
3551
3552
3553
3554
3555
3556
3557
3558
3559
3560
3561
3562

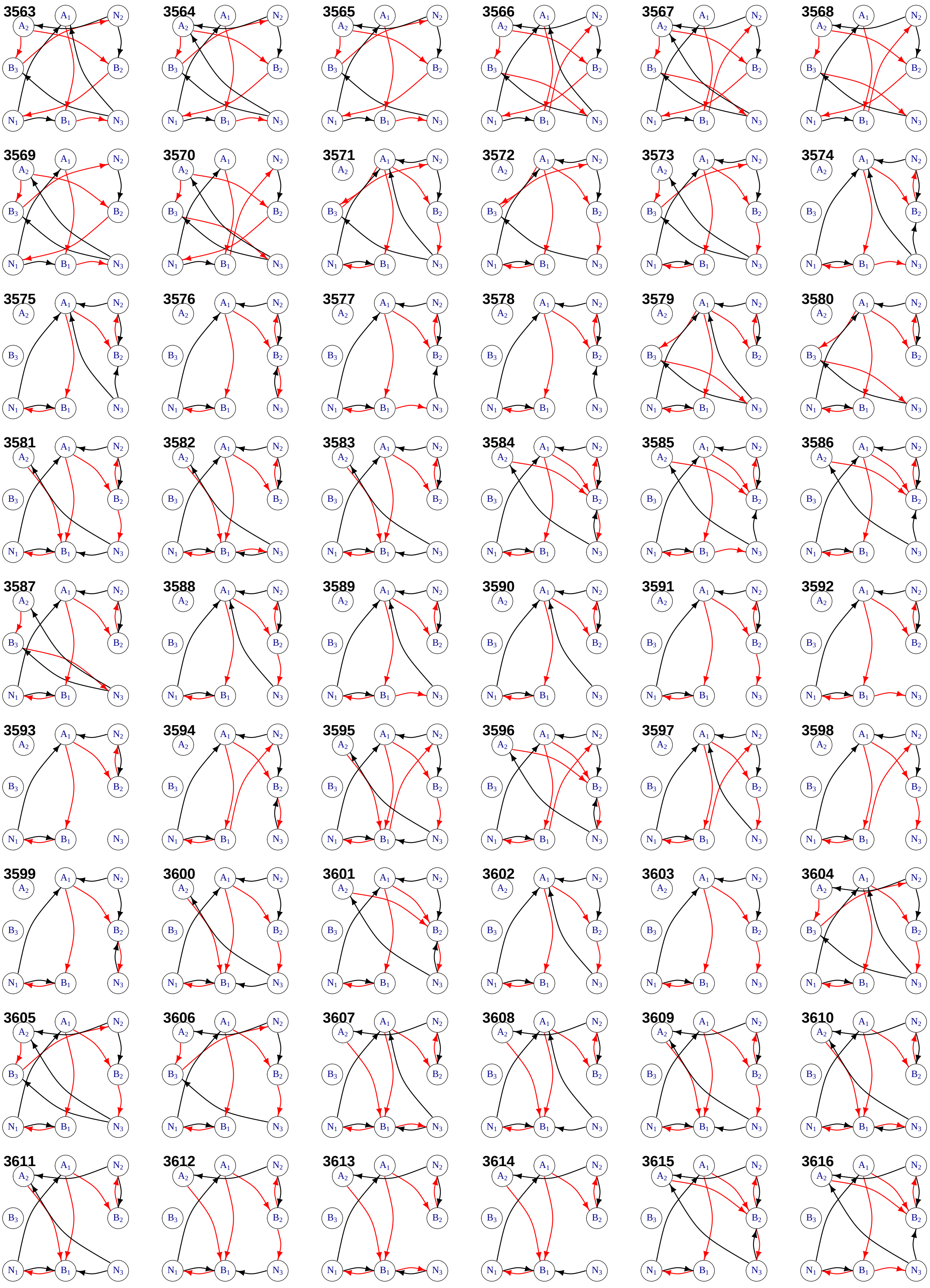
3563 3564 3565 3566 3567 3568
3569 3570 3571 3572 3573 3574
3575 3576 3577 3578 3579 3580
3581 3582 3583 3584 3585 3586
3587 3588 3589 3590 3591 3592
3593 3594 3595 3596 3597 3598
3599 3600 3601 3602 3603 3604
3605 3606 3607 3608 3609 3610
3611 3612 3613 3614 3615 3616

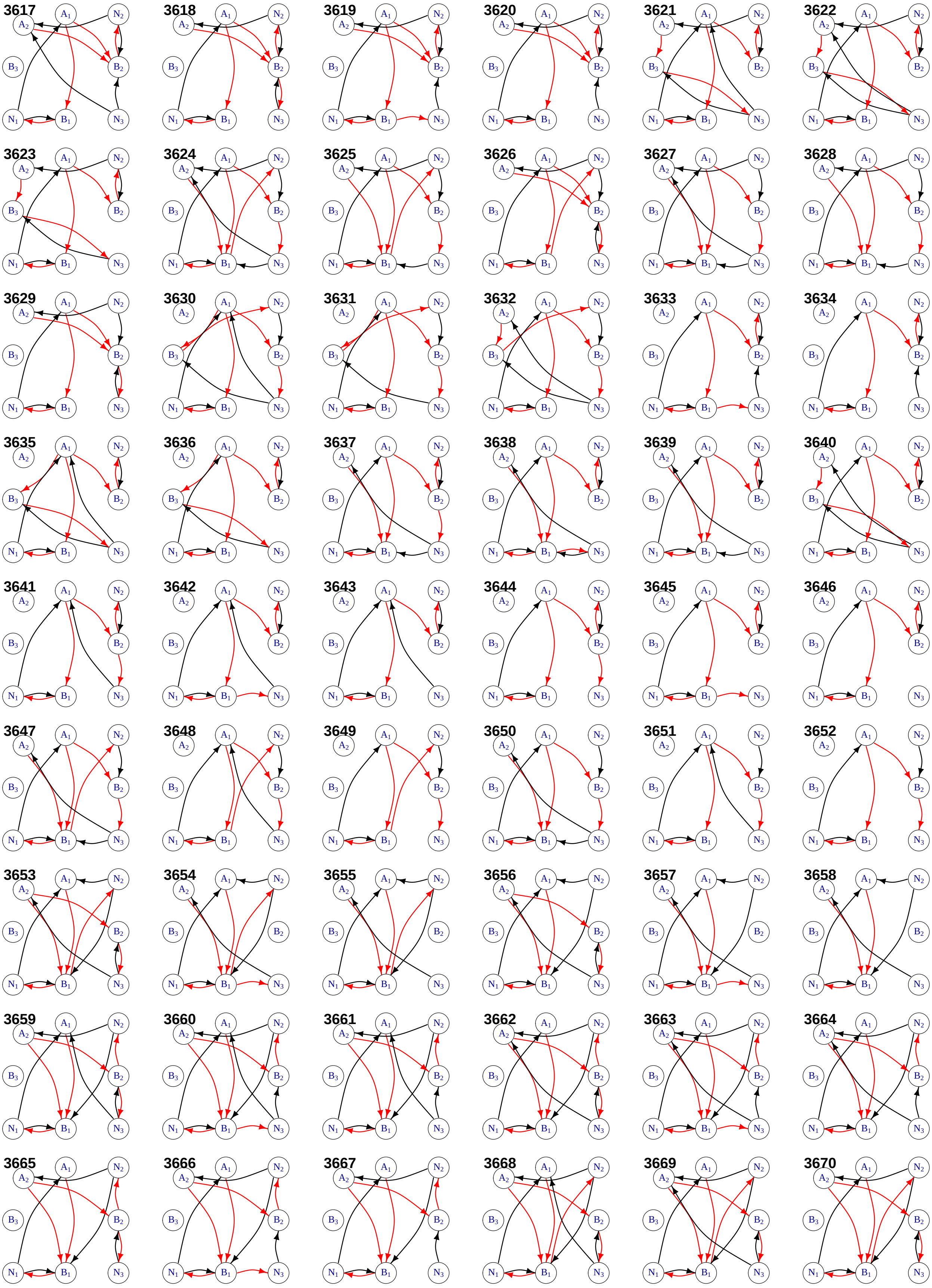
3617
3618
3619
3620
3621
3622
3623
3624
3625
3626
3627
3628
3629
3630
3631
3632
3633
3634
3635
3636
3637
3638
3639
3640
3641
3642
3643
3644
3645
3646
3647
3648
3649
3650
3651
3652
3653
3654
3655
3656
3657
3658
3659
3660
3661
3662
3663
3664
3665
3666
3667
3668
3669
3670

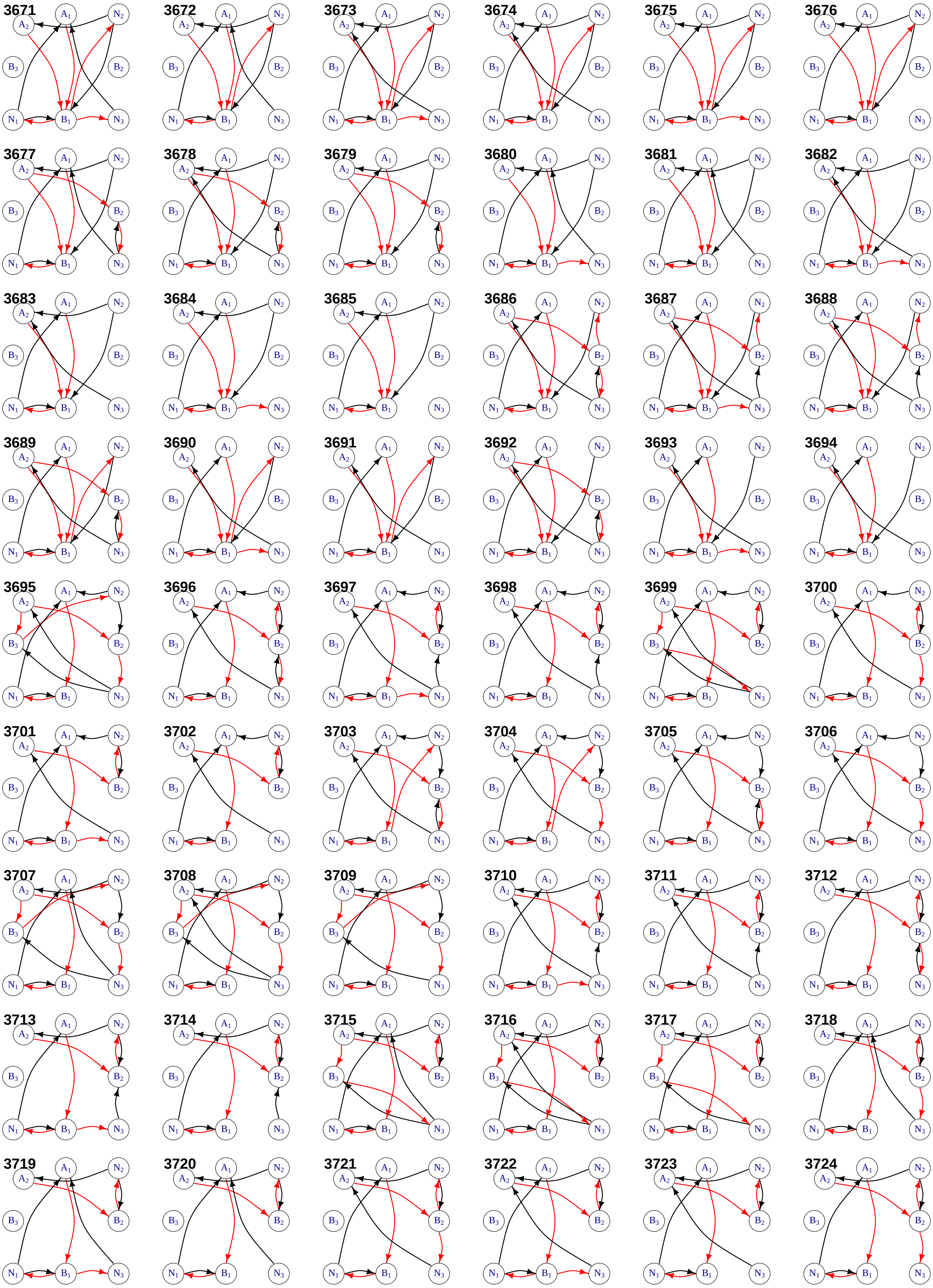
3671
3672
3673
3674
3675
3676
3677
3678
3679
3680
3681
3682
3683
3684
3685
3686
3687
3688
3689
3690
3691
3692
3693
3694
3695
3696
3697
3698
3699
3700
3701
3702
3703
3704
3705
3706
3707
3708
3709
3710
3711
3712
3713
3714
3715
3716
3717
3718
3719
3720
3721
3722
3723
3724

3725 3726 3727 3728 3729 3730
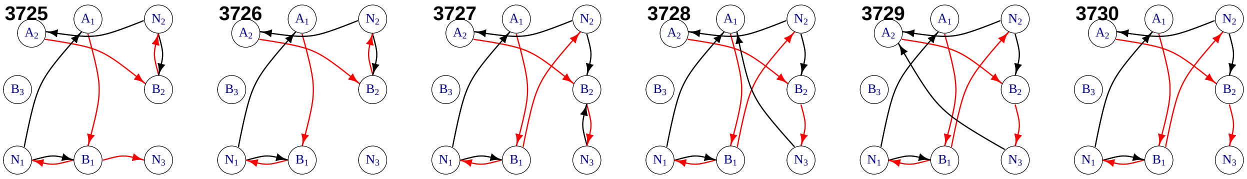

3731 3732 3733 3734 3735 3736
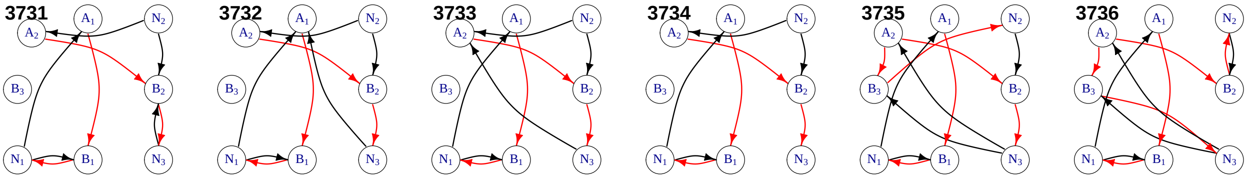

3737 3738 3739 3740 3741 3742
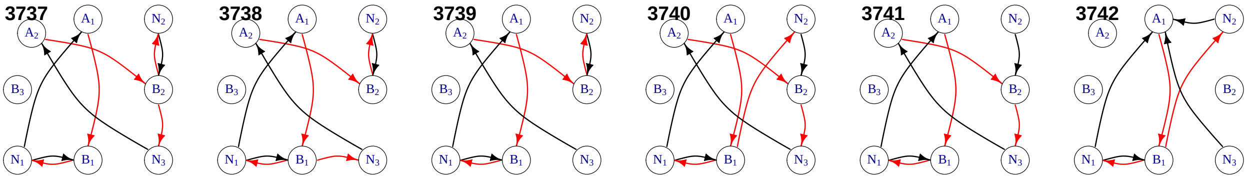

3743 3744 3745 3746 3747 3748
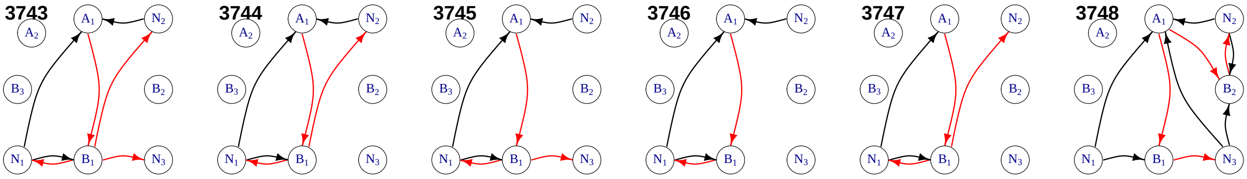

3749 3750 3751 3752 3753 3754
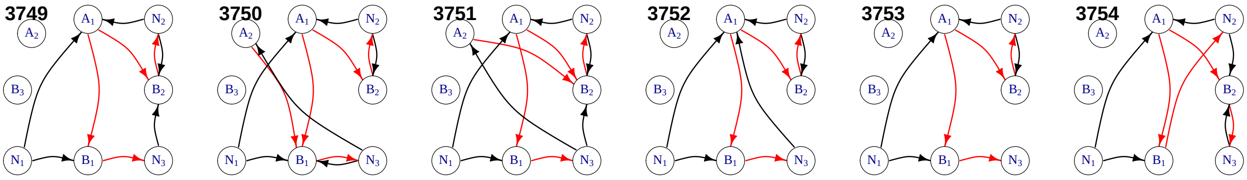

3755 3756 3757 3758 3759 3760
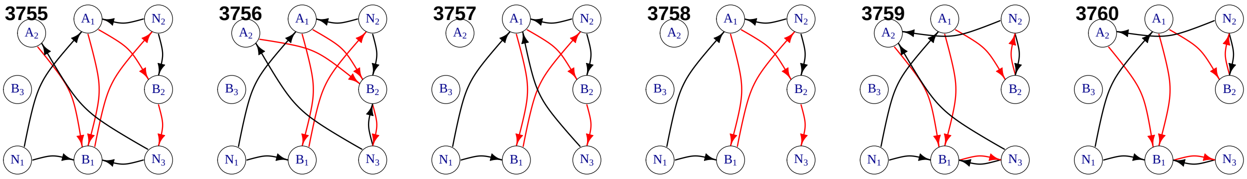

3761 3762 3763 3764 3765 3766
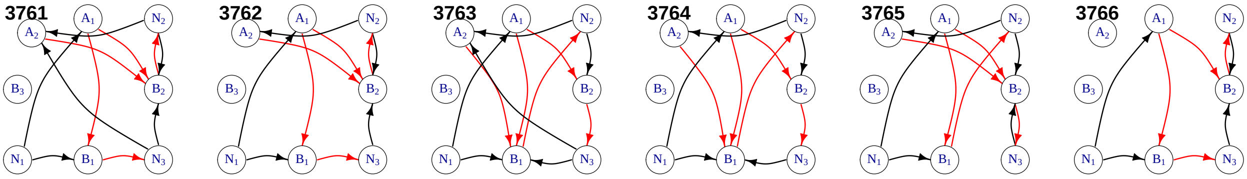

3767 3768 3769 3770 3771 3772
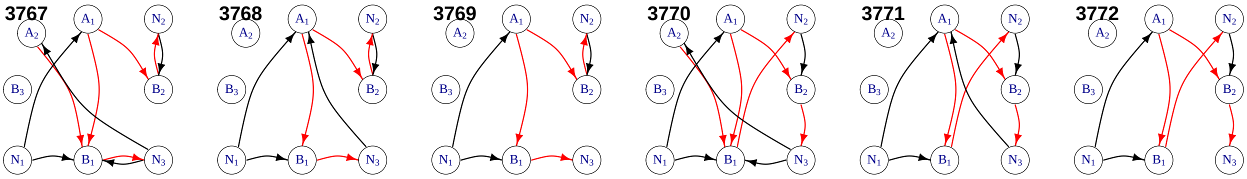

3773 3774 3775 3776 3777 3778
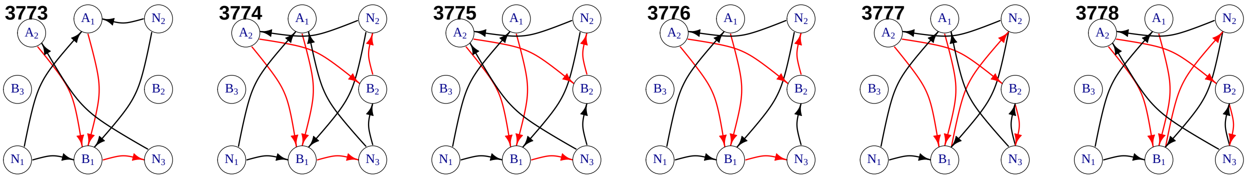

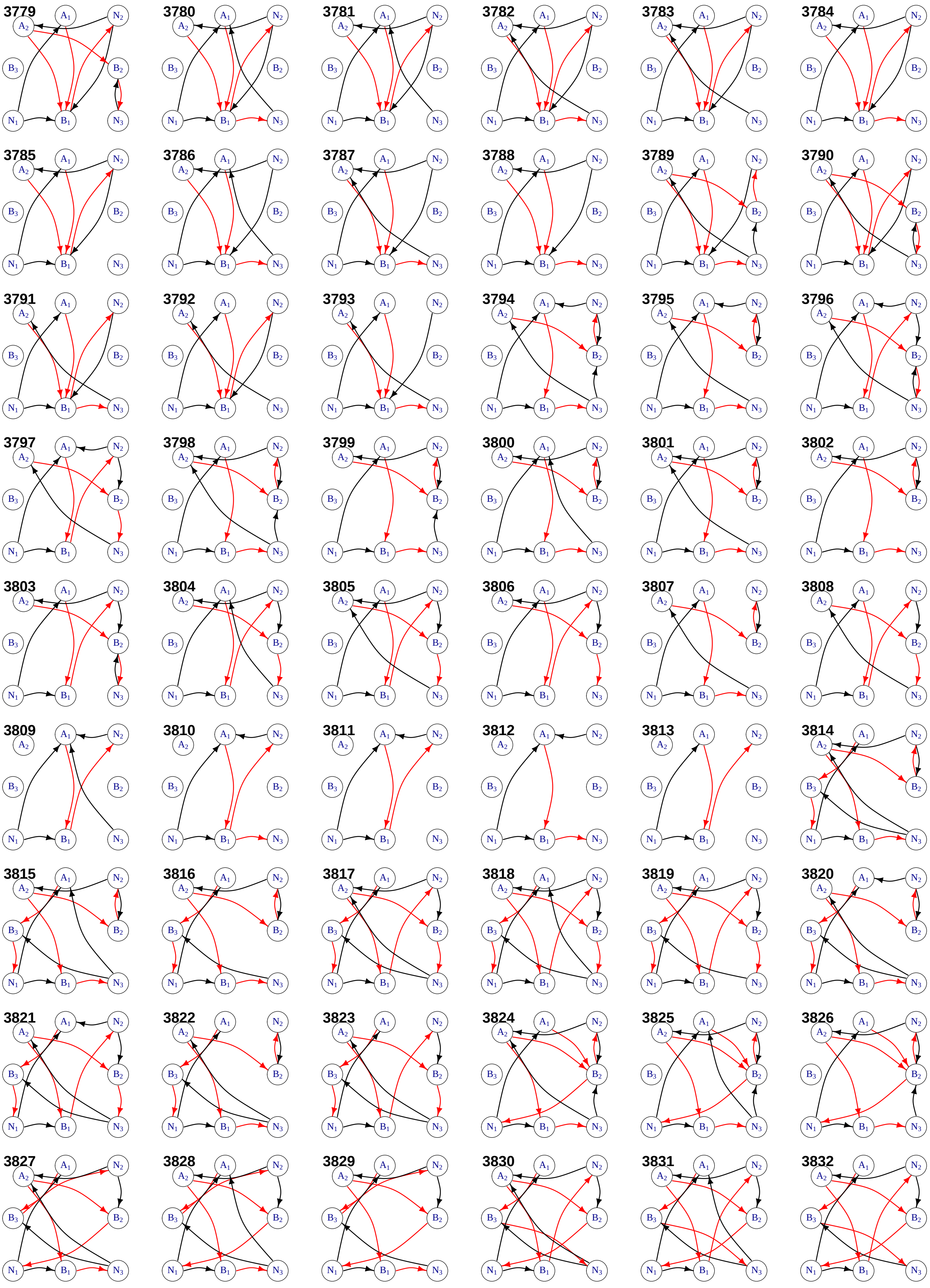
3779
3780
3781
3782
3783
3784
3785
3786
3787
3788
3789
3790
3791
3792
3793
3794
3795
3796
3797
3798
3799
3800
3801
3802
3803
3804
3805
3806
3807
3808
3809
3810
3811
3812
3813
3814
3815
3816
3817
3818
3819
3820
3821
3822
3823
3824
3825
3826
3827
3828
3829
3830
3831
3832

3833 3834 3835 3836 3837 3838

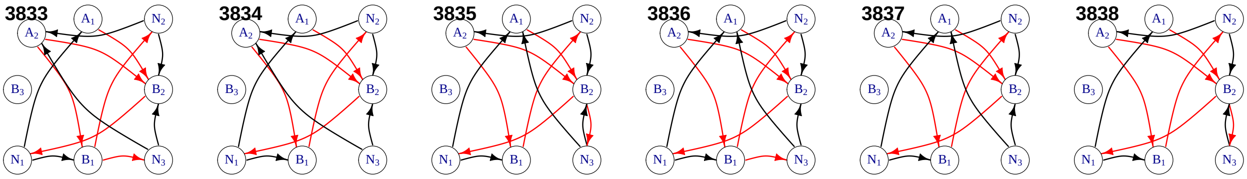

3839 3840 3841 3842 3843 3844

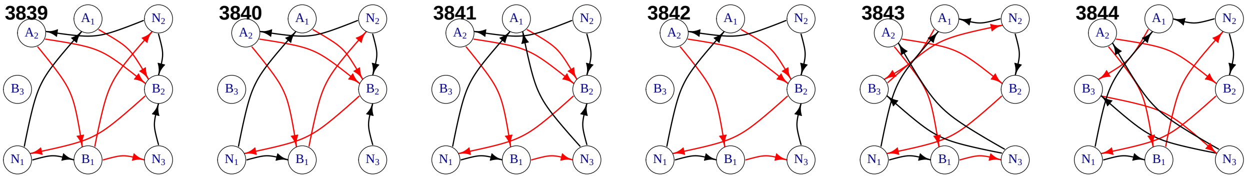

3845 3846 3847 3848 3849 3850

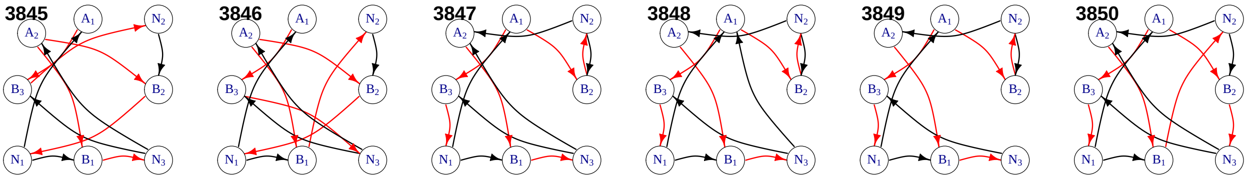

3851 3852 3853 3854 3855 3856

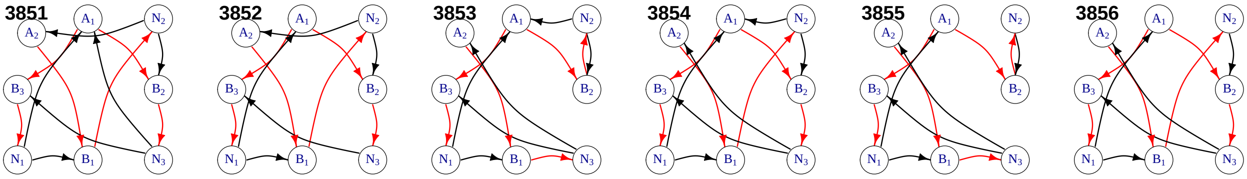

3857 3858 3859 3860 3861 3862

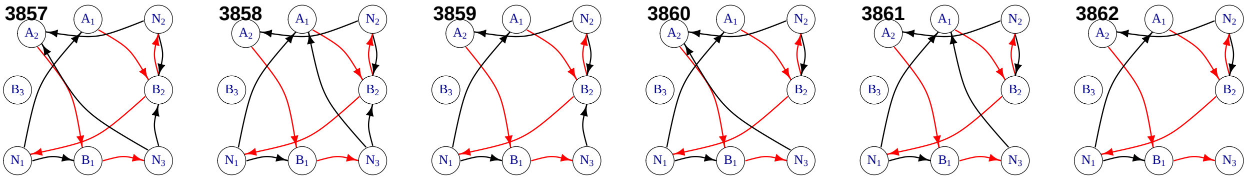

3863 3864 3865 3866 3867 3868

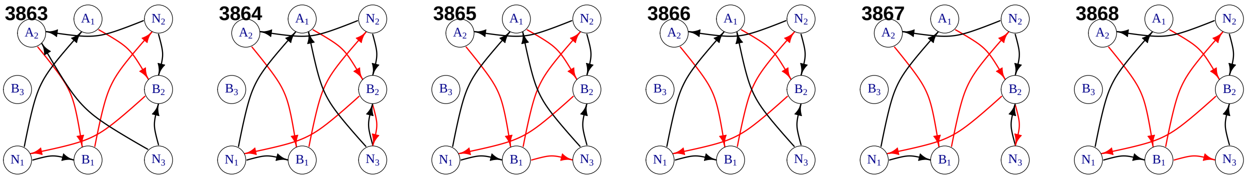

3869 3870 3871 3872 3873 3874

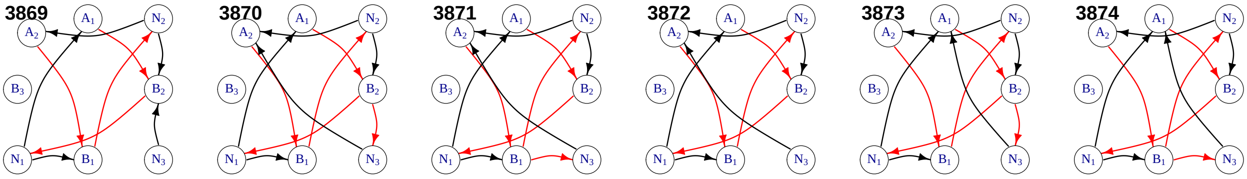

3875 3876 3877 3878 3879 3880

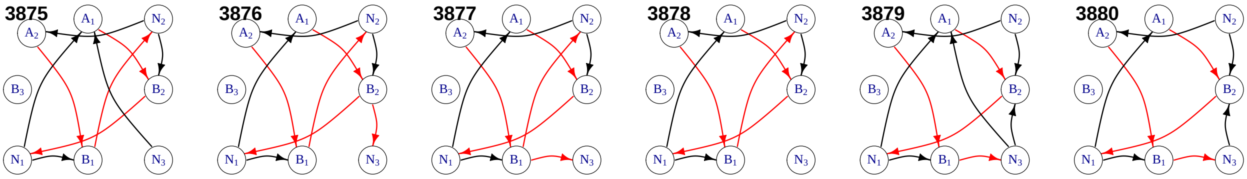

3881 3882 3883 3884 3885 3886

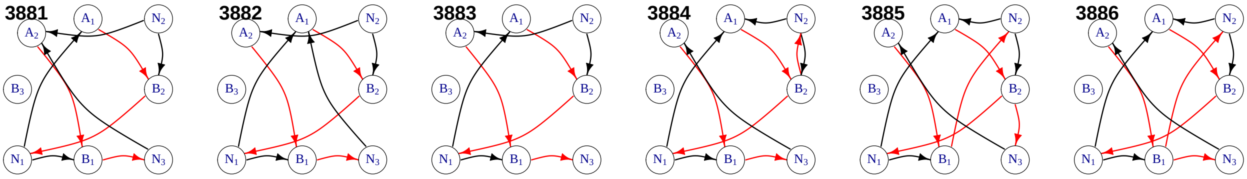

3887 3888 3889 3890 3891 3892

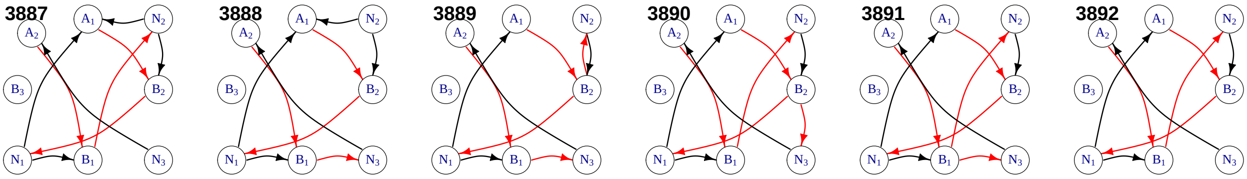

3893 3894 3895 3896 3897 3898

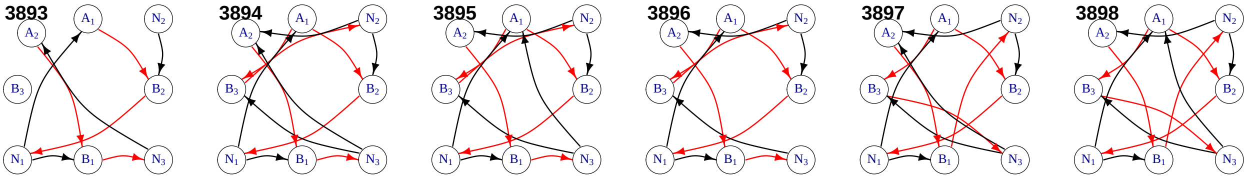

3899 3900 3901 3902 3903 3904

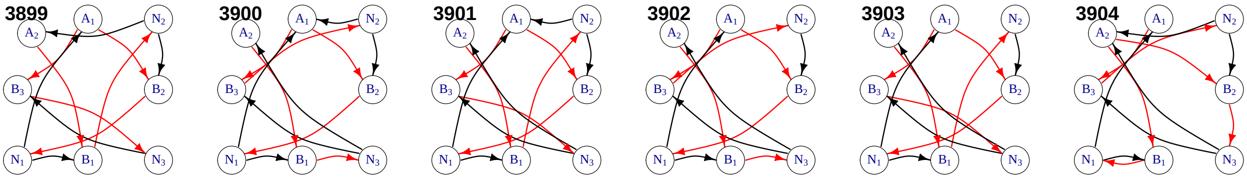

3905 3906 3907 3908 3909 3910

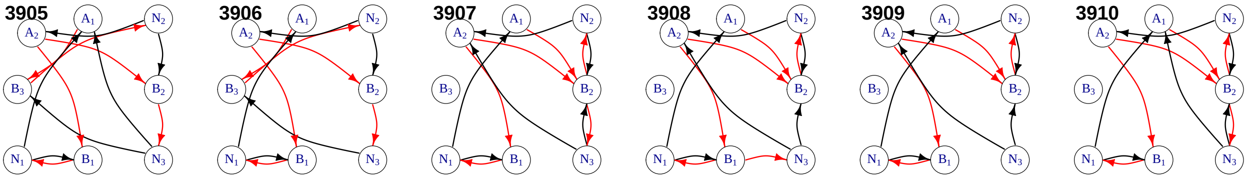

3911 3912 3913 3914 3915 3916

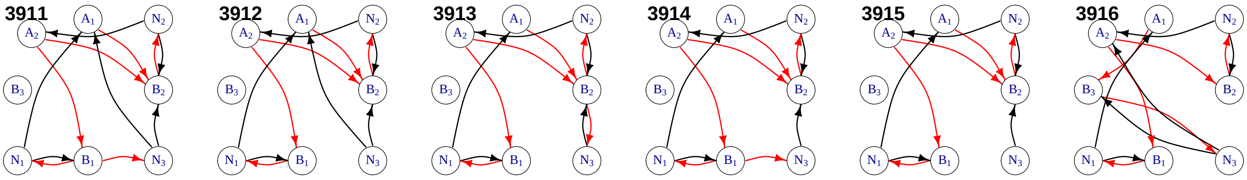

3917 3918 3919 3920 3921 3922

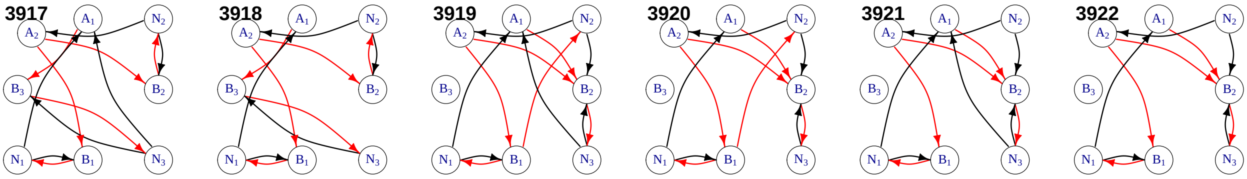

3923 3924 3925 3926 3927 3928

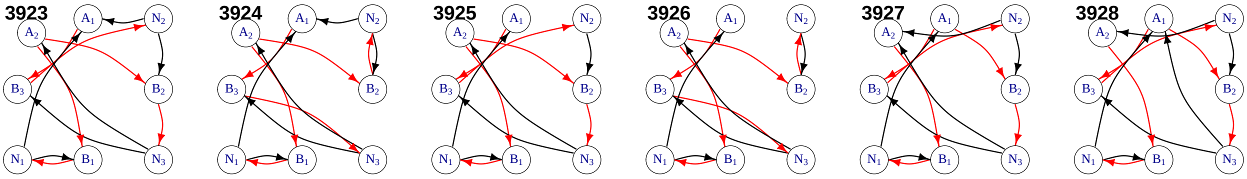

3929 3930 3931 3932 3933 3934

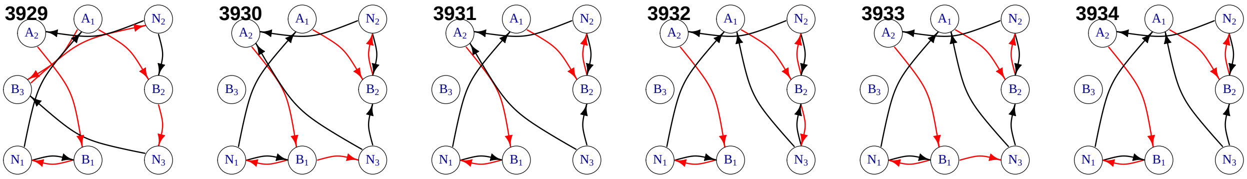

3935 3936 3937 3938 3939 3940

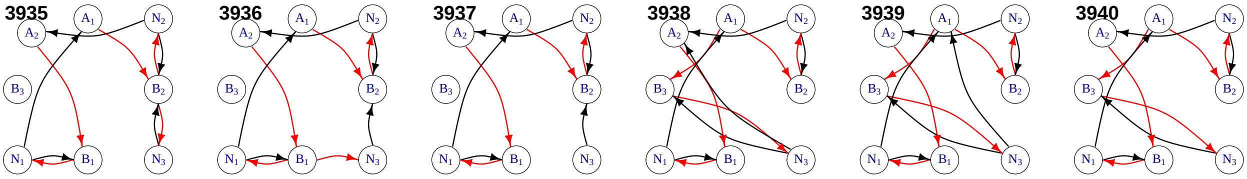

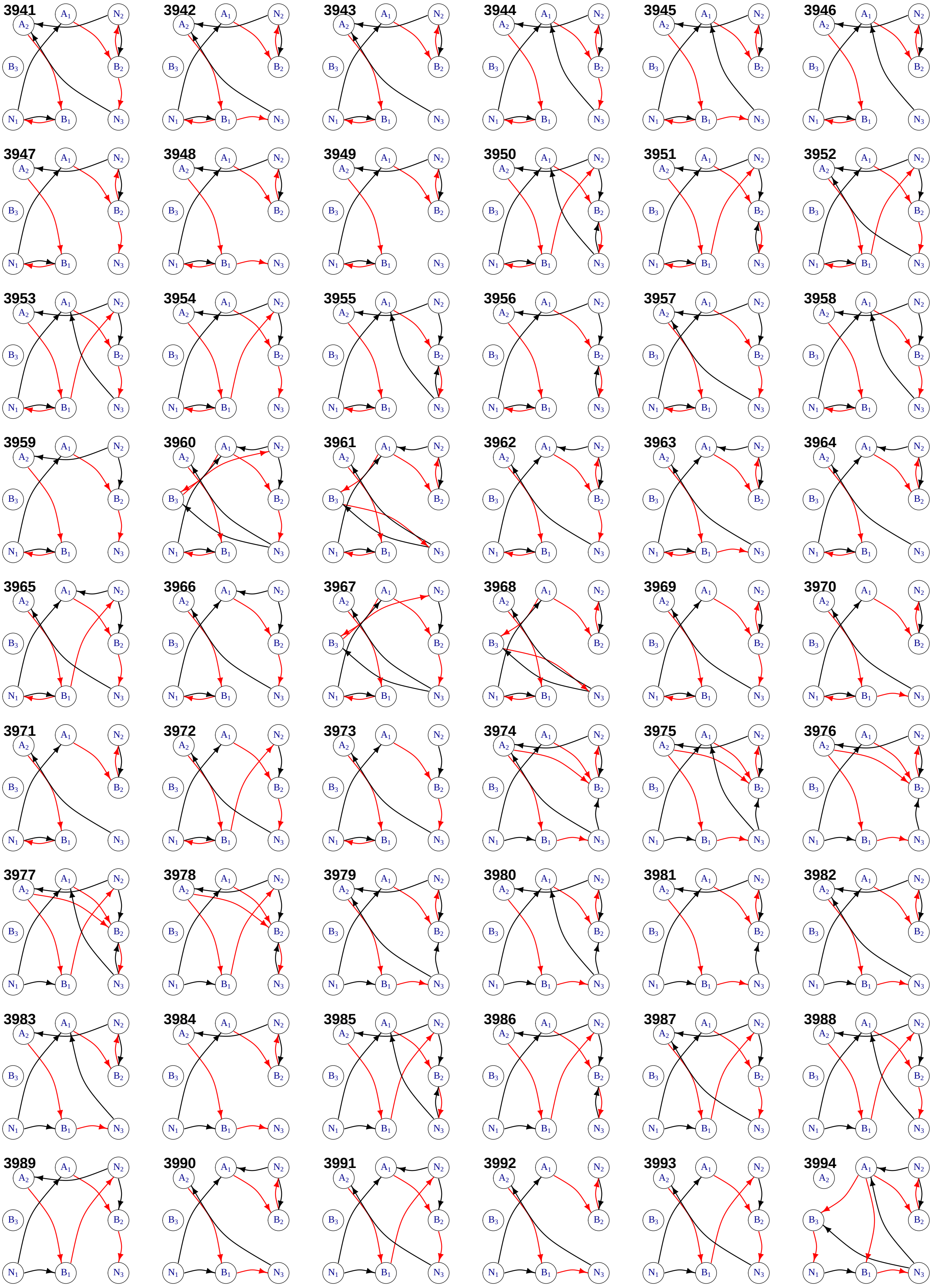
3941 3942 3943 3944 3945 3946
3947 3948 3949 3950 3951 3952
3953 3954 3955 3956 3957 3958
3959 3960 3961 3962 3963 3964
3965 3966 3967 3968 3969 3970
3971 3972 3973 3974 3975 3976
3977 3978 3979 3980 3981 3982
3983 3984 3985 3986 3987 3988
3989 3990 3991 3992 3993 3994

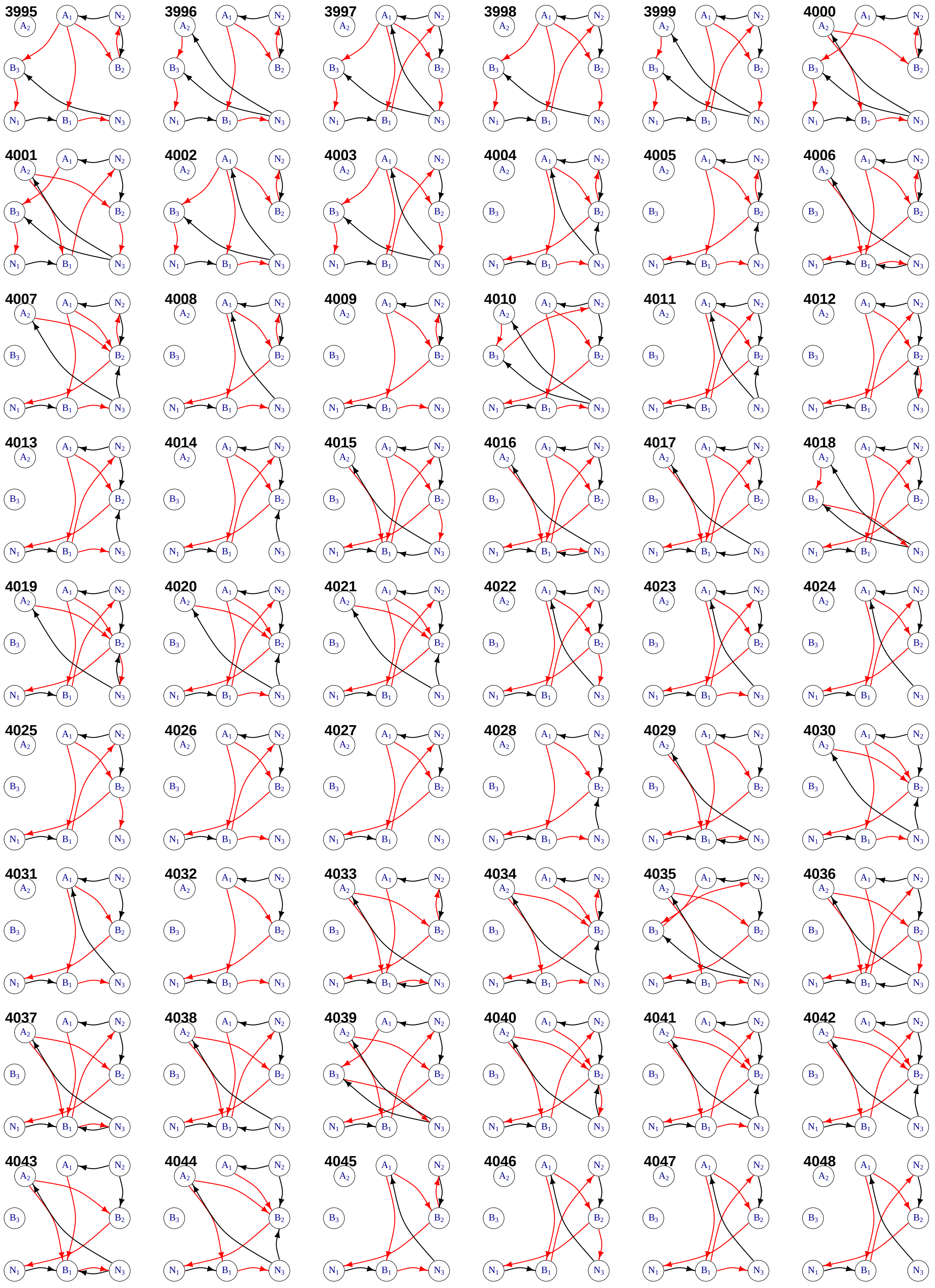
3995 3996 3997 3998 3999 4000
4001 4002 4003 4004 4005 4006
4007 4008 4009 4010 4011 4012
4013 4014 4015 4016 4017 4018
4019 4020 4021 4022 4023 4024
4025 4026 4027 4028 4029 4030
4031 4032 4033 4034 4035 4036
4037 4038 4039 4040 4041 4042
4043 4044 4045 4046 4047 4048

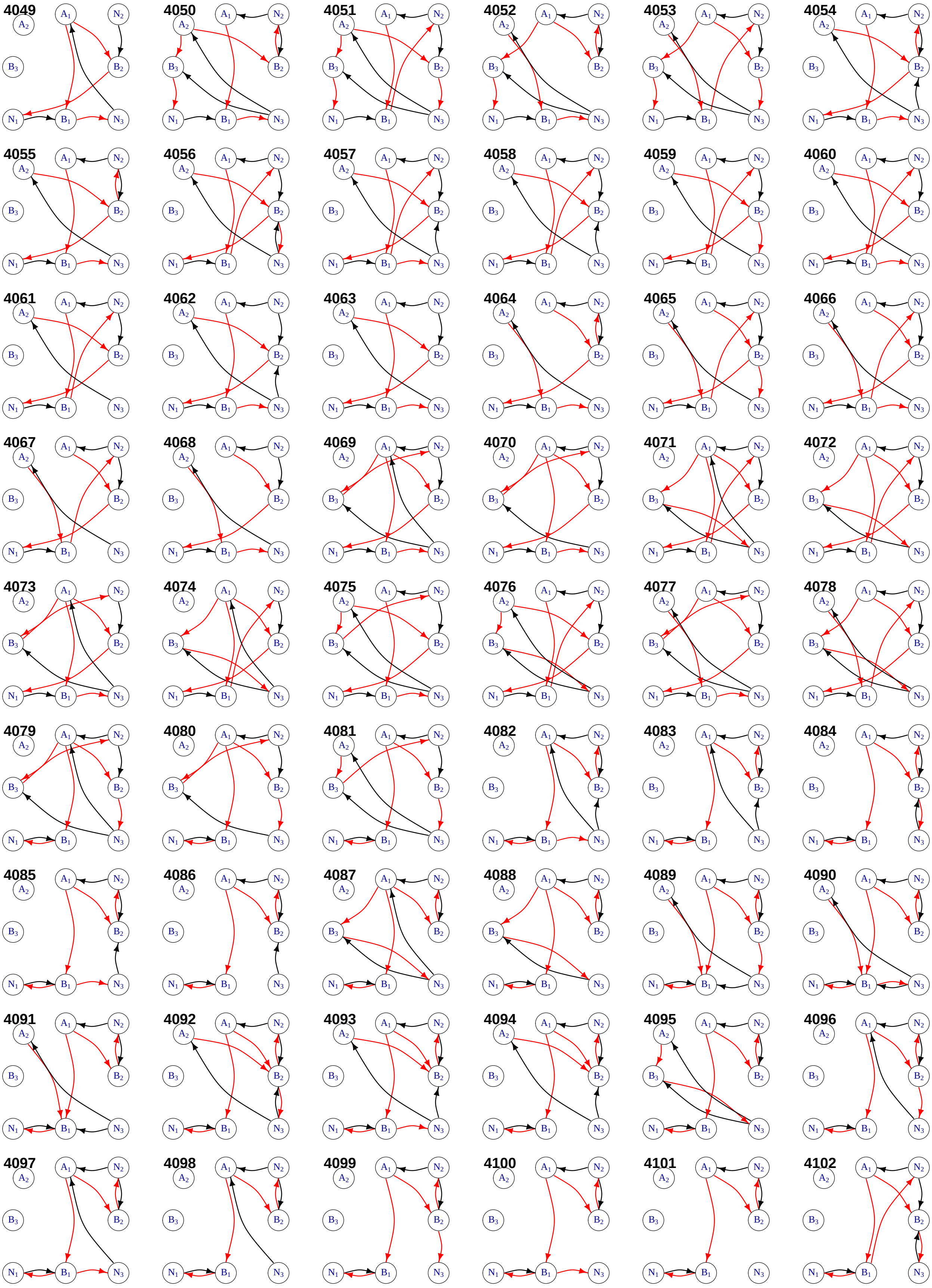
4049
4050
4051
4052
4053
4054
4055
4056
4057
4058
4059
4060
4061
4062
4063
4064
4065
4066
4067
4068
4069
4070
4071
4072
4073
4074
4075
4076
4077
4078
4079
4080
4081
4082
4083
4084
4085
4086
4087
4088
4089
4090
4091
4092
4093
4094
4095
4096
4097
4098
4099
4100
4101
4102

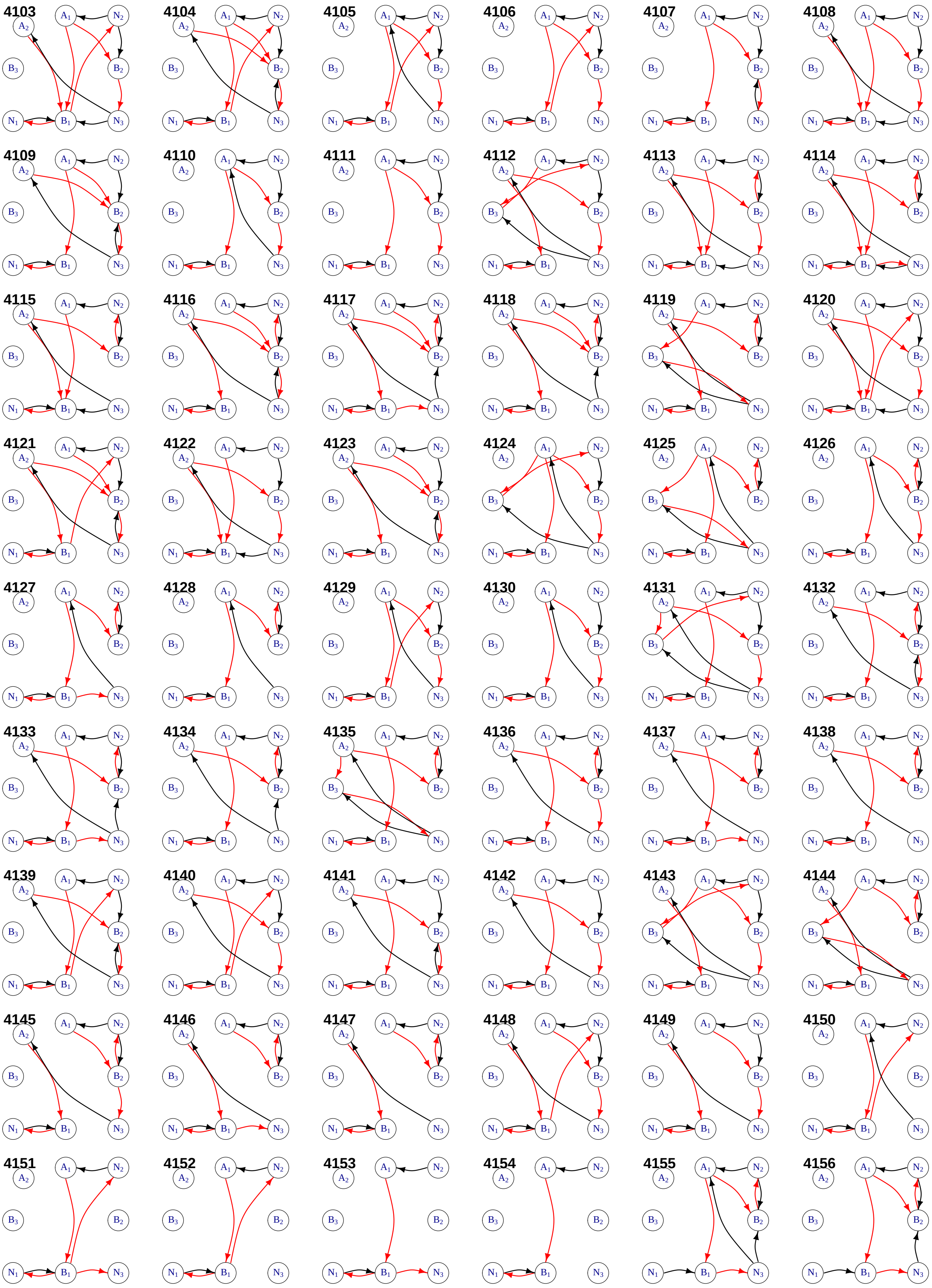
4103
4104
4105
4106
4107
4108
4109
4110
4111
4112
4113
4114
4115
4116
4117
4118
4119
4120
4121
4122
4123
4124
4125
4126
4127
4128
4129
4130
4131
4132
4133
4134
4135
4136
4137
4138
4139
4140
4141
4142
4143
4144
4145
4146
4147
4148
4149
4150
4151
4152
4153
4154
4155
4156

4157
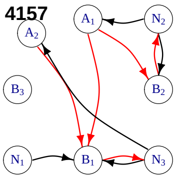

4158
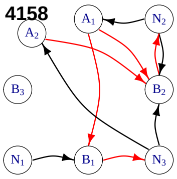

4159
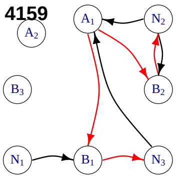

4160
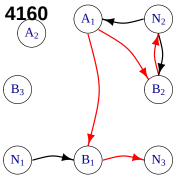

4161
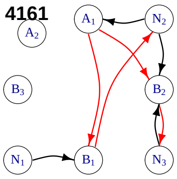

4162
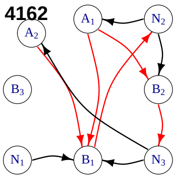

4163
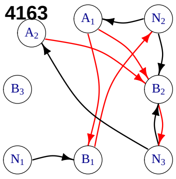

4164
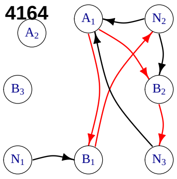

4165
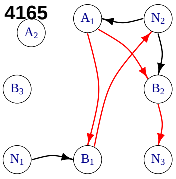

4166
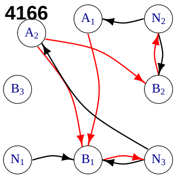

4167
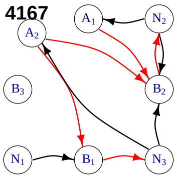

4168
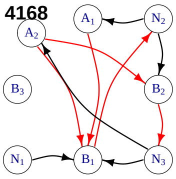

4169
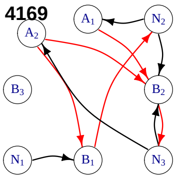

4170
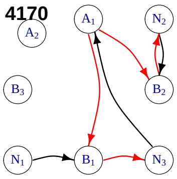

4171
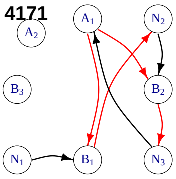

4172
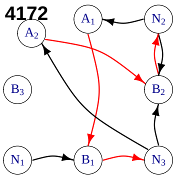

4173
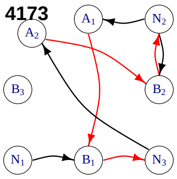

4174
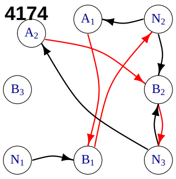

4175
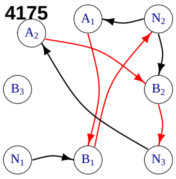

4176
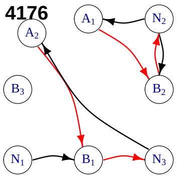

4177
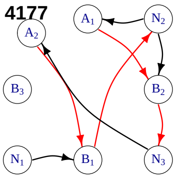

4178
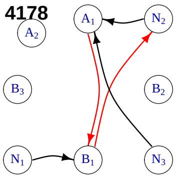

4179
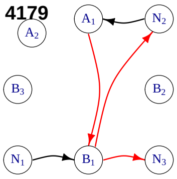

4180
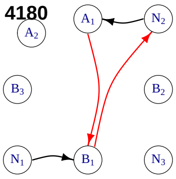

4181
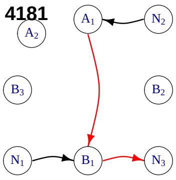

Supplement: Supplementary file 1 — Supplementary Information [file 41467_2020_20756_MOESM1_ESM.pdf]
