# Supplementary material for: Automated design of synthetic microbial communities
Source: Nat Commun. 2021 Jan 28;12:672. doi: 10.1038/s41467-020-20756-2 (PMC7844305; doi:10.1038/s41467-020-20756-2)
Supplement: Supplementary file 2 — Reporting Summary [file 41467_2020_20756_MOESM2_ESM.pdf]

nature research

Corresponding author(s): Prof. Chris P. Barnes
Last updated by author(s): Oct 8, 2020

Reporting Summary

Nature Research wishes to improve the reproducibility of the work that we publish. This form provides structure for consistency and transparency in reporting. For further information on Nature Research policies, see our Editorial Policies and the Editorial Policy Checklist.

Statistics

For all statistical analyses, confirm that the following items are present in the figure legend, table legend, main text, or Methods section.

n/a	Confirmed	
☐	☒	The exact sample size (n) for each experimental group/condition, given as a discrete number and unit of measurement
☐	☒	A statement on whether measurements were taken from distinct samples or whether the same sample was measured repeatedly
☒	☐	The statistical test(s) used AND whether they are one- or two-sided<br>*Only common tests should be described solely by name; describe more complex techniques in the Methods section.*
☒	☐	A description of all covariates tested
☐	☒	A description of any assumptions or corrections, such as tests of normality and adjustment for multiple comparisons
☐	☒	A full description of the statistical parameters including central tendency (e.g. means) or other basic estimates (e.g. regression coefficient) AND variation (e.g. standard deviation) or associated estimates of uncertainty (e.g. confidence intervals)
☒	☐	For null hypothesis testing, the test statistic (e.g. F, t, r) with confidence intervals, effect sizes, degrees of freedom and P value noted<br>*Give P values as exact values whenever suitable.*
☐	☒	For Bayesian analysis, information on the choice of priors and Markov chain Monte Carlo settings
☒	☐	For hierarchical and complex designs, identification of the appropriate level for tests and full reporting of outcomes
☐	☒	Estimates of effect sizes (e.g. Cohen's d, Pearson's r), indicating how they were calculated

Our web collection on statistics for biologists contains articles on many of the points above.

Software and code

Policy information about availability of computer code

Data collection

Data was generated using custom code that generates a model space and performs model selection via ABC SMC. Code is run with Python 3.6.11, C++11 and R 4.0. Intended to be run on HPC, and available on GitHub: https://github.com/ucl-cssb/AutoCD.

The data and code used for visualisation can be found at https://doi.org/10.5281/zenodo.4268341.

Figure 2a: Hierarchical clustering performed using Scipy. Plots made using Seaborn.
Figure 2b: Plots made using Seaborn.
Figure 2c: Density distributions made with R. Densities were calculated using library sm and plotted using ggisoband and ggplot2

Figure 3b: Non-negative matrix factorisation was performed using Scikit-learn. Plot made using Seaborn
Figure 3e: Plot made using Seaborn

Figure 4a: Hierarchical clustering performed using Scipy. Plots made using Seaborn.
Figure 4b: Plots made using Seaborn.

Figure 5a: Plots made using Seaborn.

Figure 6ab: Plots made using python-ternary
Figure 6c: Plots made using Seaborn

R requirements and versions used:
sm==2.2.5.6

ggisoband==0.0.0.9
ggplot2==3.3.0
gridExtra==2.3

C++ requirements and versions used:
Compiled with gcc 8.0
Boost ==1.67

Full list of Python requirements and versions used:
certifi==2020.6.20
chardet==3.0.4
colour==0.1.5
cycler==0.10.0
decorator==4.4.2
docopt==0.6.2
idna==2.9
joblib==0.15.1
kiwisolver==1.2.0
matplotlib==3.2.2
mpmath==1.1.0
networkx==2.4
netwulf==0.1.4
numpy==1.19.0
pandas==1.0.5
patsy==0.5.1
pipreqs==0.4.10
pyparsing==2.4.7
python-dateutil==2.8.1
python-ternary==1.0.7
pytz==2020.1
PyYAML==5.3.1
requests==2.24.0
scikit-learn==0.23.1
scipy==1.5.0
seaborn==0.10.1
simplejson==3.17.0
six==1.15.0
sklearn==0.0
statsmodels==0.11.1
sympy==1.6
threadpoolctl==2.1.0
urllib3==1.25.9
yarg==0.1.9

Data analysis

AutoCD code repository can be found at https://zenodo.org/record/4266261. The repository includes configuration files for the two- and three- strain experiments conducted in this study.

All code and data to recreate figures can be found at https://doi.org/10.5281/zenodo.4286040

For manuscripts utilizing custom algorithms or software that are central to the research but not yet described in published literature, software must be made available to editors and reviewers. We strongly encourage code deposition in a community repository (e.g. GitHub). See the Nature Research guidelines for submitting code & software for further information.

Data

Policy information about availability of data

All manuscripts must include a data availability statement. This statement should provide the following information, where applicable:

- Accession codes, unique identifiers, or web links for publicly available datasets
- A list of figures that have associated raw data
- A description of any restrictions on data availability

Data along with code to recreate figures is available at https://doi.org/10.5281/zenodo.4286040

Field-specific reporting

Please select the one below that is the best fit for your research. If you are not sure, read the appropriate sections before making your selection.

[x] Life sciences [ ] Behavioural & social sciences [ ] Ecological, evolutionary & environmental sciences

For a reference copy of the document with all sections, see nature.com/documents/nr-reporting-summary-flat.pdf

Life sciences study design

All studies must disclose on these points even when the disclosure is negative.

Sample size	ABC SMC population sizes were determined by the number that could sufficiently resolve posterior probabilities between models, and produce small enough error between different repeat experiments.
Data exclusions	No data was excluded for analysis
Replication	ABC SMC replicates were aggregated and split into three groups. Standard deviation between groups was used to confirm adequate representation of the posterior probability by showing sufficiently small distribution of posterior probabilities across the three groups. All three groups showed the trends discussed in the manuscript. Our replication of these experiments was therefore successful.
Randomization	Prior distributions were sampled from randomly.
Blinding	All samples from the prior distribution are performed randomly with no intervention by the investigators. Therefore all experiments are blinded.

Reporting for specific materials, systems and methods

We require information from authors about some types of materials, experimental systems and methods used in many studies. Here, indicate whether each material, system or method listed is relevant to your study. If you are not sure if a list item applies to your research, read the appropriate section before selecting a response.

Materials & experimental systems

n/a	Involved in the study	
☒	☐	Antibodies
☒	☐	Eukaryotic cell lines
☒	☐	Palaeontology and archaeology
☒	☐	Animals and other organisms
☒	☐	Human research participants
☒	☐	Clinical data
☒	☐	Dual use research of concern

Methods

n/a	Involved in the study	
☒	☐	ChIP-seq
☒	☐	Flow cytometry
☒	☐	MRI-based neuroimaging
